# 2-Oxoglutarate derivatives can selectively enhance or inhibit the activity of human oxygenases

**DOI:** 10.1038/s41467-021-26673-2

**Published:** 2021-11-10

**Authors:** Yu Nakashima, Lennart Brewitz, Anthony Tumber, Eidarus Salah, Christopher J. Schofield

**Affiliations:** 1 Chemistry Research Laboratory, Department of Chemistry and the Ineos Oxford Institute for Antimicrobial Research, University of Oxford, 12 Mansfield Road, OX1 3TA Oxford, UK; 2 Present Address: Institute of Natural Medicine, University of Toyama, 2630-Sugitani, 930-0194 Toyama, Japan

**Keywords:** Oxidoreductases, Mass spectrometry, Small molecules, X-ray crystallography

## Abstract

2-Oxoglutarate (2OG) oxygenases are validated agrochemical and human drug targets. The potential for modulating their activity with 2OG derivatives has not been explored, possibly due to concerns regarding selectivity. We report proof-of-principle studies demonstrating selective enhancement or inhibition of 2OG oxygenase activity by 2-oxo acids. The human 2OG oxygenases studied, factor inhibiting hypoxia-inducible transcription factor HIF-α (FIH) and aspartate/asparagine-β-hydroxylase (AspH), catalyze C3 hydroxylations of Asp/Asn-residues. Of 35 tested 2OG derivatives, 10 enhance and 17 inhibit FIH activity. Comparison with results for AspH reveals that 2OG derivatives selectively enhance or inhibit FIH or AspH. Comparison of FIH structures complexed with 2OG derivatives to those for AspH provides insight into the basis of the observed selectivity. 2-Oxo acid derivatives have potential as drugs, for use in biomimetic catalysis, and in functional studies. The results suggest that the in vivo activity of 2OG oxygenases may be regulated by natural 2-oxo acids other than 2OG.

## Abstract (web summary)

The human 2-oxoglutarate (2OG) oxygenases FIH and AspH are relevant drug targets. Here, the authors show that synthetic and naturally occurring 2OG derivatives can selectively modulate FIH and AspH activities, suggesting that these compounds may serve as a basis to develop 2OG oxygenase-targeting probes and drugs.

## Introduction

2-Oxoglutarate (2OG) and Fe(II)-dependent oxygenases couple substrate oxidation with 2OG decarboxylation to give succinate and CO_2_; they have important functions in human biology, e.g., in hypoxia signaling, extracellular matrix biosynthesis, lipid metabolism, and DNA/RNA damage repair^1^. Nature appears to have evolved complex mechanisms to regulate the activity of, at least, some of the human 2OG oxygenases, including sometimes by the use of small-molecules^2–6^. In cancer cells, normal regulation of 2OG oxygenase activity can be dysregulated, inter alia owing to elevated levels of tricarboxylic acid (TCA) cycle and related metabolites, such as (*R*)-2-hydroxyglutarate (2HG)^6–8^. The presence of 2HG has been correlated with ten–eleven-translocation (TET) and JmjC lysine-specific *N*^ε^-demethylases (JmjC KDMs) inhibition; in vitro studies revealed that 2HG inhibits these 2OG oxygenases in a 2OG-competitive manner^4,9^. By contrast with the extensive studies on 2HG- and TCA cycle intermediate-mediated inhibition of 2OG oxygenases, only one human metabolite other than 2OG itself, i.e., 2-oxoadipate (2OA), has been reported to enable catalysis by wild-type 2OG oxygenases by acting as a cosubstrate^10–13^.

Despite the importance of 2-oxo acids in biology, medicine, and synthetic chemistry^14–18^, few studies have been directed at identifying 2-oxo acids other than 2OA which retain the catalytic activity of human 2OG oxygenases. C4-alkyl-substituted 2OG derivatives have been investigated as alternative cosubstrates for human JmjC lysine-specific *N*^ε^-demethylase 4 A (KDM4A), however, activity was observed with active site mutated, but not wildtype, KDM4A^19^. 2OG analogs have been shown to restore the activity of clinically observed variants of phytanoyl-CoA 2-hydroxylase (PAHX), but do not enable wild-type PAHX catalysis^20^.

**Fig. 1:**
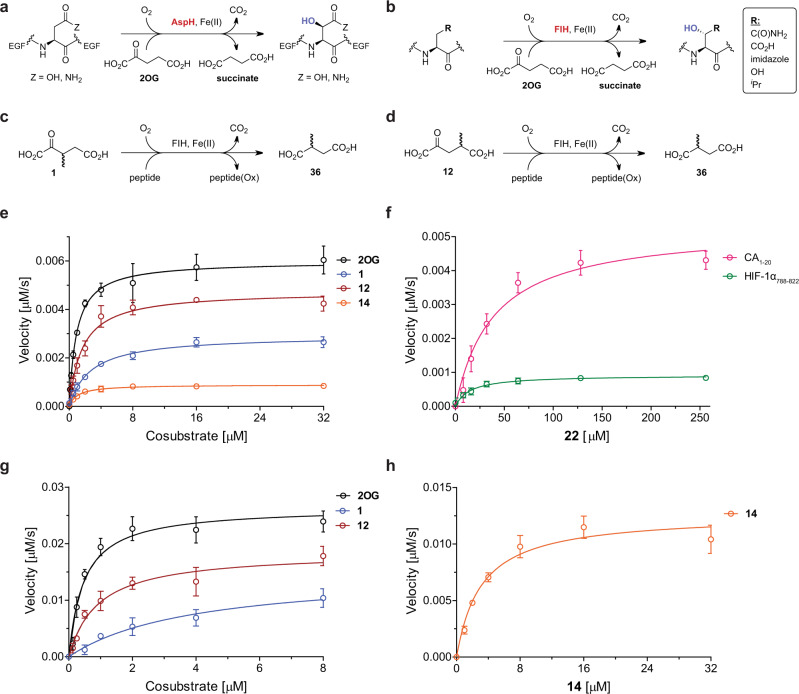
Comparison of AspH and FIH catalysis and steady-state kinetic parameters for FIH-catalyzed oxidative decarboxylation of 2OG derivatives from initial hydroxylation rates. **a** AspH and **b** FIH catalyze the post-translational C3 hydroxylation of Asn- and Asp-residues. FIH also catalyzes the oxidation of other residues, i.e., His-, Leu- and Ser-residues^41,42^; **c** and **d** FIH catalyzes the oxidative decarboxylation of **c** 3-methyl-2OG (**1**) and **d** 4-methyl-2OG (**12**) to give 2-methylsuccinate (**36**); **e** ${K}_{m}^{{{{{{\rm{app}}}}}}}$ of FIH for 2OG (black), **1** (blue), **12** (red), and **14** (orange) using HIF-1α_788–822_ as a substrate; **f** ${K}_{m}^{{{{{{\rm{app}}}}}}}$ of FIH for **22** using HIF-1α_788–822_ (green) or CA_1–20_ (pink) as a substrate; **g** ${K}_{m}^{{{{{{\rm{app}}}}}}}$ of FIH for 2OG (black), **1** (blue), and **12** (red) using CA_1–20_ as a substrate; **h** ${K}_{m}^{{{{{{\rm{app}}}}}}}$ of FIH for **14** (orange) using CA_1–20_ as a substrate. FIH assays were performed as described in the Methods section, data are shown as the mean of three independent runs (*n* = 3; mean ± standard deviation, SD). The peptide hydroxylation rates are shown in Supplementary Figs. 5 and 6, source data are provided as a Source Data file.

Recently, we developed an efficient synthesis of C3/C4-substituted 2OG derivatives, some of which are naturally occurring^21^. These 2OG derivatives were shown to enhance and/or inhibit the catalytic activity of the human 2OG oxygenase aspartate/asparagine-β-hydroxylase (AspH)^21^. AspH is a medicinal chemistry target for human cancer therapeutics and diagnostics^22–26^; it catalyzes the post-translational oxidation of Asp- and Asn-residues in specific macrocyclic disulfide isomers of epidermal growth factor-like domains (EGFDs) to give the β-erythro hydroxylated product (Fig. 1a)^27–31^.

Like AspH, human factor inhibiting the hypoxia-inducible transcription factor HIF-α (FIH) catalyzes the post-translational oxidation of Asp- and Asn-residues in a 2OG-dependent manner^32–34^, however, to give the β-threo hydroxylated product (Fig. 1b)^35^. FIH was initially identified as catalyzing hydroxylation of an Asn-residue in the C-terminal transactivation domain of HIF-α isoforms (Asn803 in HIF-1α), a reaction that suppresses HIF-mediated transcription^36^. FIH also accepts substrates other than HIF-α^37^; in particular, FIH catalyzes post-translational hydroxylations of Asp- and Asn-residues in ankyrin repeat domain proteins (e.g., iKB^38^, Notch^39^, and ASB4^40^). The current evidence suggests that FIH is biochemically more promiscuous with respect to its substrate requirements than AspH as it also catalyzes the hydroxylation of residues other than Asp and Asn (Fig. 1b)^41–43^.

The observation of AspH cosubstrate activity using C3/C4-substituted 2OG derivatives raises the possibility of selective enhancement or inhibition of 2OG oxygenases by 2-oxo acid derivatives. Here, we describe proof-of-principle studies with two isolated human 2OG oxygenases, i.e., FIH and AspH, that validate this concept. We chose to work with FIH and AspH since because both accept Asp- and Asn-residues as hydroxylation substrates, they represent a challenge in terms of selectivity. The combined crystallographic and mass spectrometry (MS)-based studies with FIH and AspH demonstrate the potential for selective enhancement or inhibition of specific 2OG oxygenase activity by 2-oxo acid derivatives.

## Results

### Synthetic 2OG derivatives are FIH cosubstrates

The ability of 35 synthetic C3/C4-substituted 2OG derivatives, which were prepared via a reported procedure (Supplementary Fig. 1 and Supplementary Methods)^21^, to sustain catalysis by purified recombinant FIH in the absence of 2OG was examined using an assay directly monitoring substrate depletion and product formation (i.e., a +16 Da mass shift) by solid-phase extraction (SPE) coupled to mass spectrometry (SPE-MS)^44,45^. Two peptides, based on the sequence of (1) the HIF-1α C-terminal transactivation domain fragment (i.e., HIF-1α_788–822_)^37^ and (2) a “consensus” FIH substrate ankyrin repeat sequence^46^ (i.e. CA_1–20_)^47^, were used as FIH substrates to test for potential substrate-dependent differences in terms of cosubstrate analog selectivity. Initial screening assays were performed in the absence of 2OG, but in the presence of high concentrations (330 μM) of the synthetic C3/C4-substituted 2OG derivatives to facilitate the identification of those 2OG derivatives which sustain FIH catalysis.

In general, FIH-catalyzed hydroxylation of CA_1–20_ appeared to be more efficient than of HIF-1α_788–822_ when using 2OG as the control cosubstrate (Table 1, entry 1). High levels of FIH substrate hydroxylation were observed for 3-methyl-2OG (**1**) and 4-methyl-2OG (**12**), both of which are natural products^48–51^, regardless of the substrate, indicating that these 2OG derivatives can efficiently replace 2OG (Table 1, entries 2 and 13). NMR studies reveal that FIH converts **1** and **12** into 2-methylsuccinate with a similar efficiency to which it converts 2OG into succinate (Fig. 1c–d, Supplementary Figs. 2–4). FIH-catalyzed substrate hydroxylation was clearly apparent using other 2OG derivatives, i.e., **2**, **3**, **13**-**16**, **22**, and **28** (Entries 3, 4, 14–16, 23, and 29); however, in all these cases, conversions were lower compared to 2OG, **1** or **12** (Table 1).

**Table 1:**
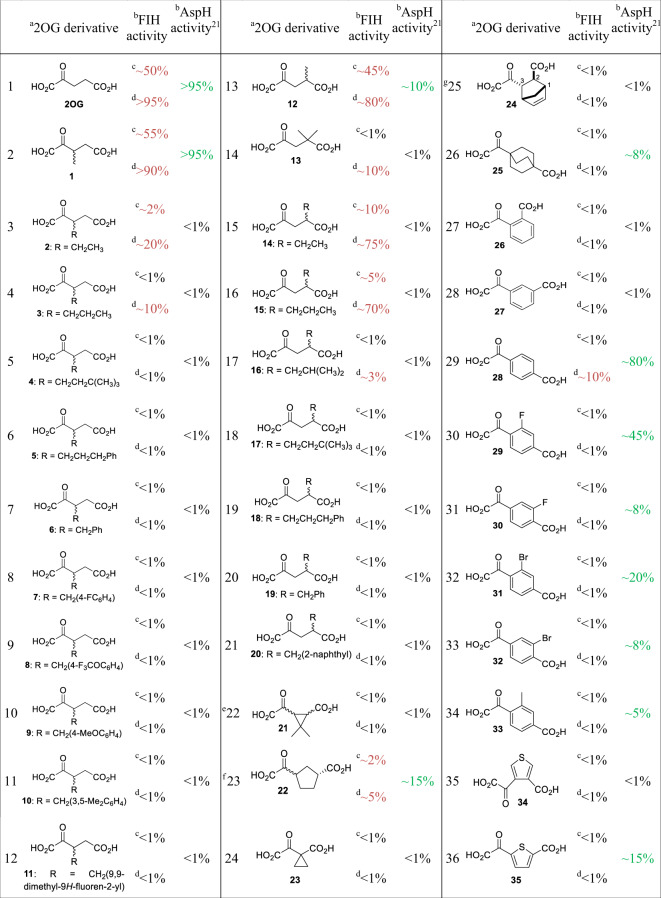
Synthetic C3/C4-substituted 2OG derivatives sustain FIH catalysis in the absence of 2OG.

^a^The synthetic C3/C4-substituted 2OG derivatives were prepared via a reported procedure (Supplementary Fig. 1)^21^, chiral 2OG derivatives were prepared as racemic mixtures. FIH and AspH activities for those 2OG derivatives acting as cosubstrates are in red and green, respectively.

^b^SPE-MS FIH turnover assays were performed using 0.15 μM FIH, 5.0 μM substrate peptide (HIF-1α_788–822_^37^ or CA_1–20_^47^), 50 μM Fe(II), 330 μM 2OG or 2OG derivative in buffer (50 mM Tris, 50 mM NaCl, pH 7.5, 20 °C). SPE-MS AspH turnover assay conditions^21^: 0.1 μM AspH, 2.0 μM substrate peptide (hFX-CP_101–119_), 50 μM Fe(II), 330 μM 2OG or 2OG derivative in reaction buffer (50 mM HEPES, pH 7.5, 20 °C). %-Substrate conversion is shown for *t* = 15 min.

^c^Using HIF-1α_788–822__._

^d^Using CA_1–20__._

^e^Mixture of racemic diastereomers, dr (*cis*:*trans*) = 2.5:1.

^f^Mixture of diastereomers, dr (*cis*:*trans*) = 1:1.

^g^(±)-(*2-exo,3-endo*)-diastereomer.

The results reveal that FIH activity decreases with both substrates with the increasing length of the 2OG C3/C4-alkyl substituent (i.e., Me > Et > Pr). The cosubstrate activity, albeit at low levels, for **22** and **28** is remarkable as their cyclic/aromatic carbon scaffolds deviate substantially from the acyclic 2OG carbon scaffold (Entries 23 and 29). Under these conditions, none of the analogs appeared to promote HIF-1α (HIF-1α_788–822_) hydroxylation relative to ankyrin (CA_1–20_) hydroxylation, suggesting the use of 2OG derivatives to modulate FIH activity with respect to particular substrates may be challenging (see below). All the other 2OG derivatives investigated did not show cosubstrate activity under the reaction conditions, in accord with the lack of FIH cosubstrate activity of the 2OG derivative **19**, reported using an LC-MS assay^52^.

Comparison of these initial results for FIH with those reported for AspH^21^ revealed the potential of selectively promoting FIH and/or AspH catalysis by using specific 2OG derivatives as cosubstrates. For example, the 2OG derivatives **2**, **3**, and **13**–**15** are efficient cosubstrates for FIH, but not for AspH, whereas **35** and **29**–**33** showed activity with AspH, but not with FIH (Table 1)^21^. Notably, 3-methyl-2OG (**1**) is an efficient cosubstrate for both FIH and AspH (Table 1, entry 2), whereas its isomer 4-methyl-2OG (**12**) is apparently more efficient in maintaining FIH compared with AspH activity (Table 1, entry 13). 4-Carboxyphenylglyoxylic acid (**28**) is an efficient AspH cosubstrate but shows only low FIH activity with CA_1–20_ and none with HIF-1α_788–822_ as a substrate (Table 1, entry 29). Note that our initial screening conditions differed when investigating FIH and AspH cosubstrate activity; i.e., the enzyme and substrate concentrations were different (0.15 μM and 5.0 μM for FIH; 0.1 μM and 2.0 μM for AspH) and different buffers were used (FIH: 50 mM Tris, 50 mM NaCl, pH 7.5; AspH: 50 mM HEPES, pH 7.5); these differences might, in part, reflect observed differences in activity with the 2OG derivatives. We, therefore, carried out more detailed kinetic FIH studies to better compare the results with those reported for AspH^21^.

### Kinetic analyses

To quantify FIH activity with the 2OG derivatives, kinetic analyses of selected compounds (i.e., 2OG, **1**, **12**, **14**, and **22**) were performed using HIF-1α_788–822_^37^ and CA_1–20_^47^ (Fig. 1e–h; Supplementary Figs. 5 and 6). Maximum velocities (${v}_{{{{{{\rm{max }}}}}}}^{{{{{{\rm{app}}}}}}}$) and Michaelis constants (${K}_{m}^{{{{{{\rm{app}}}}}}}$) were determined using 2OG as a control. The ${v}_{{{{{{\rm{max }}}}}}}^{{{{{{\rm{app}}}}}}}$-values reveal that the FIH-catalyzed hydroxylation of CA_1–20_ is faster than that of HIF-1α_788–822_ for all the cosubstrates analyzed, including 2OG (Table 2, entry 1).

**Table 2:** Comparison of the steady-state kinetic parameters of FIH for 2OG and the synthetic 2OG derivatives **1**, **12**, **14**, and **22** with those reported for AspH^a,b^.

^a^Mean of three independent runs (*n* = 3; mean ± SD).

^b^Maximum velocities (${v}_{{{{{{\rm{max }}}}}}}^{{{{{{\rm{app}}}}}}}$), Michaelis constants (${K}_{m}^{{{{{{\rm{app}}}}}}}$), turnover numbers (${k}_{{{{{{\rm{cat}}}}}}}^{{{{{{\rm{app}}}}}}}$), and specificity constant (${k}_{{{{{{\rm{cat}}}}}}}$/${K}_{m}$) were determined as described in the Methods section using 0.15 μM FIH and 5.0 μM HIF-1α_788–822_^37^ or CA_1–20_^47^ as substrates.

^c^Mixture of diastereomers, dr (*cis*:*trans*) = 1:1.

The FIH ${K}_{m}^{{{{{{\rm{app}}}}}}}$-values for 2OG are lower than those reported using different assays (25–110 μM)^37,53^, but in the range of those determined for other human 2OG oxygenases using SPE-MS assays (0.5–5 μM)^27,54,55^, likely reflecting the high sensitivity of SPE-MS assays. The FIH ${K}_{m}^{{{{{{\rm{app}}}}}}}$-value for 4-methyl-2OG (**12**) is similar to that of 2OG, indicating a similar affinity; by contrast, the FIH ${K}_{m}^{{{{{{\rm{app}}}}}}}$-value for **1**, the 3-methyl regioisomer of **12**, is higher (Table 2). Increasing the size of the alkyl substituent at C4 from methyl (**12**) to ethyl (**14**) results in a higher ${K}_{m}^{{{{{{\rm{app}}}}}}}$-value when using CA_1–20_, whereas only a marginal effect on its ${K}_{m}^{{{{{{\rm{app}}}}}}}$-value was observed when using HIF-1α_788–822_. In general, the FIH ${K}_{m}^{{{{{{\rm{app}}}}}}}$-values for 2OG, **1**, **12**, and **14** are in the same range (~0.5 to ~4.0 μM; Table 2, entries 1–4), whereas the ${K}_{m}^{{{{{{\rm{app}}}}}}}$-values are substantially higher for **22** (15 and 39 μM, respectively; Table 2, entry 5). All the determined ${K}_{m}^{{{{{{\rm{app}}}}}}}$-concentrations are in the range of reported physiological 2OG levels in human plasma (9–12 μM 2OG)^56^, but lower than reported 2OG concentrations in healthy cells (~1 mM)^57,58^.

An active site titration, performed using *N*-oxalyl-d-phenylalanine^52^ (NOFD) as an inhibitor, revealed the apparent quantitative activity of FIH (Supplementary Fig. 7). Based on the concentration of active sites, turnover numbers (catalytic constants, ${k}_{{{{{{\rm{cat}}}}}}}^{{{{{{\rm{app}}}}}}}$) and specificity constants (${k}_{{{{{{\rm{cat}}}}}}}$/${K}_{m}$) were calculated. The ${k}_{{{{{{\rm{cat}}}}}}}^{{{{{{\rm{app}}}}}}}$-values decrease in the order of 2OG > **12** > **1** > **14** ≥ **22** (Table 2). The FIH ${k}_{{{{{{\rm{cat}}}}}}}$/${K}_{m}$-values for the 2OG derivatives are higher when CA_1–20_ was used instead of HIF-1α_788–822_. The highest FIH ${k}_{{{{{{\rm{cat}}}}}}}$/${K}_{m}$-value was observed for 2OG when using CA_1–20_ (~383 mM^−1^ s^−1^; Table 2, entry 1), which is about eightfold higher than the value for 2OG when using HIF-1α_788–822_ (~48 mM^−1^ s^−1^; Table 2, entry 1) and at least one order of magnitude higher than the values observed for the 2OG derivatives investigated, with the notable exception of the ${k}_{{{{{{\rm{cat}}}}}}}$/${K}_{m}$-value with **12** using CA_1–20_, compared with which it is approximately threefold higher (~132 mM^−1^ s^−1^; Table 2, entry 3). The results reveal that the FIH ${k}_{{{{{{\rm{cat}}}}}}}$/${K}_{m}$-values for the 2OG derivatives vary, to some extent, with the nature of the substrate. Considering the high number of reported FIH substrates, this observation suggests that for some substrates, the ${k}_{{{{{{\rm{cat}}}}}}}$/${K}_{m}$-values of FIH for 2OG derivatives might actually be higher than for 2OG. They also reveal that the ${k}_{{{{{{\rm{cat}}}}}}}$/${K}_{m}$-values of FIH for the 2OG derivatives might be higher for a specific substrate (i.e., when using **12** and CA_1–20_; Table 2, entry 3) than the ${k}_{{{{{{\rm{cat}}}}}}}$/${K}_{m}$-values of FIH for the natural cosubstrate 2OG when using different substrates (i.e., HIF-1α_788–822_; Table 2, entry 1).

The FIH ${k}_{{{{{{\rm{cat}}}}}}}$/${K}_{m}$-values for the 2OG derivatives are in the range of those reported for AspH, which were also obtained using SPE-MS assays (Table 2)^21^. Notably, the FIH ${k}_{{{{{{\rm{cat}}}}}}}$/${K}_{m}$-value for 2OG with CA_1–20_ is about threefold higher than the one of AspH for 2OG (~383 and ~130 mM^−1^ s^−1^, respectively; Table 2). By contrast, the FIH ${k}_{{{{{{\rm{cat}}}}}}}$/${K}_{m}$-value for **1** with CA_1–20_ is an order of magnitude lower than that for AspH (~25 and ~296 mM^−1^ s^−1^, respectively; Table 2). The FIH ${k}_{{{{{{\rm{cat}}}}}}}$/${K}_{m}$-values for **22** are similar to the reported one of AspH for **28** (~1 mM^−1^ s^−1^; Table 2, entries 5 and 6)^21^, which, however, is not an efficient FIH cosubstrate.

### FIH and AspH competition studies

Comparisons of the effects of the synthetic 2OG derivatives on FIH catalysis with their reported effect on AspH catalysis^21^ indicated their potential to selectively enhance FIH or AspH activity. However, the FIH and AspH data were obtained under different conditions (different enzyme/(co-)substrate/cofactor concentrations, enzyme/substrate ratios, and buffers), possibly perturbing the results of their direct comparison. It is an advantage of MS-based turnover assays that the hydroxylation of different substrate peptides can be monitored in parallel in a single reaction vessel. Thus, the FIH-catalyzed hydroxylation of two different substrates as well as FIH- and AspH-catalyzed substrate hydroxylations were assessed simultaneously in the same vessel, complementing prior kinetic and turnover studies using a single isolated enzyme and substrate.

**Fig. 2:**
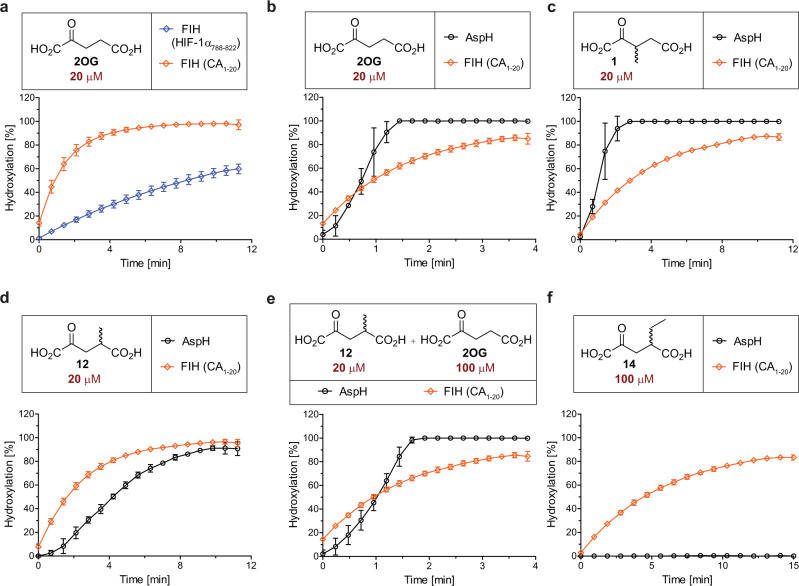
2OG derivatives selectively sustain the catalytic activities of 2OG oxygenases in vitro. Assays were performed as described in the Methods section using 0.15 μM of each 2OG oxygenase (diamond: FIH, circle: AspH) and 5.0 μM of each substrate (blue: HIF-1α_788–822_^37^; orange: CA_1–20_^47^; black: hFX-EGFD1_86–124_–4Ser^27,31^, AspH substrate) in the presence of 100 μM LAA, 10 μM FAS, and the indicated concentration of cosubstrate in buffer (50 mM Tris, 50 mM NaCl, pH 7.5). Mean of three independent runs (*n* = 3; mean ± SD). Measurement times were normalized to the first sample injection analyzed after the addition of the 2OG oxygenase to the Substrate Mixture (*t* = 0 min), by which time low levels of hydroxylation were manifest. Cosubstrates: **a** 20 μM 2OG; **b** 20 μM 2OG; **c** 20 μM **1**; **d** 20 μM **12**; **e** 20 μM **12** and 100 μM 2OG; **f** 100 μM **14**. Source data are provided as a Source Data file.

An equimolar mixture of the HIF-1α_788–822_^37^ (blue) and CA_1–20_^47^ (orange) peptides in a single reaction vessel was submitted to FIH-catalyzed modification at saturating 2OG and Fe(II) concentrations (Fig. 2a). The observed turnovers reflect the results of the kinetic experiments with individual substrates (Table 2). In agreement with an approximate eightfold difference observed in the FIH ${k}_{{{{{{\rm{cat}}}}}}}$/${K}_{m}$-values for 2OG, >90% conversion of CA_1–20_ was observed after 5 min, while only ~60% conversion of HIF-1α_788–822_ was observed after 12 min. This observation raises the possibility of the use of 2OG derivatives for selective enhancement of the oxidation of specific sets of substrates for a 2OG oxygenase.

Next, an equimolar amount of the FIH substrate CA_1–20_ and the AspH substrate hFX-EGFD1_86–124_-4Ser (5.0 μM each), the latter based on the sequence of the reported AspH substrate human coagulation factor X^27,31,59^, was submitted to equimolar amounts of FIH and AspH (0.15 μM each) in the presence of 2OG (20 μM) and Fe(II) (10 μM) in a single reaction vessel. The presence of two 2OG oxygenases did not result in substantial peptide ionization suppression and high-quality time course data were obtained: >95% AspH-catalyzed hFX-EGFD1_86–124_-4Ser hydroxylation was observed within about 1 min, whereas >80% FIH-catalyzed CA_1–20_ hydroxylation was observed after 4 min (Fig. 2b). By contrast to the analysis of the FIH and AspH kinetic parameters for 2OG with individual isolated enzymes (Table 2, entry 1), the results reveal that, when using 2OG as a cosubstrate under the tested conditions, AspH catalysis is more efficient than FIH catalysis. This might reflect the dimeric nature of FIH in solution^60^ and/or the different assay conditions used to obtain the kinetic parameters for isolated FIH and AspH; for example, a shorter substrate than the hFX-EGFD1_86–124_-4Ser peptide was used when determining the AspH kinetic parameters^21^.

In a similar single vessel experiment, an approximately twofold decrease in FIH efficiency was observed when 2OG was substituted for **1** (20 μM), keeping other reaction parameters same, i.e., >80% CA_1–20_ hydroxylation was observed after about 8 min rather than 4 min (Fig. 2c), whereas no detrimental effect on AspH catalysis was observed (>95% hFX-EGFD1_86–124_-4Ser hydroxylation within ~2 min Fig. 2c), in agreement with the kinetics of the individual enzymes (Table 2).

As the kinetic data with isolated FIH and AspH suggests (Table 2), using a saturating concentration of **12** (20 μM) as a cosubstrate instead of 2OG or **1**, reversed the reactivity profile (Fig. 2d). Thus, with **12**, FIH catalysis was more efficient than AspH catalysis, reaching ~90% CA_1–20_ hydroxylation after ~5 min, whereas AspH-catalyzed hFX-EGFD1_86–124_-4Ser hydroxylation reached ~90% after ~10 min in the same reaction vessel. Interestingly, the presence of both 2OG (100 μM) and **12** (20 μM) in the same reaction vessel reversed this effect, i.e., AspH catalysis is faster than FIH catalysis (Fig. 2e). Complete AspH-catalyzed hydroxylation of hFX-EGFD1_86–124_-4Ser was observed after ~2 min, which is only marginally slower than in the absence of any **12** (Fig. 2b). The FIH-catalyzed hydroxylation of CA_1–20_ was not affected; it reaches ~90% conversion in the presence of saturating 2OG or **12** concentrations, and also in the presence of both cosubstrates (Fig. 2e).

Comparing FIH and AspH activities in a single reaction vessel reveals that only FIH-catalyzed hydroxylation is observed when using 2OG derivative **14** (100 μM) in the absence of 2OG (Fig. 2f). However, FIH catalysis was less efficient for **14** than for 2OG, **1** or **12**, which were used at lower concentrations (20 μM). AspH-catalyzed hydroxylation of hFX-EGFD1_86–124_-4Ser was not observed, in agreement with the reported lack of cosubstrate activity with **14** (Table 1)^21^. Similarly, selective enhancement of AspH activity was observed without 2OG using the phenyl-ring containing 2OG derivative **28** (Supplementary Fig. 8), which showed negligible amounts of cosubstrate activity for FIH (Table 1).

### Crystallography

**Fig. 3:**
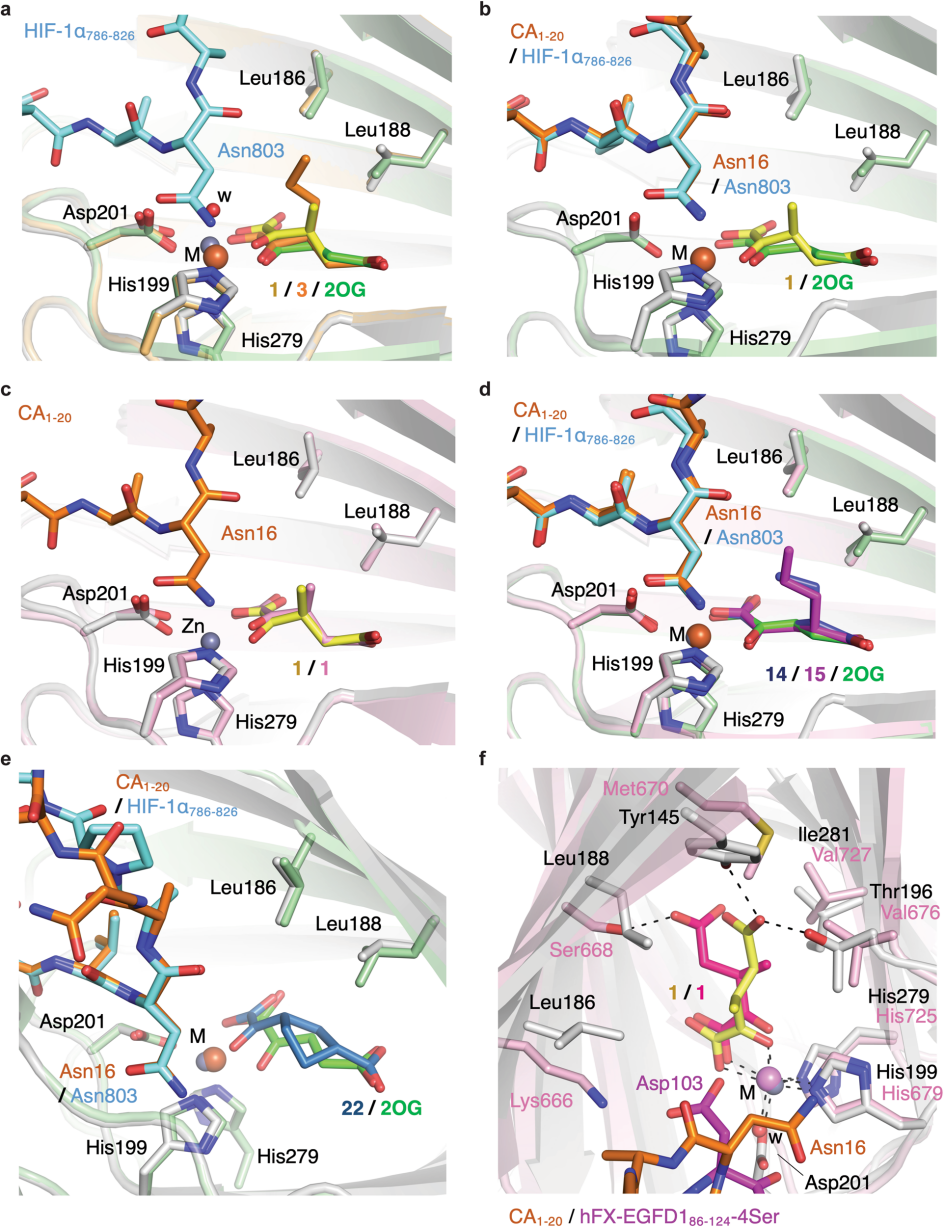
The synthetic 2OG derivatives 1, 3, 14, 15, and 22 bind FIH similarly to 2OG. **a** Superimposition of views from the FIH:**1** (FIH: gray; Zn ion: lavender blue; carbon-backbone of **1**: yellow) and FIH:**3** (FIH: light orange; Zn ion: lavender blue; carbon-backbone of **3**: orange; w: water) structures with a view from the reported FIH:2OG:HIF-1α_786–826_ structure (FIH: pale green; Fe ion: orange; carbon-backbone of 2OG: green; carbon-backbone of HIF-1α_786–826_: cyan; PDB ID: 1H2L^61^); **b** superimposition of a view from the FIH:**1**:CA_1–20_ structure (FIH: gray; Zn ion: lavender blue; carbon-backbone of **1**: yellow; carbon-backbone of CA_1–20_: orange) with one from the reported FIH:2OG:HIF-1α_786–826_ structure (FIH: pale green; Fe ion: orange; carbon-backbone of 2OG: green; carbon-backbone of HIF-1α_786–826_: cyan; PDB ID: 1H2L^61^); **c** superimposition of a view from the FIH:**1** structure (FIH: light pink; Zn ion: lavender blue; carbon-backbone of **1**: pink) with one from the FIH:**1**:CA_1–20_ structure (FIH: gray; Zn ion: lavender blue; carbon-backbone of **1**: yellow; carbon-backbone of CA_1–20_: orange); **d** superimposition of views from the FIH:**14**:CA_1–20_ (FIH: gray; Zn ion: lavender blue; carbon-backbone of **14**: blue; carbon-backbone of CA_1–20_: orange) and FIH:**15**:CA_1–20_ (FIH: light pink; Zn ion: lavender blue; carbon-backbone of **15**: magenta; carbon-backbone of CA_1–20_: orange) structures with a view from the reported FIH:2OG:HIF-1α_786–826_ structure (FIH: pale green; Fe ion: orange; carbon-backbone of 2OG: green; carbon-backbone of HIF-1α_786–826_: cyan; PDB ID: 1H2L^61^); **e** superimposition of a view from the FIH:**22**:CA_1–20_ structure (FIH: gray; Zn ion: lavender blue; carbon-backbone of **22**: sky blue; carbon-backbone of CA_1–20_: orange) with one from the reported FIH:2OG:HIF-1α_786–826_ structure (FIH: pale green; Fe ion: orange; carbon-backbone of 2OG: green; carbon-backbone of HIF-1α_786–826_: cyan; PDB ID: 1H2L^61^); **f** superimposition of a view from the FIH:**1**:CA_1–20_ structure (FIH: gray; Zn ion: lavender blue; carbon-backbone of **1**: yellow; carbon-backbone of CA_1–20_: orange) with one from the reported AspH:**1**:hFX-EGFD1_86–124_-4Ser structure (AspH: light pink; Mn ion: violet; carbon-backbone of **1**: hot pink; carbon-backbone of hFX-EGFD1_86–124_-4Ser: magenta; w: water; PDB ID: 6YYX^21^). Information on source data is provided in the Data availability section.

Crystallographic studies were initiated to investigate the binding of the 2OG derivatives to FIH and to compare the results with those reported for AspH^21^. Initially, FIH was crystallized in the presence of Zn(II), as a substitute for Fe(II), and in the presence of the C3-alkyl-substituted 2OG derivatives **1** and **3**. In the absence of substrate, the FIH complexes crystallized in the *P*4_1_2_1_2 space group (FIH:**1**, 2.29 Å resolution; FIH:**3**, 2.18 Å resolution; Supplementary Figs. 9–12). The structures were solved by molecular replacement (MR) using a reported FIH structure (PDB ID: 1H2K)^61^ as a search model. The superimposition of both structures with the reported FIH:2OG:HIF-1α_786–826_ structure (PDB ID: 1H2L)^61^ reveals that the conformation of 2OG does not substantially change upon introducing substituents at C3, albeit the conformation of the C3–C4 ethylene unit of **1** and **3** slightly differs from that of 2OG (Fig. 3a). The C3-alkyl substituents apparently align to interact with the hydrophobic sidechains of Leu186 and Leu188.

FIH co-crystallized with **1**, Zn, and CA_1–20_ in the *P*4_1_2_1_2 space group (FIH:**1**:CA_1–20_, 2.01 Å resolution; Supplementary Fig. 13). All FIH:substrate structures were solved by MR using a reported FIH structure (PDB ID: 1H2K)^61^ as a search model. Superimposition of the FIH:**1**:CA_1–20_ structure with the reported FIH:2OG:HIF-1α_786–826_ (PDB ID: 1H2L; Fig. 3b)^61^ and the FIH:**1** structures (Fig. 3c) reveals that the conformation of **1** does not substantially change upon substrate binding (Supplementary Fig. 14). The nature of the FIH substrate does not seem to influence the conformation of **1**, as revealed by co-crystallization with a fragment of the ankyrin repeat-containing validated human FIH substrate tankyrase-2^62^ (FIH:**1**:TNKS2_691–710_; 2.01 Å resolution; Supplementary Figs. 15–17).

FIH was also crystallized in the presence of CA_1–20_ and the C4-alkyl-substituted 2OG derivatives **14** and **15** (FIH:**14**:CA_1–20_, 2.21 Å resolution; FIH:**15**:CA_1–20_, 1.76 Å resolution; Supplementary Figs. 18–21). The conformations of **14** and **15** were very similar to that of 2OG with their C4-alkyl substituents facing towards the hydrophobic Leu186 and Leu188 sidechains (Fig. 3d), as observed for the C3-alkyl 2OG derivatives. The observation that the conformations of C3- and C4-alkyl 2OG derivatives are very similar to that of 2OG in complex with FIH is in agreement with their similar ${K}_{m}^{{{{{{\rm{app}}}}}}}$-values (Table 2). The difference in ${k}_{{{{{{\rm{cat}}}}}}}^{{{{{{\rm{app}}}}}}}$ is the major factor for the observed differences in the ${k}_{{{{{{\rm{cat}}}}}}}$/${K}_{m}$-values for **14** and 2OG; the lower ${k}_{{{{{{\rm{cat}}}}}}}^{{{{{{\rm{app}}}}}}}$ for **14** may reflect the lower conformational/rotational flexibility of the substituted 2OG derivatives due to stronger hydrophobic interactions with the Leu186 and Leu188 sidechains and/or the enhanced binding to and thus slower release of the corresponding alkyl-substituted succinate reaction product from FIH.

In the FIH:**22**:CA_1–20_ structure (1.75 Å resolution; Supplementary Figs. 22–23), **22** occupies a different conformation than that observed for 2OG, possibly to compensate for its rigid cyclic carbon-backbone (Fig. 3e). The FIH ${K}_{m}^{{{{{{\rm{app}}}}}}}$ for **22** reflects this conformational difference as it is about tenfold higher compared with the ${K}_{m}^{{{{{{\rm{app}}}}}}}$ for 2OG, **1**, and **14**, regardless of the substrate employed (Table 2), indicative of a lower affinity of FIH for **22**.

The hydrophobic interactions of the C3/C4-alkyl 2OG derivatives with the Leu186 and Leu188 sidechains in FIH resemble those of the C3/C4-alkyl-substituted 2OG derivatives with the Met670, Val676, and Val727 sidechains in AspH structures (Fig. 3f and Supplementary Figs. 24–25)^21^. In addition to differences in the 2OG C5 carboxylate binding motif, the interaction of aliphatic C3/C4-substituents of the 2OG derivatives with hydrophobic residues and/or hydrophobic pockets (as observed for AspH) in the active sites of 2OG oxygenases, appears to influence the conformation of the 2OG derivatives. Although the C3/C4-alkyl 2OG derivatives were used as racemic mixtures, the crystallographic analyses reveal that, at least predominantly, the (*S*)-enantiomers of the 2OG derivatives bind to FIH, potentially owing to their ability to engage in more efficient hydrophobic interactions with the hydrophobic Leu186 and Leu188 sidechains (Supplementary Figs. 10, 12, 14, 16, 19, and 21). A similar observation has been made for AspH, however, in this case, the (*R*)-enantiomers of the C3/C4-alkyl-substituted 2OG derivatives engage in hydrophobic interactions with AspH^21^. Interestingly, the unusual geometry of the AspH Fe(II)-binding site^21,31^, i.e., only two AspH histidine residues coordinate the Fe(II) cofactor (e.g., H_679_XG_681_…H_725_) rather than the typical triad of ligands (HXD/E…H) found in the active site of other human 2OG hydroxylases including FIH (H_199_XD_201_…H_279_)^1^, appears not to directly affect 2OG derivative binding to AspH nor AspH/FIH cosubstrate selectivity based on the superimposition of AspH and FIH substrate structures complexed with **1** (Fig. 3f).

### Synthetic 2OG derivatives inhibit FIH

To further investigate the utility of the 35 synthetic C3/C4-substituted 2OG derivatives for selectively modulating FIH or AspH activity, their inhibitory effect on FIH activity was investigated^44,45^. As the crystallographic studies revealed that the conformations of the CA_1–20_ and HIF-1α_786–826_ peptides align well at the FIH active site (Fig. 3b, d, and e), i.e. the predicted binding site of the 2OG derivatives, the inhibition assays were performed exclusively with HIF-1α_788–822_^37^ owing to its reduced reactivity, facilitating the experimental set-up. Half maximum inhibitory concentrations (IC_50_-values) of the 2OG derivatives were determined (comprehensive analysis in Supplementary Table 1), using NOFD^52^ as a positive inhibition control. The IC_50_ for NOFD (~0.2 μM; Table 3, entry 1) is substantially lower in the SPE-MS assay compared to a previously employed derivatization assay (${K}_{i}^{{{{{{\rm{app}}}}}}}$ ~ 93 μM)^52^, highlighting the better sensitivity of this direct assay (0.15 μM FIH was used in the SPE-MS assay and 4.0 μM FIH in the derivatization assay).

**Table 3:** Synthetic 2OG derivatives inhibit FIH.

^a^The synthetic C3/C4-substituted 2OG derivatives were prepared via a reported procedure (Supplementary Fig. 1)^21^; all chiral 2OG derivatives except for NOFD are racemates.

^b^Mean of two independent runs (*n* = 2; mean ± SD). FIH inhibition assays were performed as described in the Methods section using 0.15 μM FIH and 5.0 μM HIF-1α_788–822_^37^. FIH inhibition assays were of good quality which high S/N ratios and Z’-factors^83^ (>0.5 for each plate) indicate (Supplementary Fig. 26); Hill coefficients^93^ of the inhibition curves were in the range of the expected value −1 for 2OG derivatives inhibiting FIH.

The increase in length of the 2OG C3/C4-substituent from methyl via ethyl to propyl appears to correlate with enhanced FIH inhibition (Table 3). Thus, although the C3/C4 methyl-substituted 2OG derivatives **1** and **12** do not inhibit FIH (Table 3, entries 2 and 7), 3-propyl-2OG (**3**) inhibits FIH efficiently (IC_50_ ~2.7 μM; Table 3, entry 4). The potencies of the synthetic 2OG derivatives as a function of substituent length appear to inversely correlate with their cosubstrate efficiency (Tables 1 and 3). For example, high levels of FIH cosubstrate activity, but not FIH inhibition, were observed for **1** and **12** (Table 1, entries 2 and 13), whereas the C4 ethyl and propyl-substituted 2OG derivatives **14** and **15** are more efficient FIH cosubstrates, but less efficient FIH inhibitors compared to the corresponding C3 regioisomers **2** and **3** (Tables 1 and 3).

C4-Benzyl-2OG (**19**) inhibits FIH efficiently in the SPE-MS assay (IC_50_ ~ 3.6 μM; Table 3, entry 12), whereas only weak inhibition has been reported when employing a 2-oxo acid derivatization assay^52^. Notably, **19** inhibits FIH more than one order of magnitude less efficiently than the structurally related, but conformationally more rigid, NOFD (note that NOFD was used in the enantiopure form, while **19** was used as a racemic mixture). However, the potency of **19** was increased when replacing its C4 benzyl- with a 3-phenylpropyl-substituent (IC_50_ ~0.9 μM; Table 3, entry 11). The C4 benzyl- and C4 3-phenylpropyl-substituted 2OG derivatives **19** and **18** are two- and sixfold, respectively, more efficient FIH inhibitors than their corresponding C3 regioisomers (Table 3, entries 5, 10, 11, and Supplementary Table 1). However, the potency of C3 benzyl-derivative **6** could be substantially enhanced when introducing a sterically bulkier CH_2_(9,9-dimethyl-9*H*-fluoren-2-yl-substituent at C3 (IC_50_ ~ 1.9 μM; Table 3, entry 6).

In general, the 2OG derivatives inhibit FIH less efficiently than AspH, which might, at least to some extent, reflect the higher 2OG concentration used in the FIH inhibition assays (10 μM 2OG in the FIH assays, 3 μM 2OG in the AspH assays). Thus, the C4 ethyl- and propyl-substituted 2OG derivatives **14** and **15** are highly efficient AspH inhibitors, consistent with their reported lack of AspH cosubstrate activity^21^, whereas the same compounds are more than an order of magnitude less efficient in inhibiting FIH (Table 3, entries 9 and 10). Inhibition selectivity is even higher for 4,4-dimethyl-2OG (**13**), which efficiently inhibits AspH (IC_50_ ~ 0.4 μM^21^) but not at all FIH (Table 3, entry 8). This result highlights the importance of apparently minor structural modifications on inhibitor selectivity as 4-methyl-2OG (**12**) neither inhibits AspH nor FIH (Table 3, entry 7) and also reveals the potential of 2OG-competitive 2-oxo acids for selective 2OG oxygenase inhibition (the mode of AspH inhibition by **13** has previously been shown to be 2OG-competitive^21^).

Interestingly, varying the substitution pattern of the 2OG derivatives can result in a reversal of inhibition selectivity. For example, 3-ethyl 2OG (**2**) inhibits AspH more efficiently than FIH (IC_50_ ~ 1.2 μM for AspH and ~4.6 μM for FIH; AspH:FIH inhibitor selectivity ~4:1; Table 3, entry 3), however, substituting its C3 ethyl with a propyl group reverses inhibition selectivity; the corresponding 2OG derivative **3** inhibits FIH more efficiently than AspH (IC_50_ ~ 5.7 μM for AspH and ~2.7 μM for FIH; AspH:FIH inhibitor selectivity ~1:2; Table 3, entry 4). Similarly, the substitution of a 2OG CH_2_-unit by an NH-unit can result in a reversal of inhibition: While 4-benzyl-2OG (**19**) is more efficient in inhibiting AspH compared to FIH (AspH:FIH inhibitor selectivity: ~7:1; Table 3, entry 12), its NH-containing derivative NOFD is substantially more efficient in inhibiting FIH rather than AspH (AspH:FIH inhibitor selectivity: ~1:50; Table 3, entry 1).

2OG derivative **28** neither inhibits AspH nor FIH catalysis (Table 3, entry 13) and also derivatives of **28** bearing substituents at the aromatic core do not inhibit the two oxygenases (Supplementary Table 1). In principle, selective 2OG oxygenase inhibition by 2OG derivatives bearing an aromatic or heteroaromatic core appears to be feasible, as revealed by the inhibition profile of 2OG derivative **35**, which bears a thiophene moiety and selectively inhibits AspH over FIH (Table 3, entry 14).

**Fig. 4:**
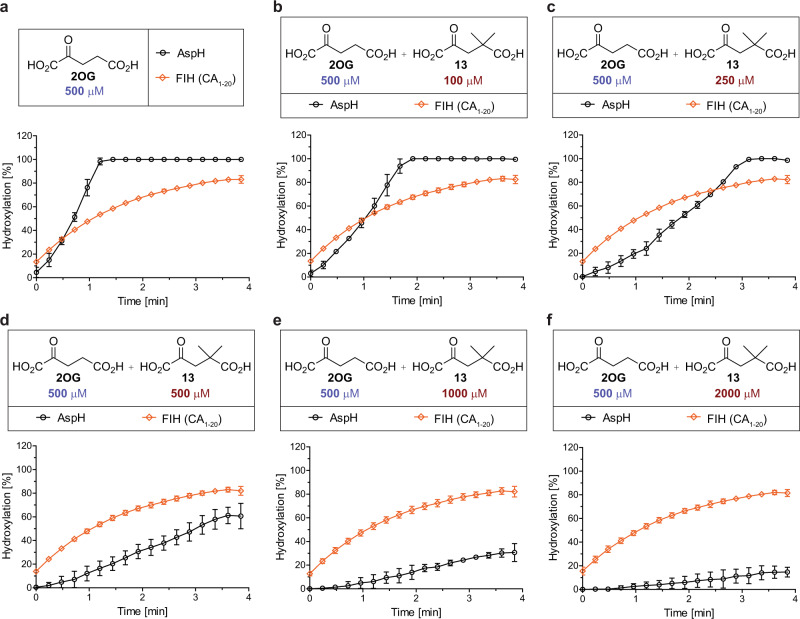
4,4-Dimethyl-2OG selectively inhibits AspH in the presence of FIH at physiological 2OG concentrations. 2OG oxygenase assays were performed as described in the Methods section using 0.15 μM of each 2OG oxygenase (diamond: FIH, circle: AspH) and 5.0 μM of each substrate (orange: CA_1–20_^47^; black: hFX-EGFD1_86–124_-4Ser^31^, AspH substrate) in the presence of 100 μM LAA, 10 μM FAS, and the indicated concentrations of cosubstrates in buffer (50 mM Tris, 50 mM NaCl, pH 7.5). Mean of three independent runs (*n* = 3; mean ± SD). Measurement times were normalized to the first sample injection analyzed after the addition of 2OG oxygenase to the Substrate Mixture (*t* = 0 min), by which time low levels of hydroxylation were manifest. Titration of 4,4-dimethyl-2OG (**13**) to a reaction mixture containing 500 μM 2OG: **a** 0 μM **13**; **b** 100 μM **13**; **c** 250 μM **13**; **d** 500 μM **13**; **e** 1000 μM **13**; **f** 2000 μM **13**. Source data are provided as a Source Data file.

The inhibition results suggest that the ratio of FIH to AspH activity may be perturbed by using an AspH-selective inhibitor in the presence of the natural cosubstrate 2OG, a strategy that is complementary to the one previously investigated relying on the use of an FIH-selective cosubstrate (i.e., **14**; Fig. 2f). AspH was most selectively inhibited by 4,4-dimethyl-2OG (**13**), which binds to AspH efficiently and is slowly converted into 2,2-dimethyl succinate (Table 3, entry 8)^21^; therefore, FIH- and AspH-catalyzed substrate hydroxylations were simultaneously assessed in a single reaction vessel using SPE-MS in the presence of a physiological relevant 2OG concentration (0.5 mM) and varied concentrations of **13** (Fig. 4). Increasing the concentration of **13** from 0 to 2.0 mM reveals selective AspH inhibition in a dose-dependent manner, with no effect on FIH activity being observed. In the absence of the inhibitor **13**, AspH catalysis is substantially faster than FIH catalysis, with substrate hydroxylation reaching apparent completion after ~1 min; ~80% FIH-catalyzed substrate turnover was only observed after 4 min (Fig. 4a). Using equimolar amounts of 2OG and **13** (0.5 mM each), ~80% conversion is observed for FIH after 4 min while only ~60% conversion is observed for AspH (Fig. 4d). In the presence of a fourfold excess of **13** with respect to 2OG, <20% of AspH-catalyzed substrate hydroxylation was observed after 4 min (Fig. 4f). A comparable result was obtained in a titration experiment with 2OG and 4-ethyl-2OG (**14**), which is an alternative FIH cosubstrate and an AspH inhibitor (Fig. 2f and Table 3, entry 9)^21^. The titration revealed that a tenfold excess of **14** over 2OG substantially slows down AspH catalysis while showing only a marginal effect on FIH catalysis (Supplementary Fig. 27). The results reveal the potential of 2-oxo acids for selective 2OG oxygenase inhibition at physiologically relevant 2OG concentrations.

### Mechanism of FIH inhibition

**Fig. 5:**
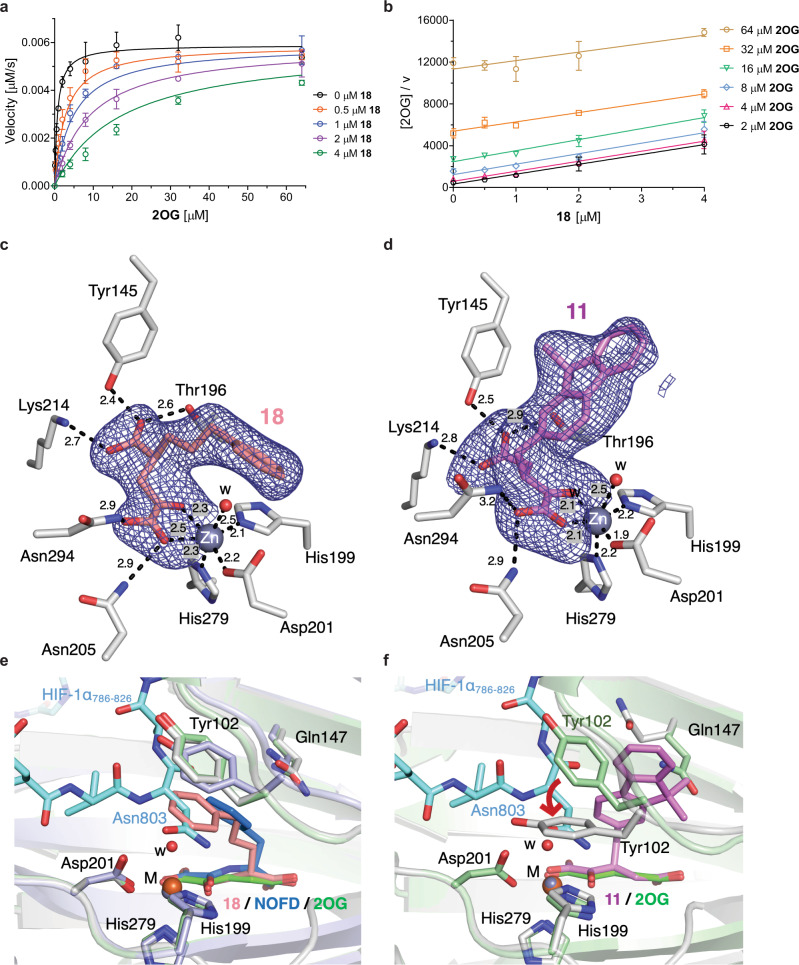
The 2OG derivatives 11 and 18 inhibit FIH by different modes. **a** Effect of variable **18** concentrations on the Michaelis–Menten kinetics. Mean of three independent runs (*n* = 3; mean ± SD); **b** Cornish-Bowden plot^63^ for **18**; **c** and **d** representative OMIT electron density map (mF_o_-DF_c_) contoured to **c** 5.0σ around (*S*)-**18** of the FIH:**18** structure (FIH: gray; Zn ion: lavender blue; carbon-backbone of **18**: salmon; w: water) and **d** 3.0σ around (*S*)-**11** of the FIH:**11** structure (FIH: gray; Zn ion: lavender blue; carbon-backbone of **11**: violet; w: water), respectively; **e** superimposition of a view from the FIH:**11** structure with views from the reported FIH:NOFD (FIH: light blue; Fe ion: orange; carbon-backbone of NOFD: deep blue; PDB ID: 1YCI^52^) and FIH:2OG:HIF-1α_786–826_ (FIH: pale green; Fe ion: orange; carbon-backbone of 2OG: green; carbon-backbone of HIF-1α_786–826_: cyan; PDB ID: 1H2L^61^) structures reveals a similar mode of inhibition for NOFD and **18**; **f** superimposition of a view from the FIH:**11** structure with one from the reported FIH:2OG:HIF-1α_786–826_ structure (FIH: pale green; Fe ion: orange; carbon-backbone of 2OG: green; carbon-backbone of HIF-1α_786–826_: cyan; PDB ID: 1H2L^61^) reveals that the binding of **11** to FIH triggers conformational changes in the Tyr102_FIH_ sidechain. Source data are provided as a Source Data file, information on crystallographic data is provided in the Data availability section.

To investigate the mechanism by which the synthetic 2OG derivatives inhibit FIH, the concentration-dependent effect of the potent FIH inhibitor **18** on the 2OG ${K}_{m}^{{{{{{\rm{app}}}}}}}$-value was investigated using HIF-1α_788–822_ (Supplementary Fig. 28). The 2OG ${K}_{m}^{{{{{{\rm{app}}}}}}}$-value decreases in a linear fashion with ascending inhibitor concentrations, while the ${v}_{{{{{{\rm{max }}}}}}}^{{{{{{\rm{app}}}}}}}$-values converge, indicating a linear 2OG-competitive inhibition mechanism (Fig. 5a). Using non-linear regression, the ${K}_{i}$-value of **18** was determined to be ~0.2 μM. Analysis of a Cornish-Bowden plot^63^ further supports a 2OG-competitive inhibition mode, i.e., parallel lines for different 2OG concentrations are obtained when plotting [2OG]/*v* against [**18**] and their intercept with the *y* axis increases on increasing the 2OG concentration (Fig. 5b).

FIH was crystallized in the presence of Zn(II) and the 2OG derivatives **11** or **18** to investigate their binding modes. FIH crystallized in the *P*4_1_2_1_2 space group (FIH:**11**, 1.99 Å resolution; FIH:**18**, 1.90 Å resolution; Supplementary Figs. 29 and 31). Clear electron density corresponding to **18** or **11**, respectively, was observed in the active site (Fig. 5c and d); **11** and **18** bind FIH in a similar manner as 2OG (Fig. 5e). The analysis of the electron density maps for the 2OG derivatives indicates that only (*S*)-**11** and (*S*)-**18** bind FIH, even though both were present as racemic mixtures (Supplementary Figs. 30 and 32).

The conformation of (*S*)-**18** in the FIH:**18** structure is reminiscent of the NOFD conformation in the reported FIH:NOFD^52^ structure, indicating that these molecules inhibit FIH by a similar mechanism: their bulky phenyl substituents extent into the FIH substrate-binding pocket and appear to shield the active site metal from the substrate and/or O_2_ (Fig. 5e). By contrast, the rigid and relatively bulky fluorenyl substituent of (*S*)-**11** orients in a different manner than the substituents of (*S*)-**18** and NOFD, triggering a conformational change in the Tyr102_FIH_ sidechain. In the FIH:**11** structure, the Tyr102_FIH_ sidechain faces into the substrate-binding position, likely to avoid a steric clash with the fluorenyl substituent of (*S*)-**11**, and thus blocks the substrate from binding to the FIH active site (red arrow in Fig. 5f). Movement of the Tyr102_FIH_ sidechain has been previously observed in crystal structures of FIH complexed with different substrates (Supplementary Fig. 33)^43^. In agreement with the proposal that the coordination of (*S*)-**11** and (*S*)-**18** to the FIH active site impairs substrate binding, FIH crystals with both inhibitor and substrate-bound could not be obtained. Even though inhibitors **11** and **18** display no substantial selectivity between AspH and FIH, the crystallographic analyses may help in the design of selective FIH inhibitors.

## Discussion

**Fig. 6:**
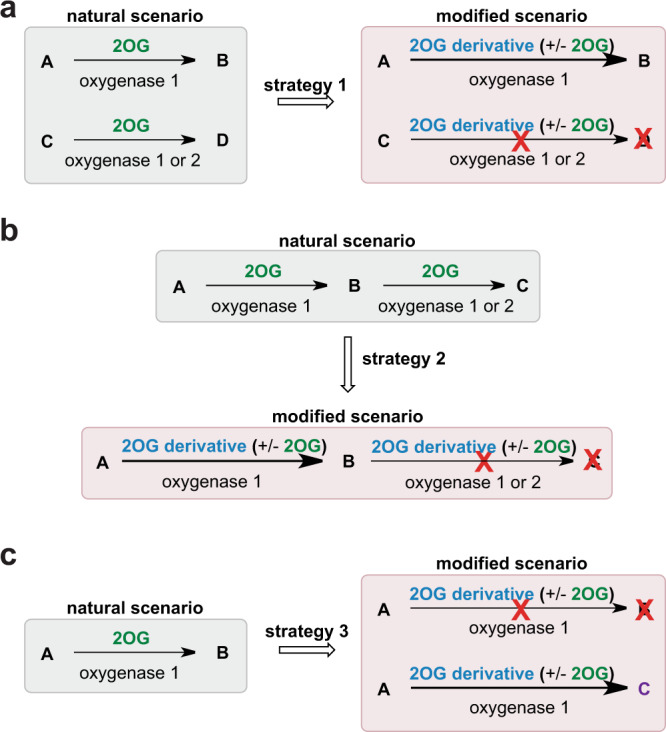
2OG derivatives may be used to selectively control reaction outcomes of 2OG oxygenase catalysis. Each enzyme reaction involves two-electron substrate oxidation coupled to the conversion of 2OG/O_2_ to succinate/CO_2_. The substitution of 2OG by a 2OG derivative or the addition of an inhibitory 2OG derivative has the potential to alter natural reaction outcomes of one or multiple 2OG oxygenase reactions. For example, it may enable the selective enhancement/inhibition of catalysis by one 2OG oxygenase in the presence of another/others (**a** strategy 1), the perturbation of sequential 2OG oxygenase reactions (**b** strategy 2), or the formation of different reaction products in a 2-oxo acid cosubstrate-dependent manner (**c** strategy 3).

Different strategies can be envisaged in which 2OG derivatives might be used to alter the normal reaction outcomes of one or more 2OG oxygenases (Fig. 6). In one case, a 2OG derivative might selectively enhance and/or inhibit the activity of one 2OG oxygenase in the presence of others (strategy 1, Fig. 6a). The application of reaction-selective 2OG oxygenase cosubstrates could also enable control of reaction outcomes for sequential reactions catalyzed by a 2OG oxygenase (strategy 2, Fig. 6b). A 2OG oxygenase may also be envisaged to convert its substrate to a different product with a 2OG derivative compared to 2OG (strategy 3, Fig. 6c). In the current work, we describe proof-of-principle studies that 2OG derivatives can be used to enable strategy 1 (Fig. 6a), i.e., to selectively modulate the activity of one or both of two human 2OG oxygenases, which both catalyze the hydroxylation of Asn- and Asp-residues, i.e., FIH and AspH.

Our results of screens of 2OG derivatives with FIH and AspH led to the identification of two complementary approaches to selectively modulate the activities of FIH and AspH (strategy 1, Fig. 6a). FIH activity can be selectively enhanced in the presence of AspH using either an FIH-selective cosubstrate (i.e., **14**; Fig. 2f) in the absence of 2OG, or an AspH-selective inhibitor in the presence of a physiologically relevant concentration of 2OG (i.e., **13**; Fig. 4). AspH activity can be selectively enhanced in the presence of FIH using a partially AspH-selective cosubstrate (i.e., **28**; Supplementary Fig. 8) in the absence of 2OG. High levels of cosubstrate/inhibitor selectivity were observed in all cases, revealing the potential of 2-oxo acids for the selective enhancement and/or inhibition of 2OG oxygenase catalysis. This potential is supported by the reported lack of cosubstrate/inhibition activity of some of the tested C4-alkyl-substituted 2OG derivatives with wild-type KDM4A, which is a 2OG-dependent JmjC KDM^19^.

The application of MS-based assays to simultaneously assess FIH and AspH activity in the same reaction vessel complements kinetic studies with isolated 2OG oxygenases and enables the direct comparison of 2OG oxygenase activities. Importantly, the simultaneous turnover assays show that natural products can have a profound impact on the reactivity profiles of isolated 2OG oxygenases. For example, 3-methyl-2OG (**1**), which is an ingredient of honey^48^, promotes AspH catalysis relative to FIH catalysis, whereas its regioisomer, 4-methyl-2OG (**12**), which is present in plants^49,50^ and in wine^51^, promotes FIH catalysis relative to AspH catalysis in the absence of 2OG (Fig. 2; strategy 1, Fig. 6a). Considering that cancer-associated mutations of the human isocitrate-dehydrogenase complex result in a 2OG deficient phenotype which, along with elevated 2-hydroxyglutarate levels, can correlate with decreased 2OG oxygenase activity^64,65^, the result that naturally occurring 2OG derivatives can enable 2OG oxygenase activity could be of in vivo relevance and medicinal value, e.g., to treat side effects arising from reduced 2OG oxygenase activity.

Some proteins, such as human Notch, are substrates of both AspH and FIH: AspH catalyzes the hydroxylation of extracellular Notch EGFDs^66^ and FIH catalyzes the hydroxylation of ankyrin repeats in the Notch intracellular domain^39^. Nature likely controls the FIH and AspH-catalyzed post-translational Notch modifications by spatial separation of the enzymes (FIH is predominantly present in the cytosol^67^, whereas the oxygenase domain of AspH faces into the ER^68^). The use of a selective cosubstrate may enable the selective enhancement of AspH or FIH catalysis acting on the same substrate, thus complementing natural control by spatial separation. Although there is likely scope for improvement of the synthetic 2OG derivatives reported by us, our studies provide proof-of-principle for this concept, i.e., 4-carboxyphenylglyoxylic acid (**28**) is a substantially more efficient AspH than FIH cosubstrate (Supplementary Fig. 8), whereas 4-ethyl-2OG (**14**) is an FIH cosubstrate but an AspH inhibitor (Fig. 2f).

The comparison of FIH:2OG derivative structures with reported AspH:2OG derivative structures reveals that the unusual Fe(II)-binding geometry of AspH, which has only two Fe(II) binding ligands compared to the typical triad present in other 2OG hydroxylases (including in FIH and KDM4A)^21^, does not appear to underpin the observed differences in cosubstrate selectivity patterns. In near all the FIH:2OG derivative and AspH:2OG derivative structures, the 2OG derivatives occupy the 2OG-binding site and display similar conformations as 2OG (Fig. 3); a notable exception is the structurally distinct 2OG derivative **22**, whose conformation differs owing to its cyclic structure (Fig. 3e). Together with the reported lack of cosubstrate activity of certain C4-substituted 2OG derivatives with KDM4A^19^, these observations suggest that the interactions of the C3/C4-substituents of the 2OG derivatives with sidechains of (hydrophobic) residues flanking the 2OG-binding site, which form hydrophobic regions, determine the efficiency of cosubstrate binding and coproduct release (Fig. 3f). In general, the FIH ${k}_{{{{{{\rm{cat}}}}}}}$/${K}_{m}$-values for the 2OG derivatives appear to decrease upon increasing the length of the 2OG C4 substituent from hydrogen to methyl and ethyl, independent from the nature of the substrate (Table 2). As the ${K}_{m}^{{{{{{\rm{app}}}}}}}$-values remain in the same range within experimental error (0.5~3.1 μM, Table 2), this observation indicates that the 2OG derivatives bind FIH with similar affinities, but likely undergo subsequent oxidative decarboxylation reactions less efficiently than 2OG, resulting in apparent inhibition. Although they employ the same general strategies for 2OG binding^69^, the 2OG-binding sites of different 2OG oxygenases vary substantially and thus may be particularly suited for the binding of specific 2OG derivatives resulting either in selective inhibition or activation as evidenced by our work.

The results presented here highlight the potential of 2-oxo acids, including 2OG derivatives and natural products, to inhibit 2OG oxygenases and related enzymes. The potential of 2-oxo acids for the inhibition of 2OG oxygenases is supported by the (partially) selective FIH inhibitor NOFD, a derivative of the broader spectrum 2OG oxygenase inhibitor NOG^52^. The inhibition selectivity of NOG derivatives has also been studied for other subclasses of 2OG oxygenases including the JmjC KDMs and TET enzymes^70–73^. The observation that dimethyl *N*-oxalylglycine (DMOG), a cell-wall penetrable NOG precursor, inhibits 2OG oxygenases in vivo and in cellular studies, supports the potential utility of corresponding 2OG derivatives for cellular functional assignment studies, regardless of the high 2OG concentrations in cells (~1 mM 2OG^57,58^). The potential of 2-oxo acids to inhibit 2OG oxygenases is shown by the results for 4,4-dimethyl-2OG (**13**), which efficiently and selectively inhibits AspH (IC_50_ ~ 0.4 μM^21^), even in the presence of physiologically relevant 2OG concentrations, i.e., 0.5 mM 2OG (Fig. 4).

The involvement of 2-oxo acids in multiple metabolic pathways may be considered to limit the utility of 2OG derivatives for their development as human therapeutics. Indeed, the presence of 2OG derivatives with substituents at the C3/C4 position may modulate the activities of 2OG-dependent enzymes other than 2OG oxygenases. For example, it has been reported that 4,4-dimethyl-2OG (**13**) is a substantially less-efficient substrate of glutamic oxaloacetic aminotransferase compared with the natural substrate 2OG or 4-methyl-2OG (**12**), suggesting that **13** might not interfere with the catalytic activities of human aminotransferases^74^. However, from a safety perspective, it is notable that some of the 2OG derivatives described here are present in human nutrition^48–51^ and cells appear to tolerate reasonably high levels of DMOG with no obvious toxicity^75^. Further, considering the pleiotropic effects that NOG and its derivatives exhibit in cellular studies, application of related 2-oxo acid derivatives to cells may trigger phenotypes useful in identifying previously overlooked 2OG oxygenase/2OG-utilizing enzyme functions. FIH^76^ and AspH^22–26^ are proposed medicinal chemistry targets and the use of (partially) selective 2OG derivatives, such as **13** or corresponding esters in cellular studies may help to improve the currently limited understanding on their biochemical roles and complement similar approaches using heterocyclic small-molecules^24,76–81^.

In summary, the combined results with FIH and AspH, 2OG oxygenases that both catalyze Asp- and Asn-residue hydroxylations, demonstrate the potential of 2-oxo acid derivatives to selectively enhance or inhibit one of the enzyme reactions in the presence of the other, in some cases in the presence of 2OG. From a set of 35 2OG derivatives, we identified 10 as enhancing and 17 as inhibiting FIH activity. Although further mechanistic studies are required to analyze the details of why some 2-oxo acid derivatives inhibit and others enable 2OG oxygenase catalysis, our structural studies provide insight into the active site regions into which the C3/C4 2OG substituents bind; this information should be useful in the identification of 2-oxo acid derivatives, including natural products present in food, that enable selective modulation of 2OG oxygenase reaction outcomes in vivo. Analogous strategies described here for modulation of 2OG oxygenase activity by cosubstrate derivatives should be applicable to other enzyme families employing modified cosubstrates.

## Methods

### Protein production and purification

Wildtype N-terminally His_6_-tagged human FIH was produced and purified by an adaption of a literature procedure^82^. In brief, DNA encoding for wild-type human FIH with a N-terminal His_6_-tag was cloned into a pNIC28-Bsa4 plasmid. After transformation of the plasmid into *E. coli* BL21 (DE3) cells, expression was performed at 37 °C in LB media supplemented with 37.5 µg/mL kanamycin. FIH-production was induced at an OD_600_ of ~0.6 at 20 °C by adding isopropyl β-d-thiogalactopyranoside (final concentration of 0.5 mM). Cultures were grown overnight. Cells were spun at 5000 rpm for 10 mins and the resulting cell pellets were stored at −80 °C.

The cell pellets were suspended in buffer (50 mM HEPES, pH 7.4, 500 mM NaCl, 20 mM imidazole, 0.5 mM TCEP) and lysed by high-pressure homogenization (three passages). Following cell lysis, the insoluble cell debris was removed by centrifugation (36,000 × *g*, 1 h, 4 °C). His_6_-tagged FIH was purified by sequential Ni(II)-affinity chromatography (using 50 mM HEPES, pH 7.4, 1 M NaCl, 0.5 mM TCEP with increasing imidazole concentrations: 20–300 mM) and size-exclusion chromatography (HiLoad 16/600 Superdex 75 pg column; buffer: 20 mM HEPES, pH 7.4, 500 mM NaCl, 5% glycerol, 0.5 mM TCEP) using an ÄKTAxpress machine. Fractions containing FIH were pooled and concentrated; FIH was >95% pure by SDS-PAGE and MS analysis. Purified FIH was stored at a concentration of 42 μM (20 mM HEPES, pH 7.4, 500 mM NaCl, 5% glycerol, 0.5 mM TCEP) at −78 °C, fresh aliquots were used for all FIH assays (cleavage of the His_6_-tag did not show beneficial effects on FIH activity).

A truncated construct of wild-type N-terminally His_6_-tagged human AspH, comprising the catalytic oxygenase domain and the tetratricopeptide repeat domain (His_6_-AspH_315-758_), was produced and purified as previously reported^27,31^.

### Substrate peptides

FIH substrates were based on the sequence of the two reported human FIH substrate proteins HIF-1α^34,36^ (HIF-1α C-terminal transactivation domain fragment amino acids 788–822, HIF-1α_788–822_: DESGLPQLTSYDCEVNAPIQGSRNLLQGEELLRAL; FIH catalyzes hydroxylation of Asn803^34,37^) and tankyrase-2^62^ (tankyrase-2 amino acids 691–710, TNKS2_691–710_: NLEVAEYLLQHGADVNAQDK; FIH catalyzes hydroxylation of Asn706^62^) and of a reported synthetic consensus ankyrin repeat^46^ (CA_1–20_:^47^ HLEVVKLLLEAGADVNAQDK; FIH catalyzes hydroxylation of Asn16^47^). The AspH substrate peptide, i.e., hFX-EGFD1_86–124_-4Ser^27,31^, was based on the sequence of the EGFD1 (amino acids 86–124) of the reported AspH substrate human coagulation factor X (hFX), four hFX cysteine residues (Cys90_hFX_, Cys95_hFX_, Cys112_hFX_, Cys121_hFX_) have been substituted for serine residues to avoid disulfide scrambling. Peptides were synthesized by solid-phase peptide synthesis and purified by GL Biochem (Shanghai) Ltd (Shanghai, China); all peptides were prepared with C-terminal amides.

### FIH inhibition assays

FIH inhibition assays were performed as described^44^, using cosubstrate/cofactor stock solutions (l-ascorbic acid, LAA: 50 mM in MQ-grade water; 2-oxoglutarate, 2OG: 10 mM in MQ-grade water; ammonium iron(II) sulfate hexahydrate, FAS, (NH_4_)_2_Fe(SO_4_)_2_·6H_2_O: 400 mM in 20 mM HCl diluted to 1 mM in MQ-grade water), which were freshly prepared from commercially-sourced solids (Sigma Aldrich).

Dimethyl sulfoxide (DMSO) solutions of the 2OG derivatives were dry dispensed across 384-well polypropylene assay plates (Greiner) in an approximately threefold and 11-point dilution series (100 μM top concentration; the final DMSO assay concentration was kept constant at 0.5%_v/v_) using an ECHO 550 acoustic dispenser (Labcyte). DMSO and NOFD^52^ were used as negative and positive inhibition controls, respectively. Each reaction was performed in technical duplicates in adjacent wells of the assay plates; additionally, assays were performed in two independent duplicates on different days using different inhibitor solutions.

The Enzyme Mixture (25 μL/well), containing 0.3 μM His_6_-FIH in buffer (50 mM Tris, 50 mM NaCl, pH 7.5), was dispensed across the inhibitor-containing 384-well assay plates with a multidrop dispenser (ThermoFischer Scientific) at 20 °C under an ambient atmosphere. The plates were subsequently centrifuged (1000 rpm, 15 s) and incubated for 15 min at 20 °C. The Substrate Mixture (25 μL/well), containing 10.0 μM HIF-1α_788–822_^37^ substrate, 200 μM LAA, 20.0 μM 2OG, and 20.0 μM FAS in buffer (50 mM Tris, 50 mM NaCl, pH 7.5), was added using the multidrop dispenser. The plates were centrifuged (1000 rpm, 15 s) and after incubating for 15 min, the enzyme reaction was stopped by the addition of 10%_v/v_ aqueous formic acid (5 μL/well). The plates were then centrifuged (1000 rpm, 30 s) and analyzed by MS.

MS-analyses were performed using a RapidFire RF 365 high-throughput sampling robot (Agilent) attached to an iFunnel Agilent 6550 accurate-mass quadrupole time-of-flight mass spectrometer operated in the positive ionization mode. Assay samples were aspirated under vacuum for 0.6 s and loaded onto a C4 SPE cartridge. After loading, the C4 SPE cartridge was washed with 0.1%_v/v_ aqueous formic acid to remove non-volatile buffer salts (5.5 s, 1.5 mL/min). The peptide was eluted from the SPE cartridge with 0.1%_v/v_ aqueous formic acid in 85/15 _v/v_ acetonitrile/water into the mass spectrometer (5.5 s, 1.25 mL/min) and the SPE cartridge re-equilibrated with 0.1%_v/v_ aqueous formic acid (0.5 s, 1.25 mL/min). The mass spectrometer was operated using the MassHunter Workstation B.08.00 software (Agilent), the mass spectrometer parameters were: capillary voltage (4000 V), nozzle voltage (1000 V), fragmentor voltage (365 V), gas temperature (280 °C), gas flow (13 L/min), sheath gas temperature (350 °C), sheath gas flow (12 L/min). The m/z + 2 (for CA_1–20_) or +3 (for HIF-1α_788–822_) charge states of the peptide (substrate) and the hydroxylated peptide (product) were used to extract ion chromatogram data, peak areas were integrated using RapidFire Integrator 4.3.0 (Agilent). Data were exported into Microsoft Excel and used to calculate the % conversion of the hydroxylation reaction using the equation: % conversion = 100 × (integral product peptide)/(integral substrate peptide+integral product peptide). Normalized dose–response curves (NOFD and DMSO controls) were obtained from the raw data by non-linear regression (GraphPad Prism 5) and used to determine IC_50_ values. The standard deviation (SD) of two independent IC_50_ determinations (*n* = 2) was calculated using GraphPad Prism 5. Z′-factors were calculated according to the cited literature using Microsoft Excel (Supplementary Fig. 26)^83^.

### Determination of kinetic parameters

Maximum velocities (${v}_{{{{{{\rm{max }}}}}}}^{{{{{{\rm{app}}}}}}}$) and Michaelis constants (${K}_{m}^{{{{{{\rm{app}}}}}}}$) of FIH were determined in independent triplicates for 2OG and the synthetic 2OG derivatives by SPE-MS monitoring HIF-1α_788–822_^37^ or CA_1–20_^47^ turnover. His_6_-FIH (1.8 μL, 42 μM) was added at 20 °C to a substrate mixture containing 5.0 μM substrate, 100 μM LAA, 20 μM FAS, and 2OG/2OG derivative in 0.5 mL buffer (50 mM Tris, 50 mM NaCl, pH 7.5). Final 2OG/2OG derivative concentrations are given in Supplementary Figs. 5 and 6. Reactions were monitored with a rate of ~1 sample/40 s using the same SPE-MS configuration as described above. Data were analyzed as described above and the slopes of the initial reaction rates (Supplementary Figs. 5 and 6) fitted to a Michaelis–Menten plot using non-linear regression (GraphPad Prism 5). The total concentration of active FIH was determined by an active site titration (Supplementary Fig. 7) and used to calculate turnover numbers (${k}_{{{{{{\rm{cat}}}}}}}^{{{{{{\rm{app}}}}}}}$) and specificity constants (${k}_{{{{{{\rm{cat}}}}}}}$/${K}_{m}$) for 2OG and the 2OG derivatives.

### Competition assays

To a Substrate Mixture containing 5.0 μM of each of the requisite substrates (for FIH: HIF-1α_788–822_^37^ and/or CA_1–20_^47^; for AspH: hFX-EGFD1_86–124_-4Ser^27,31^), 100 μM LAA, 10 μM FAS, and 20 μM 2OG/2OG derivative in 1.0 mL buffer (50 mM Tris, 50 mM NaCl, pH 7.5) was added 0.15 μM of His_6_-FIH and His_6_-AspH_315-758_ (when appropriate). Peptide turnover was monitored by SPE-MS using the same instrument configuration (with the exception that the peptides were eluted from the SPE cartridge with 0.1%_v/v_ aqueous formic acid in 80/20 _v/v_ acetonitrile/water) as described above.

### Crystallography

Crystallization experiments were performed in 96-well, three-subwell, low profile Intelliplates (Art Robbins Instruments) using a Phoenix RE liquid dispensing robot (Art Robbins Instruments) with 1.6 M ammonium sulfate, 6%_w/v_ PEG400, and 0.1 M HEPES buffer (pH 7.5) as the mother liquid. N-Terminally His_6_-tagged FIH (20 mg/mL) was mixed with ZnCl_2_ (1 mM), 2OG derivative (2 mM), and, when appropriate, a synthetic consensus ankyrin repeat peptide (5 mM; CA_1–20_^47^) or a tankyrase-2 fragment peptide^62^ (5 mM; TNKS2_691–710_) as FIH substrate. Crystals were grown using the vapor diffusion method at 20 °C in 200 nL of 300 nL sitting drops with 2:1, 1:1, or 1:2 sample:well solution ratios; precipitants are listed in Supplementary Tables 2 and 3. Crystals were cryo-protected using mother liquor supplemented with 25%_v/v_ glycerol before cryo-cooling in liquid N_2_. Data were collected at 100 K using synchrotron radiation at diamond light source beamlines I03 and I04. Data were indexed, integrated, and scaled using the autoPROC^84^, STARANISO^85^, or Xia2^86^ strategy of the beamline auto-processing pipeline (Supplementary Tables 2 and 3).

The FIH crystal structures were determined by MR using the AutoMR (PHASER^87^) subroutine in PHENIX (version 1.18.2)^88^. The search model used for MR was based on PDB ID 1H2K^61^ for FIH crystal structures. The structural model was improved by iterative cycles of manual re-building in Coot (version 0.8.6.1)^89^ and crystallographic refinement in phenix.refine^90^ (refinement details are summarized in Supplementary Tables 2 and 3). The crystal structure data for FIH complexed to Zn, 2OG derivative, and, in some cases, substrate (i.e. CA_1–20_ or TANK2_691–710_) have been deposited in the protein data bank (see Data availability section). PyMOL (version 4.6)^91^ was used for the generation of graphical representations; polder omit maps were calculated using Polder Maps^92^ in PHENIX (version 1.18.2)^88^.

### Reporting summary

Further information on research design is available in the Nature Research Reporting Summary linked to this article.

## Supplementary information

Supplementary Information

Reporting Summary

## Data Availability

The crystal structure data for FIH complexed to Zn, 2OG derivative (3-methyl-2OG, **1**; 3-propyl-2OG, **3**; 3-(9,9-dimethyl-9*H*-fluoren-2-yl)methyl-2OG, **11**; 4-ethyl-2OG, **14**; 4-propyl-2OG, **15**; 4-(3-phenylpropyl)-2OG, **18**; 3-(carboxycarbonyl)cyclopentane-1-carboxylic acid, **22**), and, in some cases, substrate (i.e. CA_1–20_ or TANK2_691–710_) have been deposited in the protein data bank under PDB accession codes: 7A1L (FIH:**1**), 7A1M (FIH:**3**), 7A1J (FIH:**18**), 7A1K (FIH:**11**), 7A1N (FIH:**1**:CA_1–20_), 7A1O (FIH:**14**:CA_1–20_), 7A1P (FIH:**15**:CA_1–20_), 7A1Q (FIH:**22**:CA_1–20_), and 7A1S (FIH:**1**:TANK2_691–710_). In addition, data of the following reported crystal structure have been used: 1H2L^61^, 1H2K^61^, 1YCI^52^, and 6YYX^21^. Source data are provided with this paper.

## Source data

Source Data File
