# Supplementary material for: 2-Oxoglutarate derivatives can selectively enhance or inhibit the activity of human oxygenases
Source: Nat Commun. 2021 Nov 10;12:6478. doi: 10.1038/s41467-021-26673-2 (PMC8580996; doi:10.1038/s41467-021-26673-2)
Supplement: Supplementary file 1 — Supplementary Information [file 41467_2021_26673_MOESM1_ESM.pdf]

Supplementary Information

2-Oxoglutarate derivatives can selectively enhance or inhibit the activity of human oxygenases

Yu Nakashima[1,2,#], Lennart Brewitz[1,#], Anthony Tumber[1], Eidarus Salah[1], and Christopher J. Schofield[1,*]

[1]Chemistry Research Laboratory, Department of Chemistry and the Ineos Oxford Institute for Antimicrobial Research, University of Oxford, 12 Mansfield Road, OX1 3TA, Oxford, United Kingdom.

[2]Present address: Institute of Natural Medicine, University of Toyama, 2630-Sugitani, 930-0194, Toyama, Japan.

*christopher.schofield@chem.ox.ac.uk

[#]These authors contributed equally to this work.

Table of contents

1. Supplementary Figures 2-45
2. Supplementary Tables 46-49
3. Supplementary Methods 50-54
4. Supplementary References 55

1. Supplementary Figures

Supplementary Figure 1. Synthesis of the 2OG derivatives used in this work. The C3/C4-substituted 2OG derivatives (**S5**) were prepared according to a reported strategy employing cyanosulfur ylids **S3** as intermediates[1]. Cyanosulfur ylids **S3** were prepared by the dehydrative coupling of mono-methyl dicarboxylic acid half-esters (**S1**) with the reported tetrahydrothiophene salt **S2**[2]. The cyanosulfur ylids (**S3**) were oxidized using oxone to afford the corresponding dimethyl dicarboxylic acid esters (**S4**). C3/C4-substituted 2OG derivatives (**S5**) were obtained via lithium hydroxide-mediated saponification of dimethyl esters **S4**, in sufficient purity to be employed in biochemical and crystallographic experiments. Note that 2OG derivatives **11** and **22** have not previously been reported.

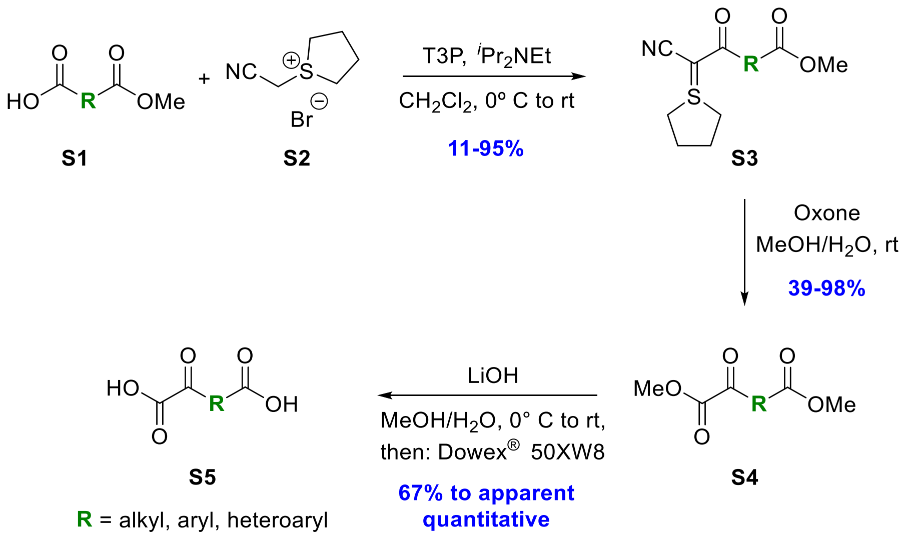

Supplementary Figure 2. FIH catalyzes the oxidative decarboxylation of 2OG to give succinate. FIH-catalyzed oxidative decarboxylation of 2OG was monitored using a Bruker AVIII 700 NMR machine equipped with a 5 mm $^{1}H(^{13}C/^{15}N)$ inverse cryoprobe; spectra were analyzed using the Bruker TopSpin 3.6.1 software. Assay conditions: 5 µM FIH, 150 µM synthetic consensus ankyrin (CA_{1-20}) substrate peptide[3], 150 µM 2OG, and 50 µM ammonium iron(II) sulfate hexahydrate (FAS, $(NH_4)_2Fe(SO_4)_2 \cdot 6H_2O$) in 50 mM aqueous Tris-$d_{11}$ containing $10\%_{v/v}$ D_2O (pH 7.5, 25° C). The ratio of the CA_{1-20} peptide and the hydroxylated CA_{1-20}(Ox) peptide, which is the product of the FIH-catalyzed hydroxylation reaction, was determined using SPE-MS after NMR analysis indicated full conversion of the FIH cosubstrate.

(**a**) Close-up (3.0-2.2 ppm) of the ^{1}H NMR spectrum of commercial 2OG in 50 mM aqueous Tris-d_{11} containing $10\%_{v/v}$ D_2O (pH 7.5, 25° C); (**b**) Close-up (3.0-2.2 ppm) of the ^{1}H NMR spectrum of commercial succinate in 50 mM aqueous Tris-d_{11} containing $10\%_{v/v}$ D_2O (pH 7.5, 25° C); (**c**) ^{1}H NMR analysis reveals ~70% FIH-catalyzed conversion of 2OG to succinate within 5 min; (**d**) Analysis of the final reaction mixture (t = 10 min) by SPE-MS reveals apparently complete hydroxylation (>95% conversion) of the CA_{1-20} peptide (m/z = 1089.6 corresponds to the +2 charge state of hydroxylated CA_{1-20}(Ox); m/z = 1081.6 corresponds to the +2 charge state of CA_{1-20}), indicating that approximately equimolar amounts of succinate and hydroxylated CA_{1-20}(Ox) peptide are present in the final reaction mixture. Thus, the FIH-catalyzed oxidative decarboxylation of 2OG to give succinate is highly coupled with FIH-catalyzed substrate hydroxylation.

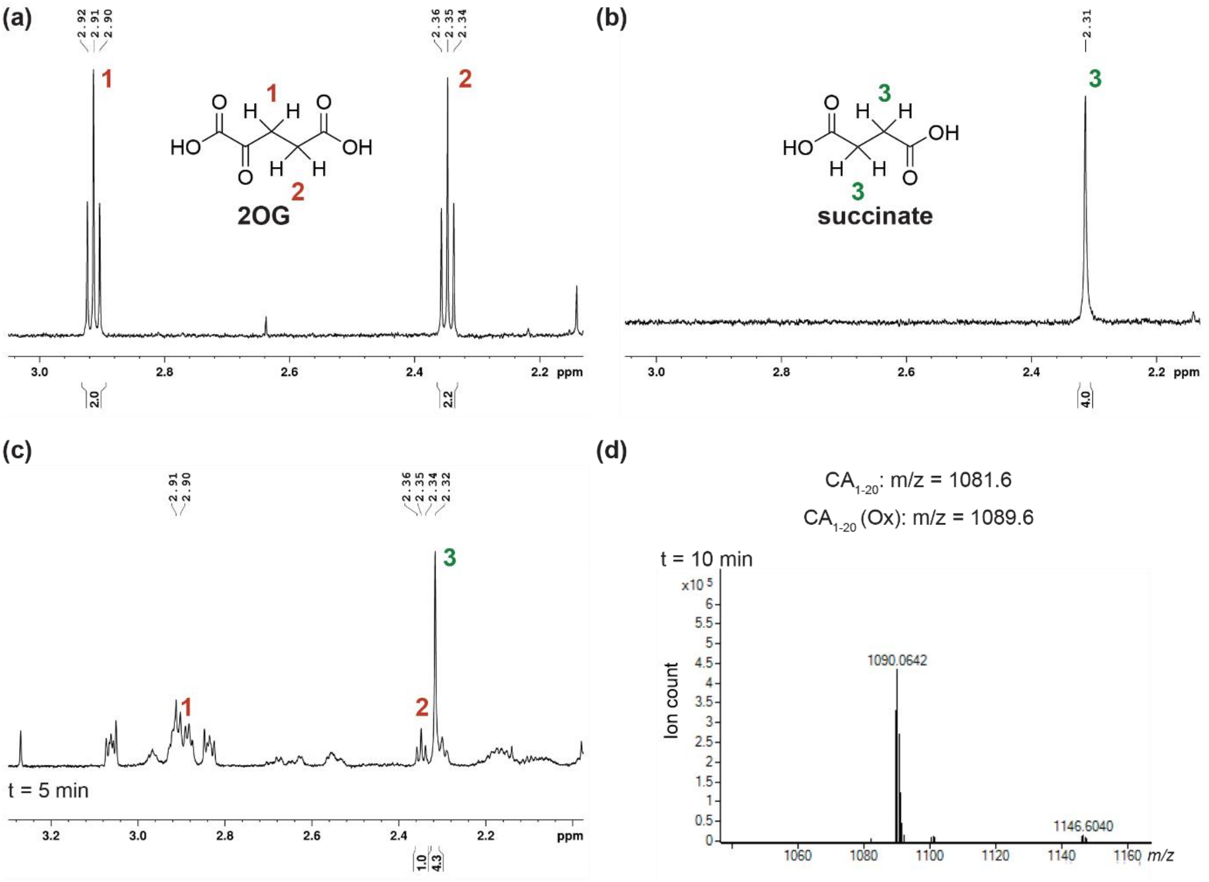

Supplementary Figure 3. FIH catalyzes the oxidative decarboxylation of 3-methyl-2OG to give 2-methylsuccinate. FIH-catalyzed oxidative decarboxylation of 3-methyl-2OG (**1**) was monitored using a Bruker AVIII 700 NMR machine equipped with a 5 mm $^{1}H(^{13}C/^{15}N)$ inverse cryoprobe; spectra were analyzed using the Bruker TopSpin 3.6.1 software. Assay conditions: 5 μM FIH, 150 μM synthetic consensus ankyrin ($CA_{1\text{-}20}$) substrate peptide[3], 150 μM **1**, and 50 μM ammonium iron(II) sulfate hexahydrate (FAS, $(NH_4)_2Fe(SO_4)_2{\cdot}6H_2O$) in 50 mM aqueous Tris-$d_{11}$ containing 10%$_{v/v}$ D_2O (pH 7.5, 25° C). The ratio of the $CA_{1\text{-}20}$ peptide and the hydroxylated $CA_{1\text{-}20}$(Ox) peptide, which is the product of the FIH-catalyzed hydroxylation reaction, was determined using SPE-MS after NMR analysis indicated full conversion of the FIH cosubstrate.

(**a**) Close-up (3.4-0.9 ppm) of the ^{1}H NMR spectrum of synthetic **1** in 50 mM aqueous Tris-d_{11} containing 10%$_{v/v}$ D_2O (pH 7.5, 25° C); (**b**) Close-up (3.4-0.9 ppm) of the ^{1}H NMR spectrum of commercial 2-methylsuccinate (**36**) in 50 mM aqueous Tris-d_{11} containing 10%$_{v/v}$ D_2O (pH 7.5, 25° C); (**c**) ^{1}H NMR analysis reveals ~70% FIH-catalyzed conversion of **1** to **36** within 5 min; (**d**) Analysis of the final reaction mixture (t = 10 min) by SPE-MS reveals high level of hydroxylation (>90% conversion) of the $CA_{1\text{-}20}$ peptide (m/z = 1089.6 corresponds to the +2 charge state of hydroxylated $CA_{1\text{-}20}$(Ox); m/z = 1081.6 corresponds to the +2 charge state of $CA_{1\text{-}20}$). This indicates the presence of approximately equimolar amounts of **36** and the hydroxylated $CA_{1\text{-}20}$(Ox) peptide in the final reaction mixture, as the same concentration of **1** and $CA_{1\text{-}20}$ was employed (150 μM). Thus, the FIH-catalyzed oxidative decarboxylation of **1** to **36** is highly coupled with FIH-catalyzed substrate hydroxylation.

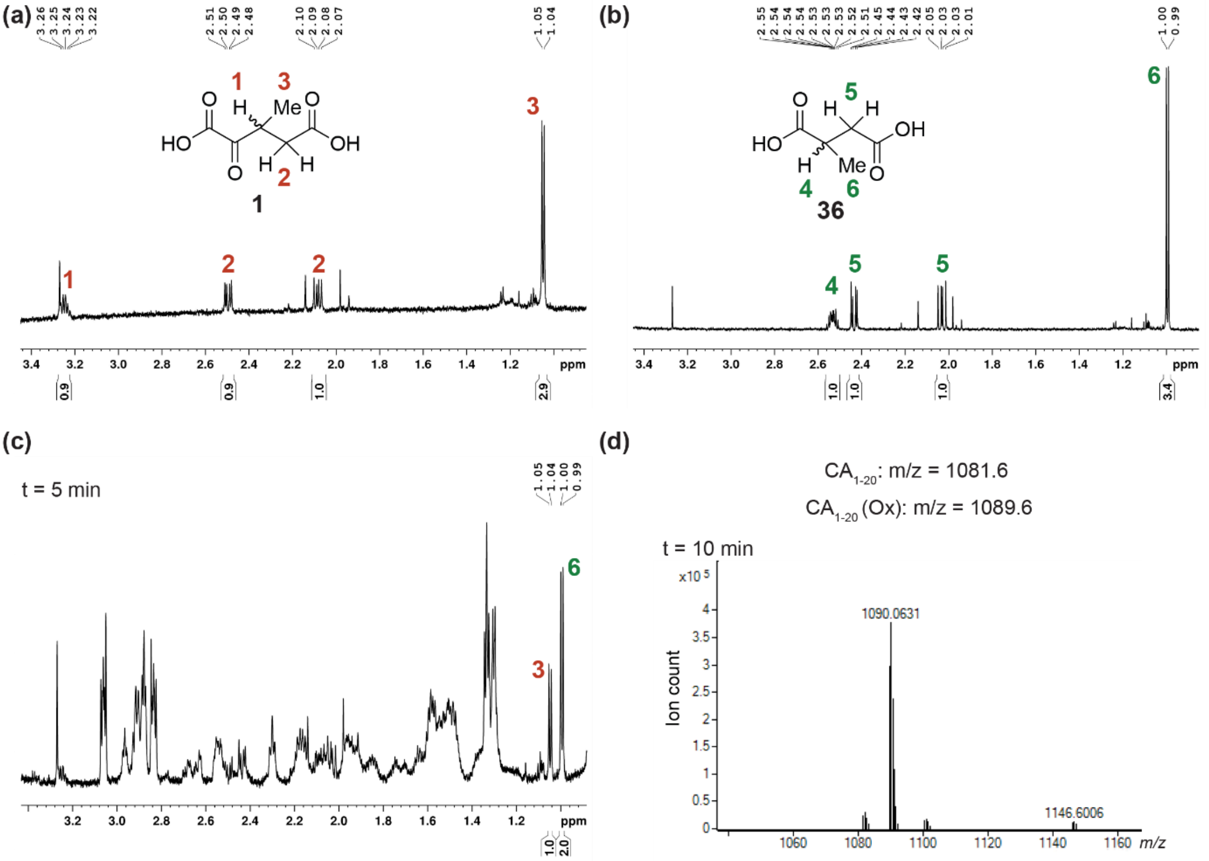

Supplementary Figure 4. FIH catalyzes the oxidative decarboxylation of 4-methyl-2OG to give 2-methylsuccinate. FIH-catalyzed oxidative decarboxylation of 4-methyl-2OG (**12**) was monitored using a Bruker AVIII 700 NMR machine equipped with a 5 mm $^{1}H(^{13}C/^{15}N)$ inverse cryoprobe; spectra were analyzed using the Bruker TopSpin 3.6.1 software. Assay conditions: 5 µM FIH, 150 µM synthetic consensus ankyrin (CA_{1-20}) substrate peptide[3], 150 µM **12**, and 50 µM ammonium iron(II) sulfate hexahydrate (FAS, $(NH_4)_2Fe(SO_4)_2{\cdot}6H_2O$) in 50 mM aqueous Tris-$d_{11}$ containing $10\%_{v/v}$ D_2O (pH 7.5, 25° C). The ratio of the CA_{1-20} peptide and the hydroxylated CA_{1-20}(Ox) peptide, which is the product of the FIH-catalyzed hydroxylation reaction, was determined using SPE-MS after NMR analysis indicated full conversion of the FIH cosubstrate.

(**a**) Close-up (3.3-0.9 ppm) of the ^{1}H NMR spectrum of synthetic **12** in 50 mM aqueous Tris-d_{11} containing $10\%_{v/v}$ D_2O (pH 7.5, 25° C); (**b**) Close-up (3.4-0.9 ppm) of the ^{1}H NMR spectrum of commercial 2-methylsuccinate (**36**) in 50 mM aqueous Tris-d_{11} containing $10\%_{v/v}$ D_2O (pH 7.5, 25° C); (**c**) ^{1}H NMR analysis reveals ~65% FIH-catalyzed conversion of **12** to **36** within 5 min; (**d**) Analysis of the final reaction mixture (t = 10 min) by SPE-MS reveals apparently complete hydroxylation (>95% conversion) of the CA_{1-20} peptide (m/z = 1089.6 corresponds to the +2 charge state of hydroxylated CA_{1-20}(Ox); m/z = 1081.6 corresponds to the +2 charge state of CA_{1-20}). This indicates the presence of approximately equimolar amounts of **36** and the hydroxylated CA_{1-20}(Ox) peptide in the final reaction mixture, as the same concentration of **12** and CA_{1-20} was employed (150 µM). Thus, the FIH-catalyzed oxidative decarboxylation of **12** to give **36** is highly coupled with FIH-catalyzed substrate hydroxylation.

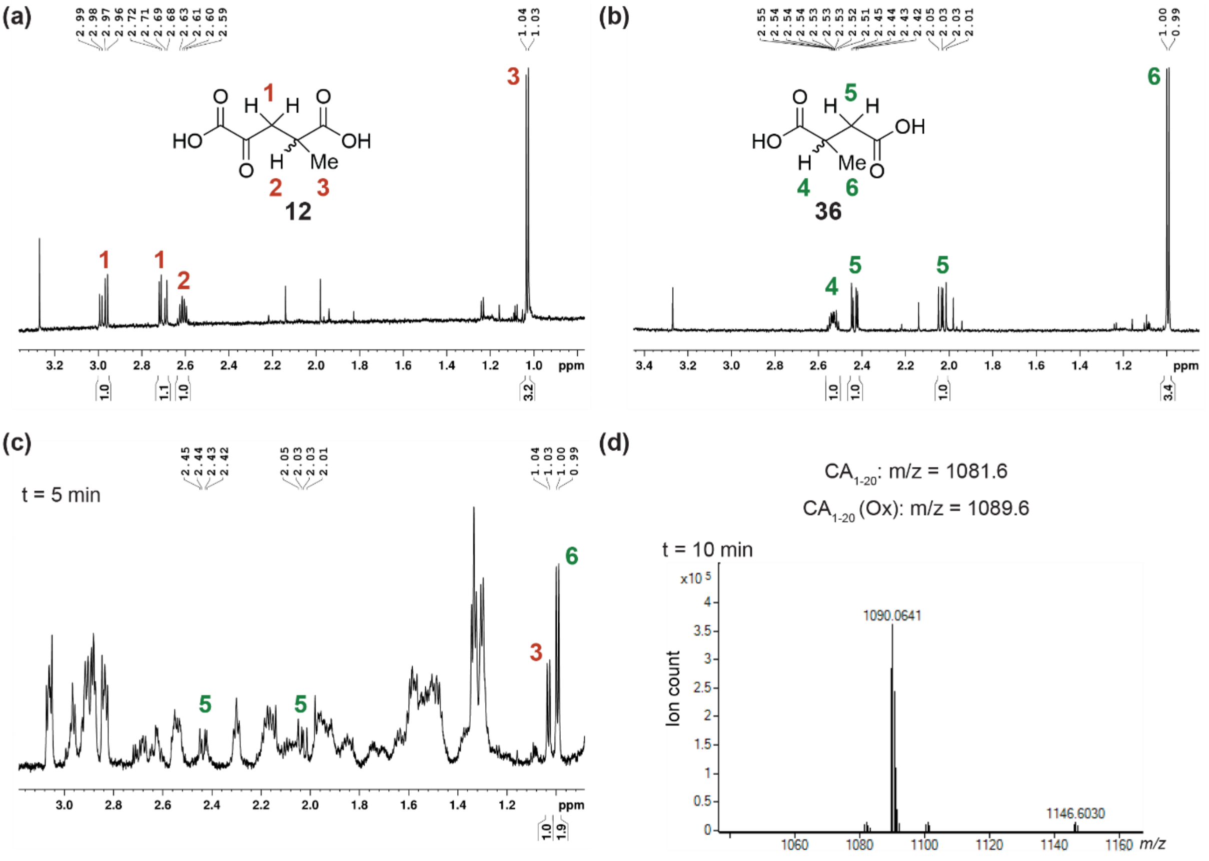

Supplementary Figure 5. Hydroxylation rates of FIH-catalyzed HIF-1α$_{788-822}$ hydroxylations used to determine kinetic parameters for 2OG derivatives (continues on the following page). Maximum velocities (v_{max}^{app}) and Michaelis constants (K_m^{app}) of FIH were determined in independent triplicates for 2OG and the 2OG derivatives **1**, **12**, **14**, and **22**, monitoring FIH-catalyzed hydroxylation of the HIF-1α$_{788-822}$[4] substrate peptide by SPE-MS as described in the Methods section. Conditions: 0.15 µM FIH, 5.0 µM HIF-1α$_{788-822}$[4], 100 µM L-ascorbic acid (LAA), and 20 µM ammonium iron(II) sulfate hexahydrate (FAS, $(NH_4)_2Fe(SO_4)_2 \cdot 6H_2O$) in buffer (50 mM Tris, 50 mM NaCl, pH 7.5, 20° C). Measurement times were normalized to the first sample injection analyzed after the addition of FIH to the Substrate Mixture (t = 0 s), by which time low levels of hydroxylation were manifest. Data are shown as the mean of three independent runs (n = 3; mean ± standard deviation, SD). Source data are provided as a Source Data file.

(**a**) Time course of the FIH-catalyzed hydroxylation reaction of the HIF-1α$_{788-822}$ peptide for the shown concentrations of 2OG; (**b**) hydroxylation rates used to determine kinetic parameters of FIH for 2OG; (**c**) time course of the FIH-catalyzed hydroxylation reaction of the HIF-1α$_{788-822}$ peptide for the shown concentrations of 3-methyl-2OG (**1**); (**d**) hydroxylation rates used to determine kinetic parameters of FIH for **1**; (**e**) time course of the FIH-catalyzed hydroxylation reaction of the HIF-1α$_{788-822}$ peptide for the shown concentrations of 4-methyl-2OG (**12**); (**f**) hydroxylation rates used to determine kinetic parameters of FIH for **12**; (**g**) time course of the FIH-catalyzed hydroxylation reaction of the HIF-1α$_{788-822}$ peptide for the shown concentrations of 4-ethyl-2OG (**14**); (**h**) hydroxylation rates used to determine kinetic parameters of FIH for **14**; (**i**) time course of the FIH-catalyzed hydroxylation reaction of the HIF-1α$_{788-822}$ peptide for the shown concentrations of 3-(carboxycarbonyl)cyclopentane-1-carboxylic acid (**22**); (**j**) hydroxylation rates used to determine kinetic parameters of FIH for **22**.

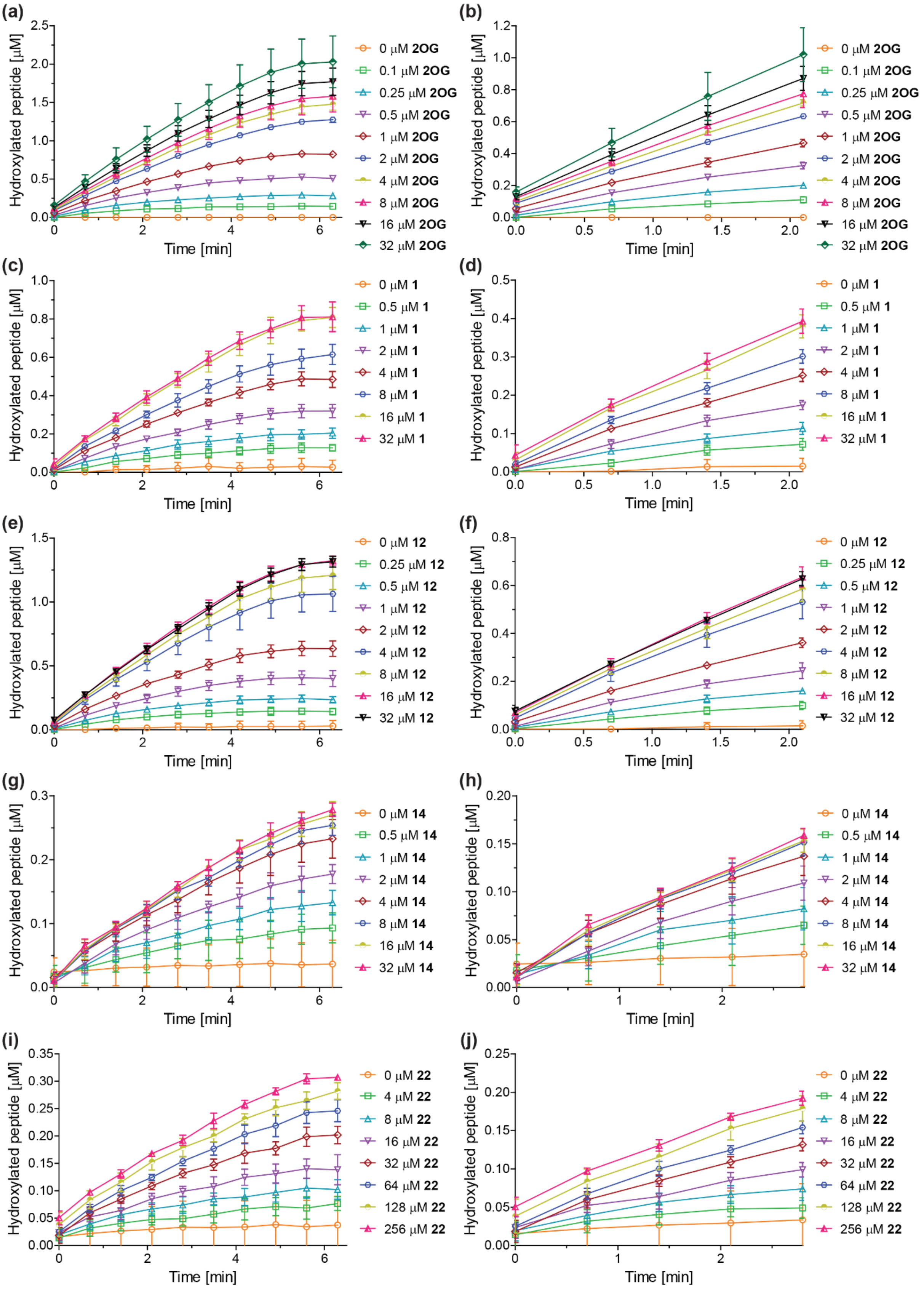
(a)
Hydroxylated peptide [μM]
Time [min]
0 μM 2OG
0.1 μM 2OG
0.25 μM 2OG
0.5 μM 2OG
1 μM 2OG
2 μM 2OG
4 μM 2OG
8 μM 2OG
16 μM 2OG
32 μM 2OG
(b)
Hydroxylated peptide [μM]
Time [min]
0 μM 2OG
0.1 μM 2OG
0.25 μM 2OG
0.5 μM 2OG
1 μM 2OG
2 μM 2OG
4 μM 2OG
8 μM 2OG
16 μM 2OG
32 μM 2OG
(c)
Hydroxylated peptide [μM]
Time [min]
0 μM 1
0.5 μM 1
1 μM 1
2 μM 1
4 μM 1
8 μM 1
16 μM 1
32 μM 1
(d)
Hydroxylated peptide [μM]
Time [min]
0 μM 1
0.5 μM 1
1 μM 1
2 μM 1
4 μM 1
8 μM 1
16 μM 1
32 μM 1
(e)
Hydroxylated peptide [μM]
Time [min]
0 μM 12
0.25 μM 12
0.5 μM 12
1 μM 12
2 μM 12
4 μM 12
8 μM 12
16 μM 12
32 μM 12
(f)
Hydroxylated peptide [μM]
Time [min]
0 μM 12
0.25 μM 12
0.5 μM 12
1 μM 12
2 μM 12
4 μM 12
8 μM 12
16 μM 12
32 μM 12
(g)
Hydroxylated peptide [μM]
Time [min]
0 μM 14
0.5 μM 14
1 μM 14
2 μM 14
4 μM 14
8 μM 14
16 μM 14
32 μM 14
(h)
Hydroxylated peptide [μM]
Time [min]
0 μM 14
0.5 μM 14
1 μM 14
2 μM 14
4 μM 14
8 μM 14
16 μM 14
32 μM 14
(i)
Hydroxylated peptide [μM]
Time [min]
0 μM 22
4 μM 22
8 μM 22
16 μM 22
32 μM 22
64 μM 22
128 μM 22
256 μM 22
(j)
Hydroxylated peptide [μM]
Time [min]
0 μM 22
4 μM 22
8 μM 22
16 μM 22
32 μM 22
64 μM 22
128 μM 22
256 μM 22

Supplementary Figure 6. Hydroxylation rates of FIH-catalyzed CA_{1-20} hydroxylations used to determine kinetic parameters for 2OG derivatives (continues on the following page). Maximum velocities (v_{max}^{app}) and Michaelis constants (K_m^{app}) of FIH were determined in independent triplicates for 2OG and the 2OG derivatives **1**, **12**, **14**, and **22**, monitoring FIH-catalyzed hydroxylation of the CA_{1-20}[3] substrate peptide by SPE-MS as described in the Methods section. Conditions: 0.15 µM FIH, 5.0 µM CA_{1-20}[3], 100 µM L-ascorbic acid (LAA), and 20 µM ammonium iron(II) sulfate hexahydrate (FAS, $(NH_4)_2Fe(SO_4)_2 \cdot 6H_2O$) in buffer (50 mM Tris, 50 mM NaCl, pH 7.5, 20° C). Measurement times were normalized to the first sample injection analyzed after the addition of FIH to the Substrate Mixture (t = 0 s), by which time low levels of hydroxylation were manifest. Data are shown as the mean of three independent runs (n = 3; mean ± standard deviation, SD). Source data are provided as a Source Data file.

(**a**) Time course of the FIH-catalyzed hydroxylation reaction of the CA_{1-20} peptide for the shown concentrations of 2OG; (**b**) hydroxylation rates used to determine kinetic parameters of FIH for 2OG; (**c**) time course of the FIH-catalyzed hydroxylation reaction of the CA_{1-20} peptide for the shown concentrations of 3-methyl-2OG (**1**); (**d**) hydroxylation rates used to determine kinetic parameters of FIH for **1**; (**e**) time course of the FIH-catalyzed hydroxylation reaction of the CA_{1-20} peptide for the shown concentrations of 4-methyl-2OG (**12**); (**f**) hydroxylation rates used to determine kinetic parameters of FIH for **12**; (**g**) time course of the FIH-catalyzed hydroxylation reaction of the CA_{1-20} peptide for the shown concentrations of 4-ethyl-2OG (**14**); (**h**) hydroxylation rates used to determine kinetic parameters of FIH for **14**; (**i**) time course of the FIH-catalyzed hydroxylation reaction of the CA_{1-20} peptide for the shown concentrations of 3-(carboxycarbonyl)cyclopentane-1-carboxylic acid (**22**); (**j**) hydroxylation rates used to determine kinetic parameters of FIH for **22**.

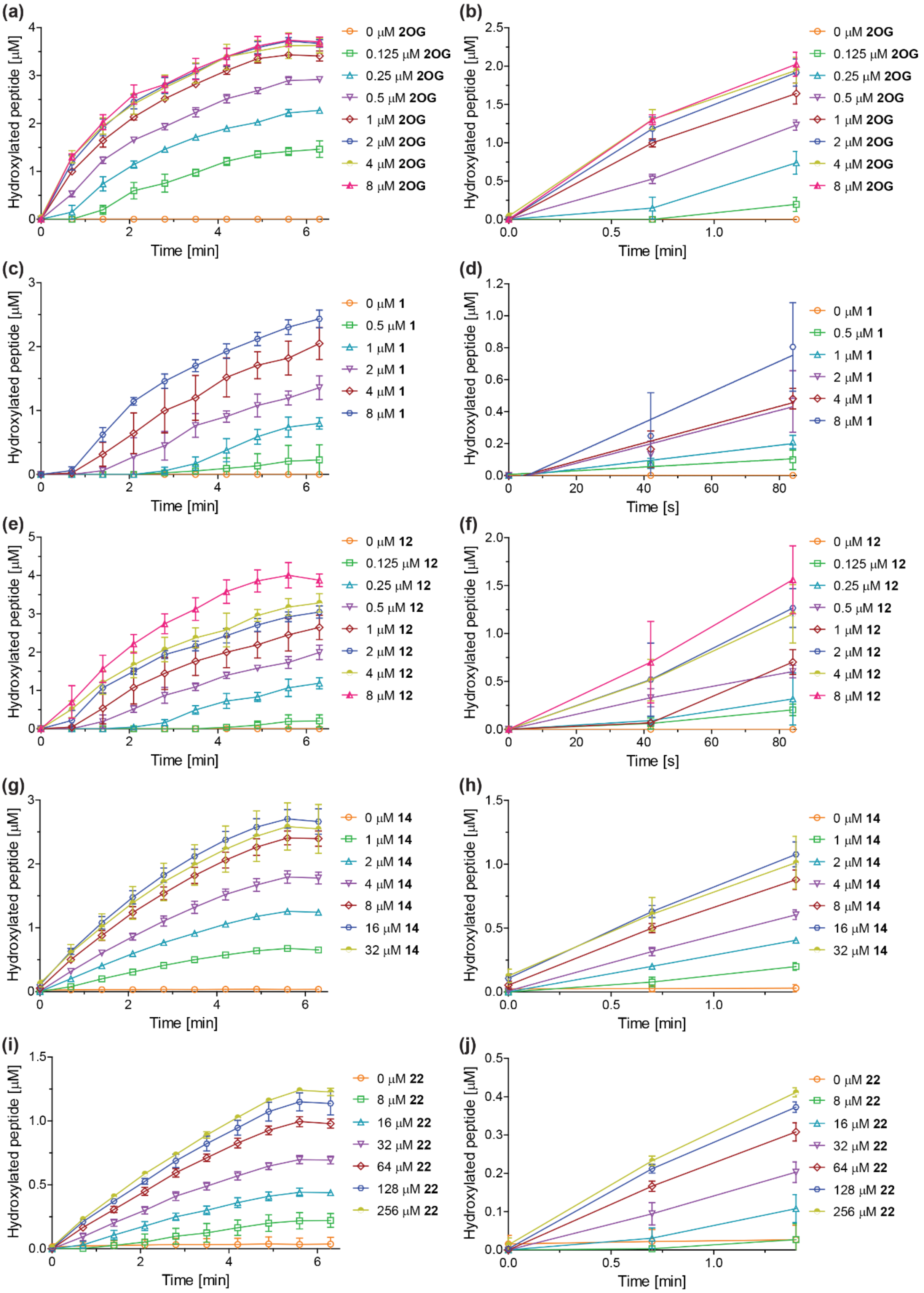
(a)
Hydroxylated peptide [μM]
Time [min]
0 μM 2OG
0.125 μM 2OG
0.25 μM 2OG
0.5 μM 2OG
1 μM 2OG
2 μM 2OG
4 μM 2OG
8 μM 2OG
(b)
Hydroxylated peptide [μM]
Time [min]
0 μM 2OG
0.125 μM 2OG
0.25 μM 2OG
0.5 μM 2OG
1 μM 2OG
2 μM 2OG
4 μM 2OG
8 μM 2OG
(c)
Hydroxylated peptide [μM]
Time [min]
0 μM 1
0.5 μM 1
1 μM 1
2 μM 1
4 μM 1
8 μM 1
(d)
Hydroxylated peptide [μM]
Time [s]
0 μM 1
0.5 μM 1
1 μM 1
2 μM 1
4 μM 1
8 μM 1
(e)
Hydroxylated peptide [μM]
Time [min]
0 μM 12
0.125 μM 12
0.25 μM 12
0.5 μM 12
1 μM 12
2 μM 12
4 μM 12
8 μM 12
(f)
Hydroxylated peptide [μM]
Time [s]
0 μM 12
0.125 μM 12
0.25 μM 12
0.5 μM 12
1 μM 12
2 μM 12
4 μM 12
8 μM 12
(g)
Hydroxylated peptide [μM]
Time [min]
0 μM 14
1 μM 14
2 μM 14
4 μM 14
8 μM 14
16 μM 14
32 μM 14
(h)
Hydroxylated peptide [μM]
Time [min]
0 μM 14
1 μM 14
2 μM 14
4 μM 14
8 μM 14
16 μM 14
32 μM 14
(i)
Hydroxylated peptide [μM]
Time [min]
0 μM 22
8 μM 22
16 μM 22
32 μM 22
64 μM 22
128 μM 22
256 μM 22
(j)
Hydroxylated peptide [μM]
Time [min]
0 μM 22
8 μM 22
16 μM 22
32 μM 22
64 μM 22
128 μM 22
256 μM 22

Supplementary Figure 7. FIH active site titration. The concentration of active FIH was investigated by titration with the reported inhibitor *N*-oxalyl-D-phenylalanine (NOFD[5]). SPE-MS was used to monitor the hydroxylation of the HIF-1$\alpha_{788\text{-}822}$ peptide[4] in buffer (50 mM Tris, 50 mM NaCl, pH 7.5, 20 °C) using identical MS configurations as described in the Methods section. Data are shown as the mean of three independent runs (n = 3; mean ± standard deviation, SD). Source data are provided as a Source Data file. (**a**) Time course of the FIH-catalyzed hydroxylation reaction of HIF-1$\alpha_{788\text{-}822}$ for the shown NOFD concentrations using 0.3 µM FIH, 5.0 µM HIF-1$\alpha_{788\text{-}822}$, 100 µM LAA, 10.0 µM FAS, and 10.0 µM 2OG; (**b**) hydroxylation rates used to determine the concentration of FIH active sites. Measurement times were normalized to the first sample injection analyzed after the addition of FIH to the Substrate Mixture (t = 0 s), by which time low levels of hydroxylation were manifest; (**c**) Morrison plot used to determine the concentration of FIH active sites. The slopes of the initial reaction rates were fitted to the Morrison equation using non-linear regression (GraphPad Prism 5) with the following constraints: 0 < enzyme active sites ($[E]_T$) < 0.3 µM; K_m^{app} (2OG) = 0.84 µM; concentration (2OG) = 10.0 µM. The Morrison equation (I: inhibitor concentration; K_i: dissociation constant of inhibitor; E_T: total concentration of active enzyme; v_i/v_0: fractional enzyme activity)[6]:

$$\frac{v_i}{v_0} = \left(1 - \frac{[E]_T + [I] + (K_i \bullet (1 + [S]/K_{m,app}) - \sqrt{([E]_T + [I] + K_i \bullet (1 + [S]/K_{m,app}))^2 - 4[E]_T[I]}}{2[E]_T} \right)$$

The FIH active site titration reveals apparent quantitative activity of FIH (~0.3 µM active FIH for 0.3 µM FIH used). Note, that NOFD was chosen as FIH inhibitor even though it is not very tight-binding, a decision made due to the lack of reported efficient tight-binding or covalent FIH inhibitors[7].

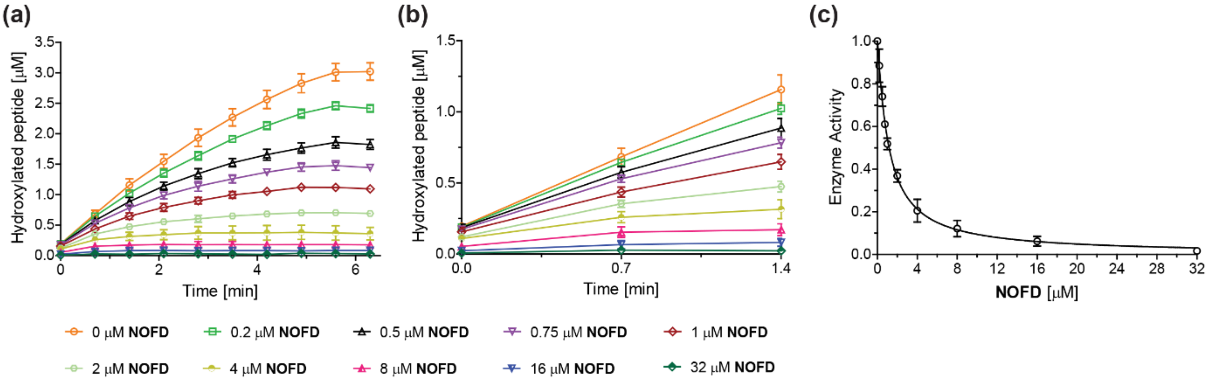

Supplementary Figure 8. 4-Carboxyphenylglyoxylic acid (28) sustains AspH activity selectively in the presence of FIH and in the absence of 2OG.

FIH and AspH-catalyzed peptide hydroxylations were analyzed simultaneously using SPE-MS as described in the Methods section; competition experiments were performed in the same reaction vessel using equimolar 2OG oxygenase and substrate concentrations. Conditions: 0.15 µM FIH, 5.0 µM $CA_{1\text{-}20}$ peptide (FIH substrate)[3], 0.15 µM AspH, 5.0 µM hFX-EGFD$1_{86\text{-}124}$-4Ser peptide (AspH substrate)[8], 100 µM L-ascorbic acid (LAA), 10 µM ammonium iron(II) sulfate hexahydrate (FAS, $(NH_4)_2Fe(SO_4)_2 \cdot 6H_2O$), and 2OG and/or 4-carboxyphenylglyoxylic acid (**28**) in buffer (50 mM Tris, 50 mM NaCl, pH 7.5, 20° C). Measurement times were normalized to the first sample injection analyzed after the addition of the 2OG oxygenases to the Substrate Mixture (t = 0 s), by which time low levels of hydroxylation were manifest. **28** is a reported cosubstrate of AspH (Table 1)[1], and showed weak cosubstrate activity for FIH with $CA_{1\text{-}20}$ (Table 1). Source data are provided as a Source Data file.

28 selectively sustains AspH catalysis (black circles; panel **b**) in the absence of 2OG, while no cosubstrate activity for FIH (orange squares) was observed. However, compared to the natural AspH cosubstrate 2OG (panel **a**), **28** is substantially less efficient in sustaining AspH catalysis (~40% conversion after 35 min, compared to >95% conversion after 2 min with 2OG as a cosubstrate as shown in panel **a**). Note that **28** is able to sustain low levels of FIH catalysis at a higher concentration, *i.e.* 330 µM (Table 1). In the presence of both 2OG (20 µM) and **28** (100 µM), AspH activity is similar as in the absence of **28** (panel **c** and **a**), while FIH activity is substantially reduced (panel **c**); note that an inhibitory effect of **28** on FIH catalysis was not observed under different assay conditions (Supplementary Table 1).

Cosubstrate concentrations: (**a**) 20 µM 2OG, 0 µM **28**; (**b**) 0 µM 2OG, 100 µM **28**; (**c**) 20 µM 2OG, 100 µM **28**.

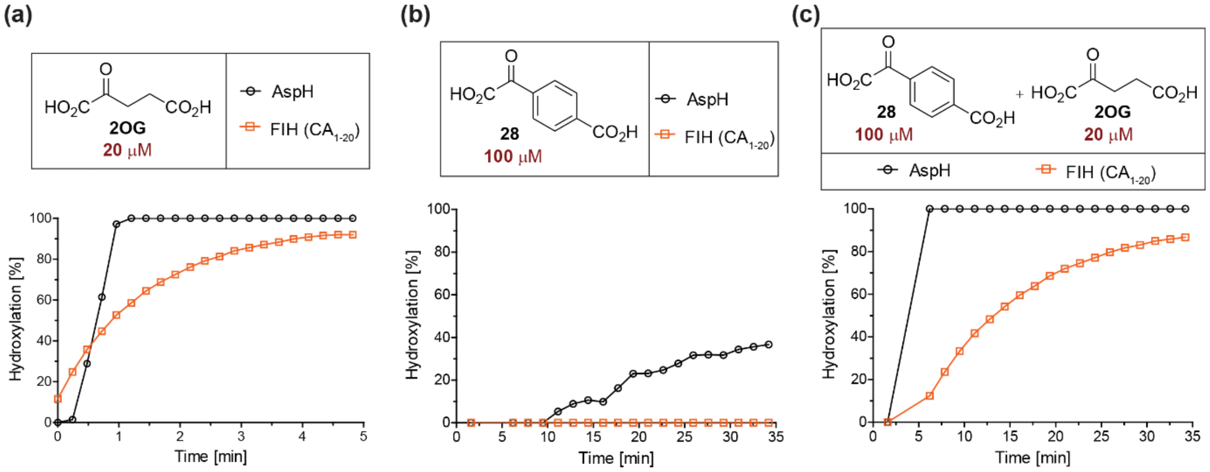

Supplementary Figure 9. Views from a crystal structure of FIH complexed with 3-methyl-2OG (1) and Zn (FIH:1; PDB ID: 7A1L). Color code: FIH: grey; carbon-backbone of 3-methyl-2OG (**1**): yellow; Zn: lavender blue; oxygen: red; nitrogen: blue.

(**a**) Overview of the FIH:**1** crystal structure; (**b**) representative OMIT electron density map (mF_o-DF_c) contoured to 3σ around (*S*)-**1** of the FIH:**1** structure. (*S*)-**1** coordinates to the Zn ion in a bidentate manner and is positioned to interact with the sidechains of FIH active site residues Lys214, Tyr145, Thr196, Asn294, and Asn205 (distances in Å); (**c**) superimposition of a view from the FIH:**1** structure with one from the reported FIH:2OG structure (FIH: pale green, carbon-backbone of 2OG: green, Fe: orange; PDB ID: 1H2N)[9] reveals similar FIH conformations (Cα RMSD = 0.17 Å).

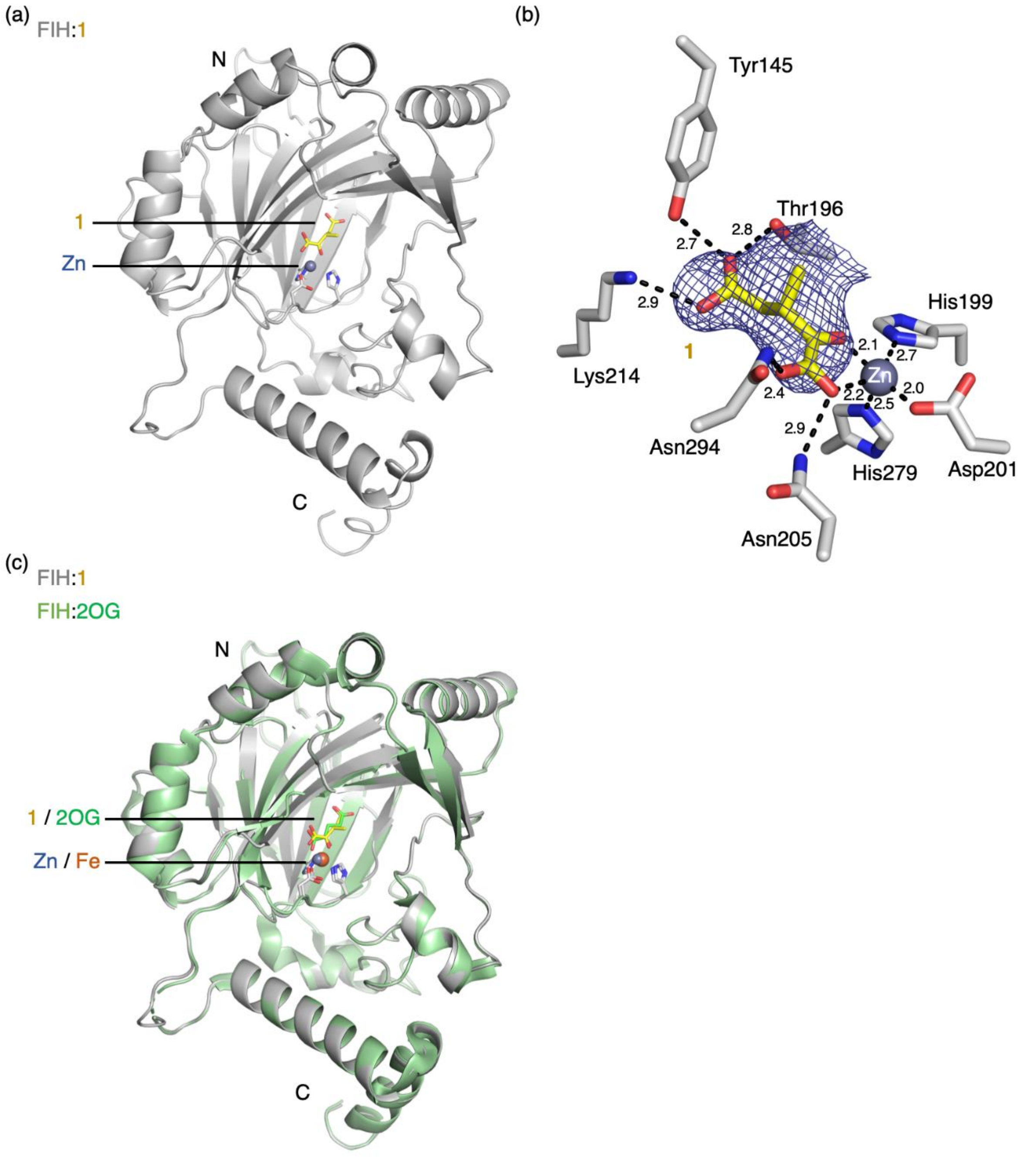

Supplementary Figure 10. Analysis of electron density maps for 3-methyl-2OG (1) in the FIH:1 structure (PDB ID: 7A1L). Color code: FIH: grey; carbon-backbone of (*S*)-3-methyl-2OG ((*S*)-**1**): yellow; carbon-backbone of (*R*)-3-methyl-2OG ((*R*)-**1**): deep green; Zn: lavender blue; water: red sphere; oxygen: red; nitrogen: blue.

(**a**) Representative OMIT electron density map (mF_o-DF_c) contoured to 3σ around (*S*)-**1** modelled in the FIH:**1** structure (blue mesh). Electron density maps (F_o-F_c) contoured to 2σ and -2σ around (*S*)-**1** are shown in green and red mesh, respectively; (**b**) representative OMIT electron density map (mF_o-DF_c) contoured to 3σ around (*R*)-**1** modelled in the FIH:(*R*)-**1** structure (blue mesh). Electron density maps (F_o-F_c) contoured to 2σ and -2σ around (*R*)-**1** are shown in green and red mesh, respectively. Even though the experimental data do not support the predominant presence of either enantiomer in the FIH:**1** structure, (*S*)-**1** was selected for the structure refinement by analogy to the FIH:**1**:CA_{20} and FIH:**1**:$TANK2_{691-710}$ structures, which show clear evidence for the predominant (at least) presence of (*S*)-**1** in the structures (Supplementary Figures 14 and 16). This proposal is consistent with the binding mode of *N*-(carboxycarbonyl)-D-phenylalanine (NOFD) to FIH[5]; Note, that the nitrogen atom of NOFD affects the Cahn-Ingold-Prelog priority rules resulting in its formal assignment as (*R*)-enantiomer.

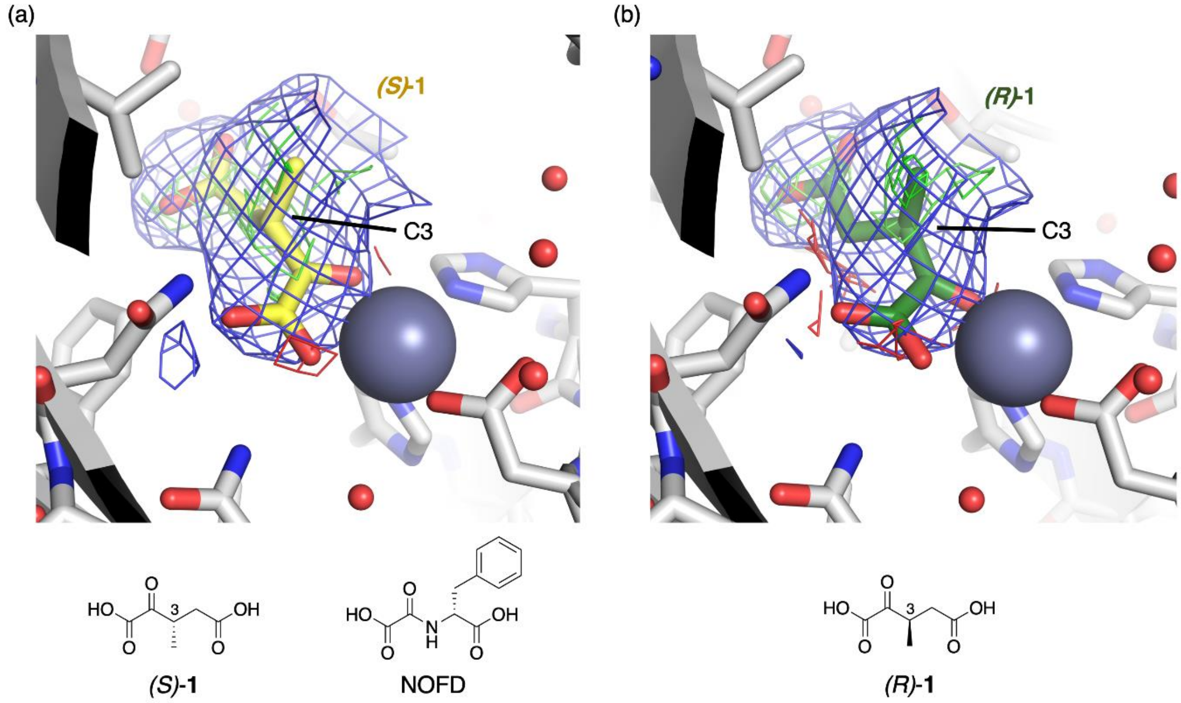

Supplementary Figure 11. Views from a structure of FIH complexed with 3-propyl-2OG (3) and Zn (FIH:3; PDB ID: 7A1M). Color code: FIH: grey; carbon-backbone of 3-propyl-2OG (**3**): orange; Zn: lavender blue; oxygen: red; nitrogen: blue; w: water.

(**a**) Overview of the FIH:**3** structure; (**b**) representative OMIT electron density map (mF_o-DF_c) contoured to 5σ around (*S*)-**3** of the FIH:**3** structure. (*S*)-**3** coordinates to the Zn ion in a bidentate manner and is positioned to interact with the sidechains of FIH active site residues Lys214, Tyr145, Thr196, Asn294, and Asn205 (distances in Å); (**c**) superimposition of a view from the FIH:**3** structure with one from the FIH:**1** structure (FIH: pale yellow, carbon-backbone of **1**: yellow) reveals similar FIH conformations (Cα RMSD = 0.09 Å); (**d**) superimposition of a view from the FIH:**3** structure with one from the reported FIH:2OG:HIF-1$\alpha_{786\text{-}826}$ structure (FIH: pale green, carbon-backbone of 2OG: green, carbon-backbone of the HIF-1$\alpha_{786\text{-}826}$ substrate peptide: cyan, Fe: orange; PDB ID: 1H2L)[9] reveals similar FIH conformations (Cα RMSD = 0.14 Å).

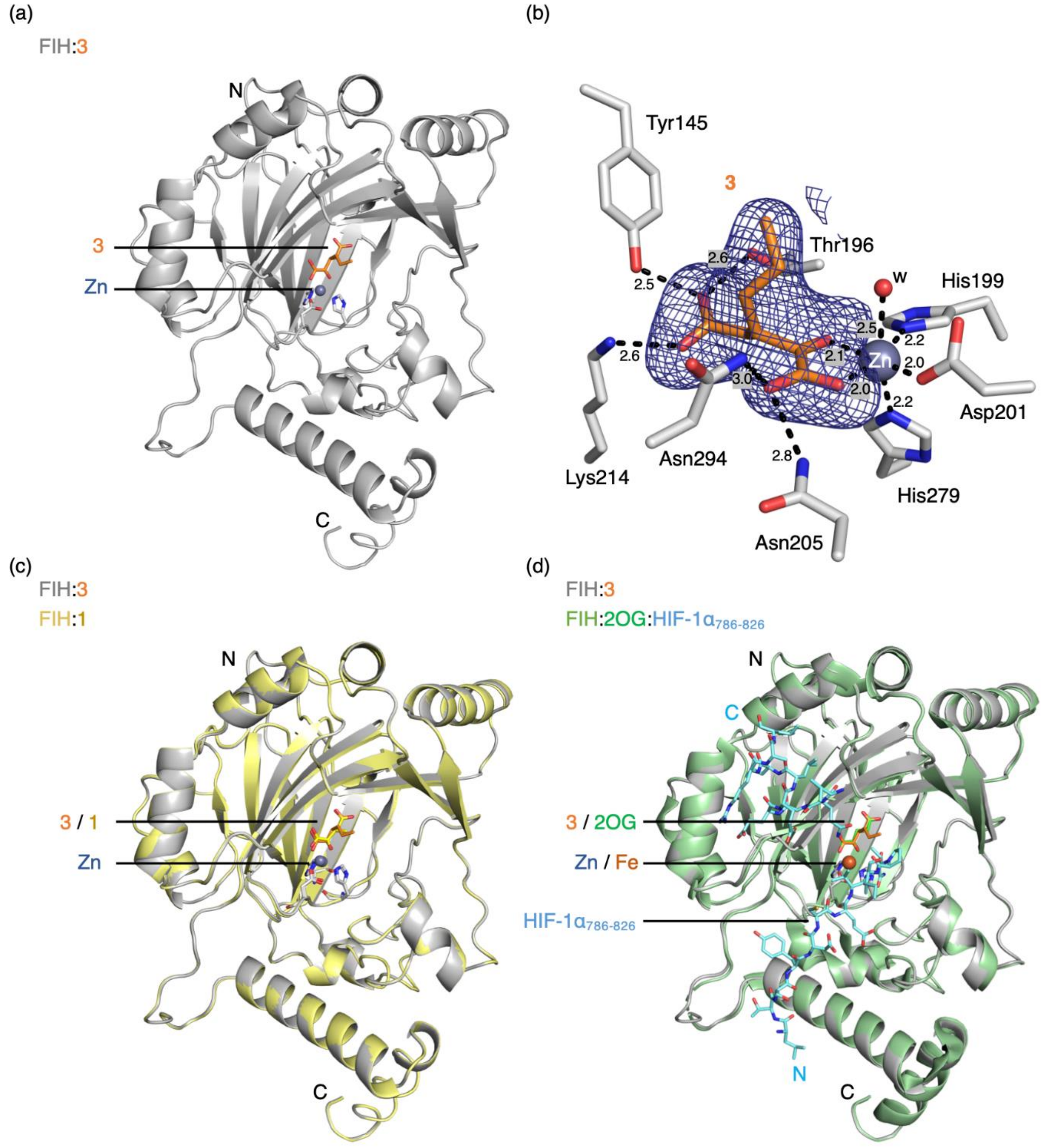

Supplementary Figure 12. Analysis of electron density maps supports the predominant presence of the (*S*)-enantiomer of 3-propyl-2OG (3) in the FIH:3 structure (PDB ID: 7A1M). Color code: FIH: grey; (*S*)-3-propyl-2OG ((*S*)-**3**): orange; carbon-backbone of (*R*)-3-propyl-2OG ((*R*)-**3**): deep green; Zn: lavender blue; water: red sphere; oxygen: red; nitrogen: blue.

(**a**) Representative OMIT electron density map (mF_o-DF_c) contoured to 3σ around (*S*)-**3** modelled in the FIH:(*S*)-**3** structure (blue mesh). Electron density maps (F_o-F_c) contoured to 2σ and -2σ around (*S*)-**3** are shown in green and red mesh, respectively; (**b**) superimposition of (*S*)-**3** and (*R*)-**3** modelled in the FIH:**3** structure. Representative OMIT electron density map (mF_o-DF_c) contoured to 3σ around (*R*)-**3** modelled in the FIH:(*R*)-**3** structure (blue mesh). Electron density maps (F_o-F_c) contoured to 2σ and -2σ around 3σ around (*R*)-**3** are shown in green and red mesh, respectively. Negative densities were observed at the -2σ level around the C3 atom and the alkyl substituent of (*R*)-**3**; these negative densities were not observed at the -2σ level for (*S*)-**3** as shown in panel (**a**). The superimposed image shows that the C3 atom and the alkyl substituent of (*S*)-**3** positioned to compensate for the negative densities observed for (*R*)-**3** in the putative FIH:(*R*)-**3** structure. Thus, the electron density analysis indicates that the (*S*)-enantiomer of **3** is predominantly (at least) present in the FIH:**3** structure. This proposal is consistent with the binding mode of *N*-(carboxycarbonyl)-D-phenylalanine (NOFD) to FIH[5]; Note, that the nitrogen atom of NOFD affects the Cahn-Ingold-Prelog priority rules resulting in its formal assignment as (*R*)-enantiomer.

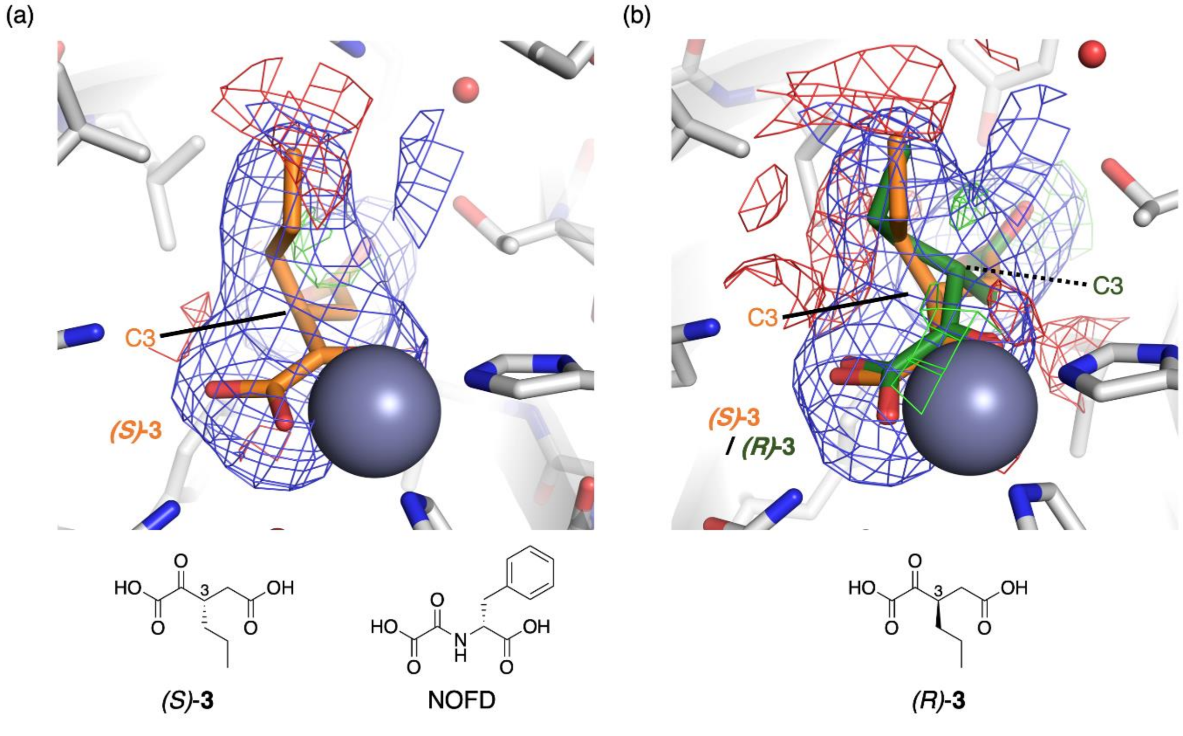

Supplementary Figure 13. Views from a structure of FIH complexed with 3-methyl-2OG (1), Zn, and the synthetic ankyrin peptide CA_{1-20} (FIH:1:CA_{1-20}; PDB ID: 7A1N). Color code: FIH: grey; carbon-backbone of 3-methyl-2OG (**1**): yellow; Zn: lavender blue; carbon-backbone of the synthetic ankyrin peptide CA_{1-20}: orange; oxygen: red; nitrogen: blue.

(**a**) Overview of the FIH:**1**:CA_{1-20} crystal structure; (**b**) representative OMIT electron density map (mF_o-DF_c) contoured to 3σ around (*S*)-**1** of the FIH:**1**:CA_{1-20} structure. (*S*)-**1** coordinates to the Zn ion in a bidentate manner and is positioned to interact with the sidechains of FIH active site residues Lys214, Tyr145, Thr196, Asn294, and Asn205 (distances in Å); (**c**) representative OMIT electron density map (mF_o-DF_c) contoured to 3σ around the synthetic ankyrin peptide CA_{1-20} of the FIH:**1**:CA_{1-20} structure.

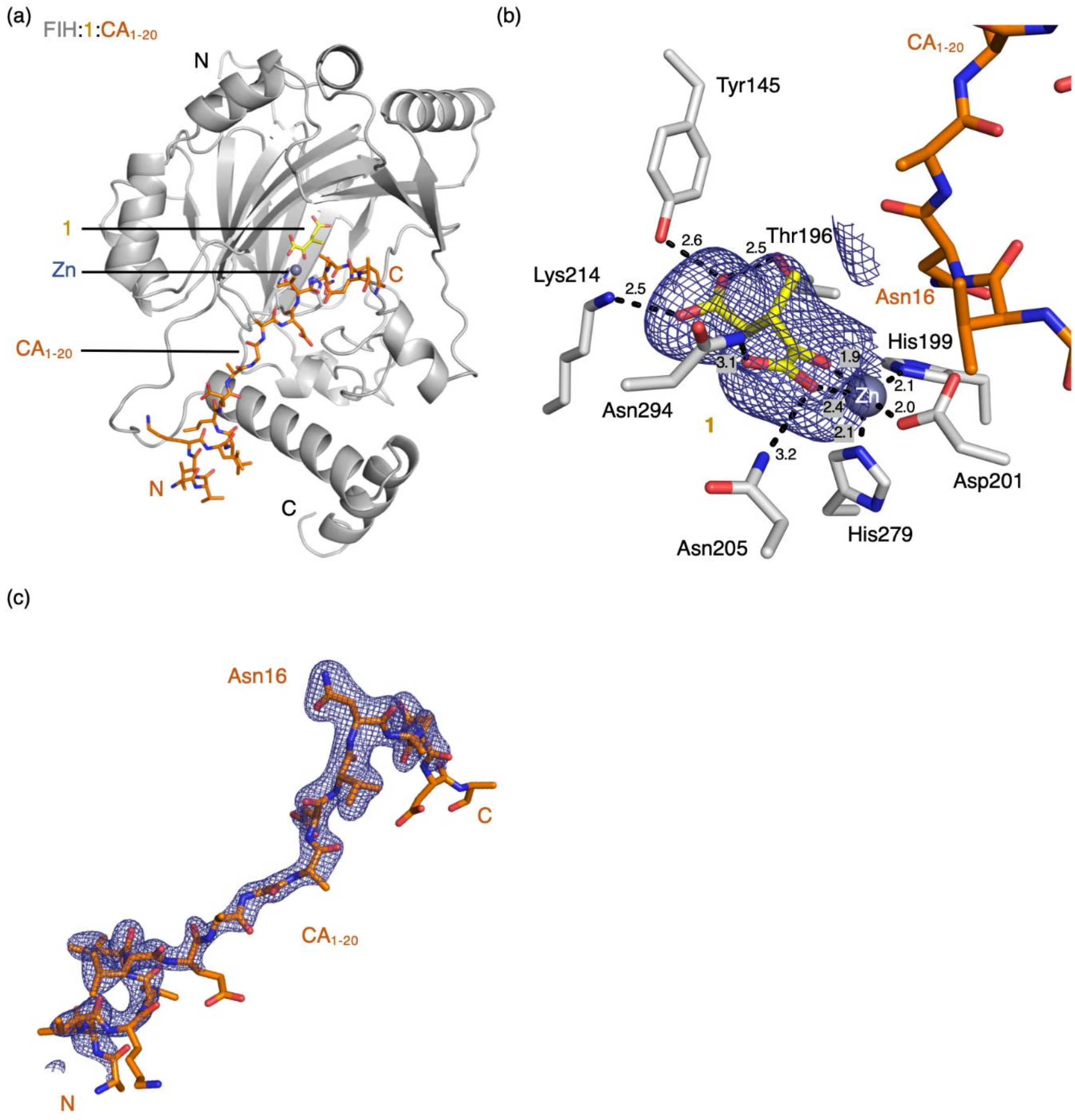

Supplementary Figure 14. Analysis of electron density maps supports the predominant presence of the (*S*)-enantiomer of 3-methyl-2OG (1) in the FIH:1:$CA_{1\text{-}20}$ structure (PDB ID: 7A1N; continues on the following page). Color code: FIH: grey; carbon-backbone of (*S*)-3-methyl-2OG ((*S*)-**1**): yellow; carbon-backbone of (*R*)-3-methyl-2OG ((*R*)-**1**): deep green; Zn: lavender blue; water: red sphere; oxygen: red; nitrogen: blue. Distances are in Å.

(**a**) Representative OMIT electron density map (mF_o-DF_c) contoured to 3σ around (*S*)-**1** modelled in the FIH:**1**:$CA_{1\text{-}20}$ structure (blue mesh). Electron density maps (F_o-F_c) contoured to 2σ and -2σ around (*S*)-**1** are shown in green and red mesh, respectively; (**b**) representative OMIT electron density map (mF_o-DF_c) contoured to 3σ around (*R*)-**1** modelled in the FIH:**1**:$CA_{1\text{-}20}$ structure (blue mesh). Electron density maps (F_o-F_c) contoured to 2σ and -2σ around (*R*)-**1** are shown in green and red mesh, respectively. The methyl carbon atom of (*R*)-**1** is proximate to the Leu188 sidechain (2.9 Å); the van der Waals overlap is ~0.6 Å. This clash with the Leu188 sidechain supports the predominant presence of (*S*)-enantiomer of **1** in the FIH:**1**:$CA_{1\text{-}20}$ structure, as both C6 and C3 carbon atoms of (*S*)-**1** are more than 4.1 Å distant from the Leu188 sidechain. Moreover, the electron density analysis in the FIH:**1**:$TANK2_{691\text{-}710}$ structure supports the predominant binding of (*S*)-**1** in the FIH structures (Supplementary Figure 16). This proposal is consistent with the binding mode of *N*-(carboxycarbonyl)-D-phenylalanine (NOFD) to FIH[5]; Note, that the nitrogen atom of NOFD affects the Cahn-Ingold-Prelog priority rules resulting in its formal assignment as (*R*)-enantiomer; (**c**) superimposition of a view from the FIH:**1**:$CA_{1\text{-}20}$ structure with one from the FIH:**1** structure (FIH: light pink, carbon-backbone of **1**: pink) reveals similar FIH conformations (Cα RMSD = 0.16 Å); (**d**) superimposition of a view from the FIH:**1**:$CA_{1\text{-}20}$ structure with one from the reported FIH:2OG:HIF-$1\alpha_{786\text{-}826}$ structure (FIH: pale green, carbon-backbone of 2OG: green, carbon-backbone of the HIF-$1\alpha_{786\text{-}826}$ substrate peptide: cyan, Fe: orange; PDB ID: 1H2L)[9] reveals similar FIH conformations (Cα RMSD = 0.14 Å); (**e**) superimposition of the $CA_{1\text{-}20}$ peptide from the FIH:**1**:$CA_{1\text{-}20}$ structure with the HIF-$1\alpha_{786\text{-}826}$ peptide from the reported FIH:2OG:HIF-$1\alpha_{786\text{-}826}$ structure[9] (PDB ID: 1H2L). Note, that the conformations of the N-termini of both substrate peptides are similar (Cα RMSD = 0.45 Å, calculated using the 10 Cα atoms of both Leu8 – Ala17 of the $CA_{1\text{-}20}$ peptide and Leu795 – Ala804 of the HIF-$1\alpha_{786\text{-}826}$ peptide), whereas the conformations of the C-termini substantially differ (the HIF-$1\alpha_{786\text{-}826}$ peptide bears an additional 15 C-terminal residues), indicating that substrate residues distant from the FIH active site may have an impact on substrate kinetics.

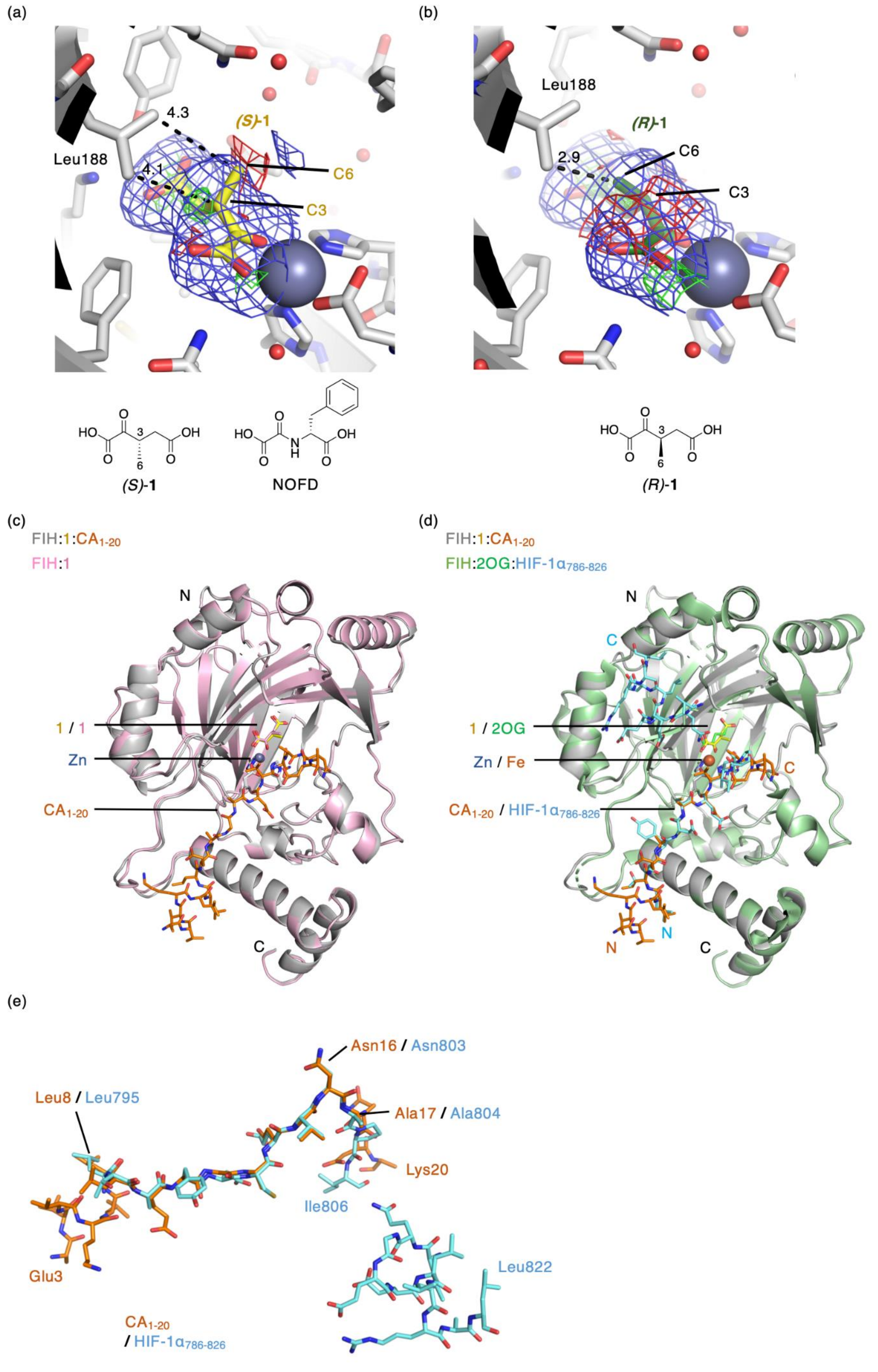
(a)
(b)
Leu188
4.3
4.1
(S)-1
C6
C3
Leu188
2.9
(R)-1
C6
C3
HO
O
3
OH
O
6
O
(S)-1
NOFD
(R)-1
(c)
FIH:1:CA1-20
FIH:1
N
1 / 1
Zn
CA1-20
C
(d)
FIH:1:CA1-20
FIH:2OG:HIF-1α786-826
N
C
1 / 2OG
Zn / Fe
CA1-20 / HIF-1α786-826
C
N
N
C
(e)
Asn16 / Asn803
Leu8 / Leu795
Ala17 / Ala804
Lys20
Ile806
Glu3
Leu822
CA1-20
/ HIF-1α786-826

Supplementary Figure 15. Views from a structure of FIH complexed with 3-methyl-2OG (1), Zn, and the synthetic tankyrase-2 fragment peptide TANK2$_{691\text{-}710}$ (FIH:1:TANK2$_{691\text{-}710}$; PDB ID: 7A1S). Color code: FIH: grey; carbon-backbone of 3-methyl-2OG (**1**): yellow; Zn: lavender blue; carbon-backbone of the tankyrase-2 fragment peptide (TANK2$_{691\text{-}710}$): purple; oxygen: red; nitrogen: blue.

(**a**) Overview of the FIH:**1**:TANK2$_{691\text{-}710}$ crystal structure; (**b**) representative OMIT electron density map (mF_o-DF_c) contoured to 5σ around (*S*)-**1** of the FIH:**1**:TANK2$_{691\text{-}710}$ structure. (*S*)-**1** coordinates to the Zn ion in a bidentate manner and is positioned to interact with the sidechains of FIH active site residues Lys214, Tyr145, Thr196, Asn294, and Asn205 (distances in Å); (**c**) representative OMIT electron density map (mF_o−DF_c) contoured to 3σ around the synthetic tankyrase-2 fragment peptide TANK2$_{691\text{-}710}$ of the FIH:**1**:TANK2$_{691\text{-}710}$ structure.

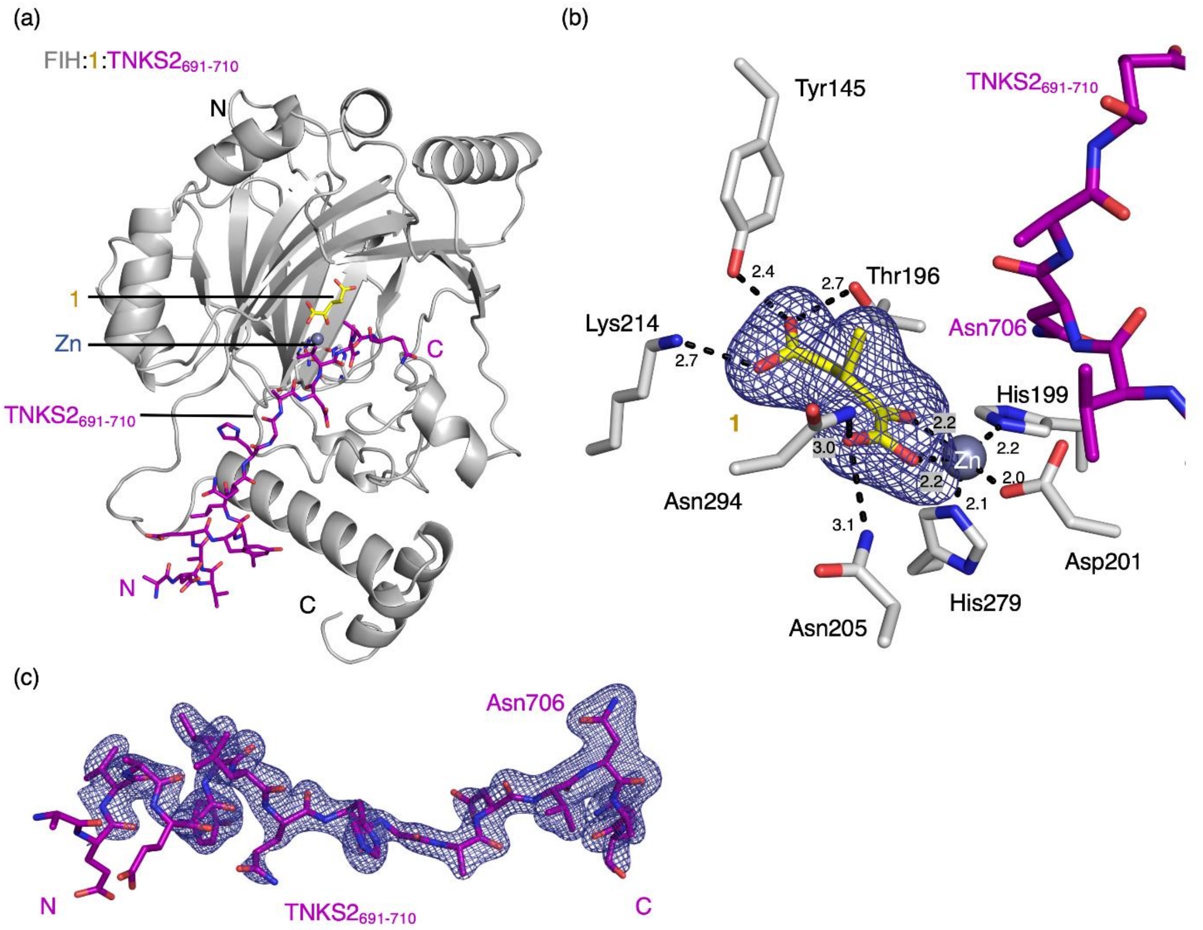

Supplementary Figure 16. Analysis of electron density maps supports the predominant presence of the (*S*)-enantiomer of 3-methyl-2OG (1) in the FIH:1:TANK2$_{691\text{-}710}$ structure (PDB ID: 7A1S). Color code: FIH: grey; carbon-backbone of (*S*)-3-methyl-2OG ((*S*)-**1**): yellow; carbon-backbone of (*R*)-3-methyl-2OG ((*R*)-**1**): deep green; lavender blue: Zn; water: red sphere; oxygen: red; nitrogen: blue. Distances are in Å.

(**a**) Representative OMIT electron density map (mF_o-DF_c) contoured to 3σ around (*S*)-**1** modelled in the FIH:(*S*)-**1**:TANK2$_{691\text{-}710}$ structure (blue mesh). Electron density maps (F_o-F_c) contoured to 2σ and -2σ around (*S*)-**1** are shown in green and red mesh, respectively; (**b**) superimposition of (*S*)-**1** and (*R*)-**1** modelled in the FIH:**1**:TANK2$_{691\text{-}710}$ structure. Representative OMIT electron density map (mF_o-DF_c) contoured to 3σ around (*R*)-**1** modelled in the FIH:(*R*)-**1**:TANK2$_{691\text{-}710}$ structure (blue mesh). Electron density maps (F_o-F_c) contoured to 2σ and -2σ around (*R*)-**1** are shown in green and red mesh, respectively. The methyl carbon atom of (*R*)-**1** is close to the Leu188 sidechain (2.9 Å); the van der Waals overlap is ~0.6 Å. Both positive and negative densities were observed around the C3 and C6 atoms of (*R*)-**1**, but not for (*S*)-**1** as shown in panel (**a**). The superimposed image shows that the C3 and C6 atoms of (*S*)-**1** are positioned to avoid a clash with the Leu188 sidechain; positive and negative densities were not observed. Thus, the electron density analysis supposed the predominant (at least) presence of the (*S*)-enantiomer of **1** in the FIH:**1**:TANK2$_{691\text{-}710}$ structure.

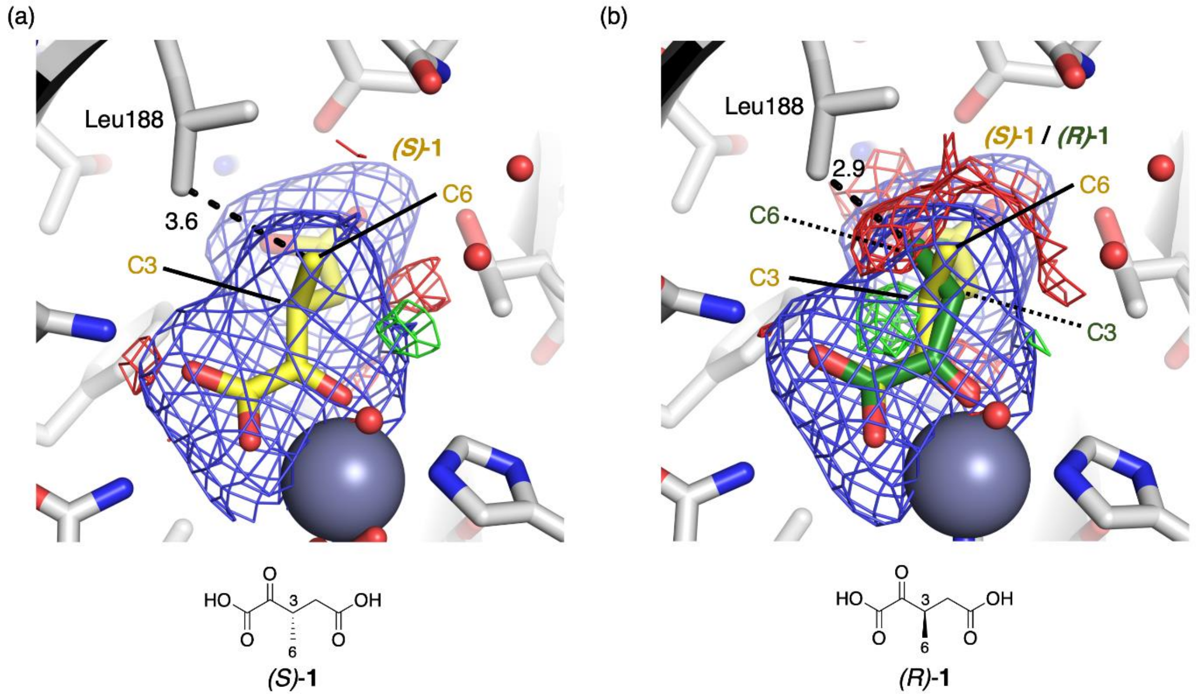

Supplementary Figure 17. FIH adopts similar conformations when complexed to the different substrates. Color code: FIH: grey; carbon-backbone of 3-methyl-2OG (**1**): yellow; Zn: lavender blue; carbon-backbone of the tankyrase-2 fragment peptide (TANK2$_{691-710}$): purple; oxygen: red; nitrogen: blue.

(**a**) Superimposition of a view from the FIH:**1**:TANK2$_{691-710}$ structure with one from the FIH:**1**:CA$_{1-20}$ structure (FIH: light pink, carbon-backbone of 2OG: pink, CA$_{1-20}$ substrate peptide: orange; Supplementary Figure 13) reveals similar FIH conformations (Cα RMSD = 0.10 Å). Both substrate peptides bind FIH in a similar manner (Cα RMSD = 0.57 Å, calculated using the 16 Cα atoms of both Glu693 – Gln708 of the TANK2$_{691-710}$ peptide and Glu3 – Gln18 of the CA$_{1-20}$ peptide); (**b**) superimposition of a view from the FIH:**1**:TANK2$_{691-710}$ structure with one from the FIH:**1**:CA$_{1-20}$ structure reveals near identical conformations of the sidechains of FIH active site residues; (**c**) superimposition of a view from the FIH:**1**:TANK2$_{691-710}$ structure with one from the reported FIH:2OG:TANK2$_{538-558}$ structure (FIH: pale green; carbon-backbone of 2OG: green; tankyrase-2 fragment peptide (538–558): pale orange, Fe: orange; PDB ID: 2Y0I)[10] reveals similar FIH conformations (Cα RMSD = 0.22 Å); (**d**) superimposition of a view from the FIH:**1**:TANK2$_{691-710}$ structure with one from the reported FIH:2OG:TANK2$_{538-558}$ structure reveals nearly identical conformations of the sidechains of FIH active site residues. The conformations of (*S*)-**1** and 2OG are identical except at the C4 carbons. Both TNKS2 substrate peptides bind FIH in a similar manner (Cα RMSD = 0.30 Å, calculated using the 14 Cα atoms of both Glu693 – Asn706 of the TANK2$_{691-710}$ peptide and Ser540 – His553 of the TANK2$_{538-558}$ peptide).

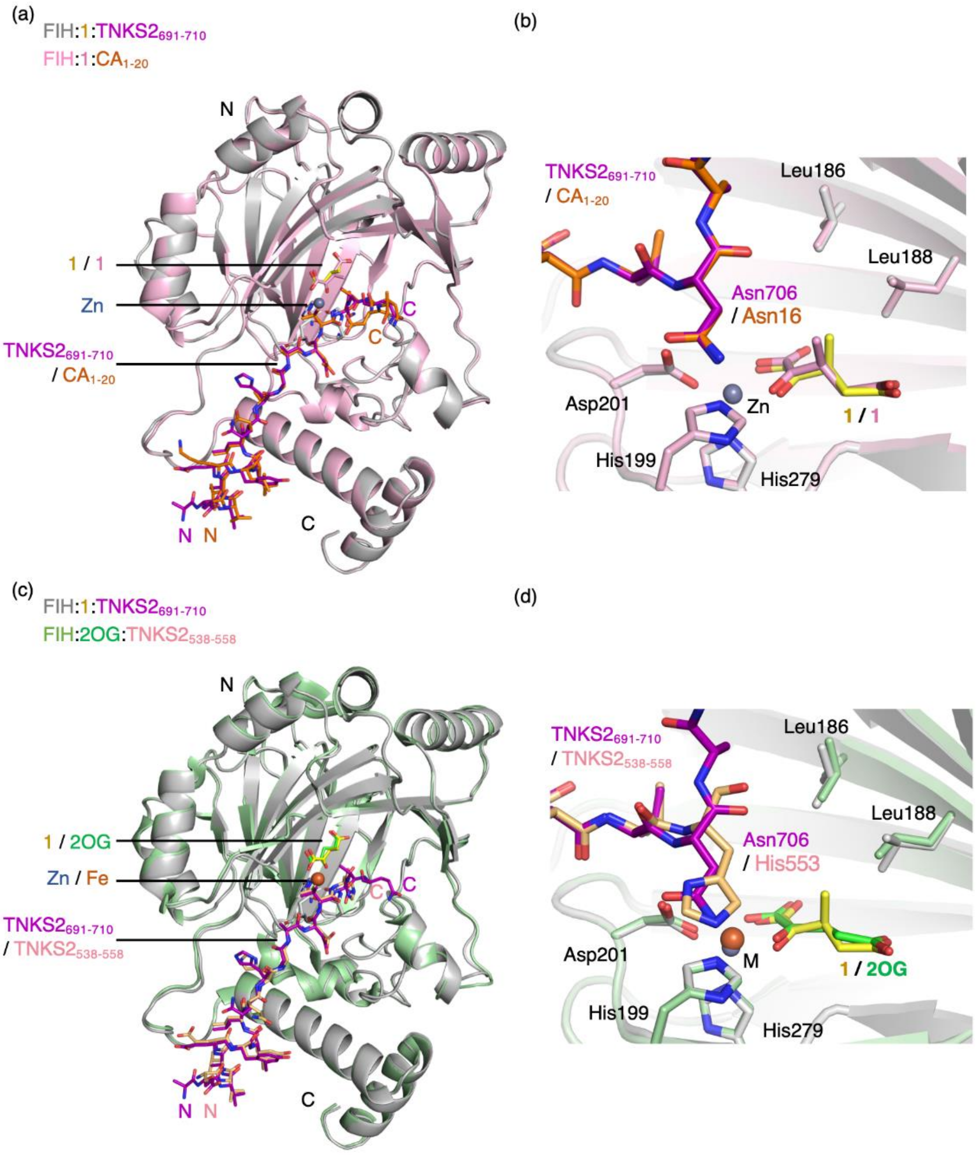

Supplementary Figure 18. Views from a structure of FIH complexed with 4-ethyl-2OG (14), Zn, and the synthetic ankyrin peptide CA_{1-20} (FIH:14:CA_{1-20}; PDB ID: 7A1O). Color code: FIH: grey; carbon-backbone of 4-ethyl-2OG (**14**): blue; Zn: lavender blue; carbon-backbone of the synthetic ankyrin peptide (CA_{1-20}): orange; oxygen: red; nitrogen: blue.

(**a**) Overview of the FIH:**14**:CA_{1-20} structure; (**b**) representative OMIT electron density map (mF_o-DF_c) contoured to 3σ around (*S*)-**14** of the FIH:**14**:CA_{1-20} structure. (*S*)-**14** coordinates to the Zn ion in a bidentate manner and is positioned to interact with the sidechains of FIH active site residues Lys214, Tyr145, Thr196, Asn294, and Asn205 (distances in Å); (**c**) representative OMIT electron density map (mF_o-DF_c) contoured to 2σ around the synthetic ankyrin peptide CA_{1-20} of the FIH:**14**:CA_{1-20} structure; (**d**) superimposition of a view from the FIH:**14**:CA_{1-20} structure with one from the reported FIH:2OG:HIF-1$\alpha_{786-826}$ structure (FIH: pale green, carbon-backbone of 2OG: green, carbon-backbone of the HIF-1$\alpha_{786-826}$ substrate peptide: cyan, Fe: orange; PDB ID: 1H2L)[9] reveals similar FIH conformations (Cα RMSD = 0.17 Å). Note, that the conformations of the N-termini of both substrate peptides are similar, whereas the conformations of the C-termini substantially differ (Cα RMSD = 0.49 Å, calculated using the 10 Cα atoms of both Leu8 – Ala17 of the CA_{1-20} peptide and Leu795 – Ala804 of the HIF-1$\alpha_{786-826}$ peptide).

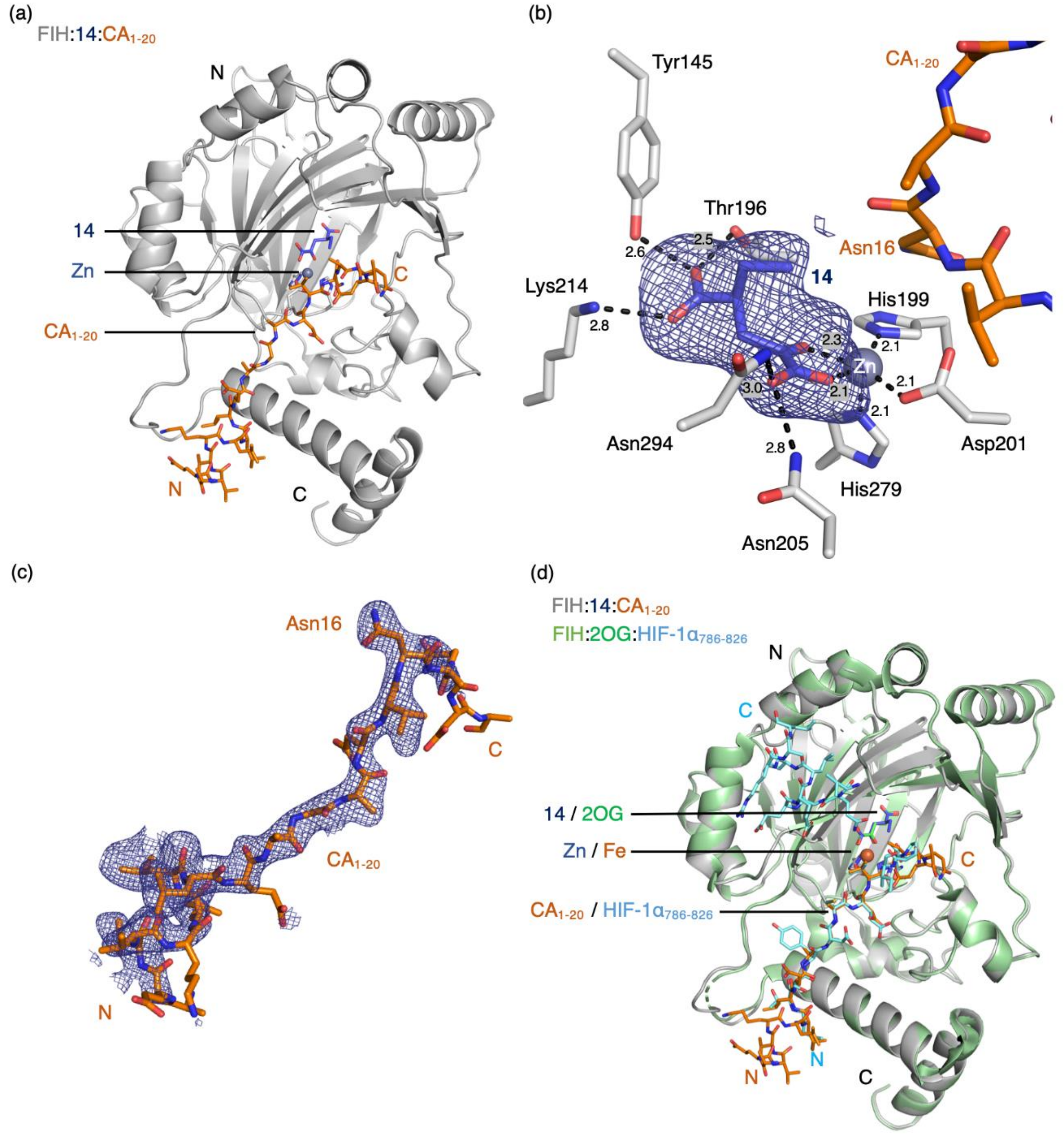

Supplementary Figure 19. Analysis of electron density maps supports the predominant presence of the (*S*)-enantiomer of 4-ethyl-2OG (14) in the FIH:14:$CA_{1\text{-}20}$ structure (PDB ID: 7A1O). Color code: FIH: grey; carbon-backbone of (*S*)-4-ethyl-2OG ((*S*)-**14**): blue; carbon-backbone of (*R*)-4-ethyl-2OG ((*R*)-**14**): deep green; Zn: lavender blue; water: red sphere; oxygen: red; nitrogen: blue.

(**a**) Representative OMIT electron density map (mF_o-DF_c) contoured to 3σ around (*S*)-**14** modelled in the FIH:(*S*)-**14**:$CA_{1\text{-}20}$ structure (blue mesh). Electron density maps (F_o-F_c) contoured to 2σ and -2σ around (*S*)-**14** are shown in green and red mesh, respectively; (**b**) superimposition of (*S*)-**14** and (*R*)-**14** modelled in the FIH:**14**:$CA_{1\text{-}20}$ structure. Representative OMIT electron density map (mF_o-DF_c) contoured to 3σ around (*R*)-**14** modelled in the FIH:(*R*)-**14**:$CA_{1\text{-}20}$ structure (blue mesh). Electron density maps (F_o-F_c) contoured to 2σ and -2σ around (*R*)-**14** are shown in green and red mesh, respectively. Analysis of the van der Waals radii of (*R*)-**14** showed overlap with Leu188 (~0.5 Å), Phe207 (~0.6 Å), and Ile281 (~0.7 Å), whereas no steric clash (more than 0.4 Å overlap) was observed for (*S*)-**14** modelled in the structure. Negative density was calculated around the C3 atom of (*R*)-**14**; negative density at this position was not observed at the -2σ level with (*S*)-**14** as shown in panel (**a**). The superimposed image of shows that the C3 atom of (*S*)-**14** is positioned to avoid the negative densities observed for (*R*)-**14** in the putative FIH:(*R*)-**14**:$CA_{1\text{-}20}$ structure. Thus, the electron density analysis indicates that the (*S*)-enantiomer of **14** is predominantly (at least) present in the FIH:**14**:$CA_{1\text{-}20}$ structure. This proposal is consistent with the binding mode of *N*-(carboxycarbonyl)-D-phenylalanine (NOFD) to FIH[5]; Note, that the nitrogen atom of NOFD affects the Cahn-Ingold-Prelog priority rules resulting in its formal assignment as (*R*)-enantiomer.

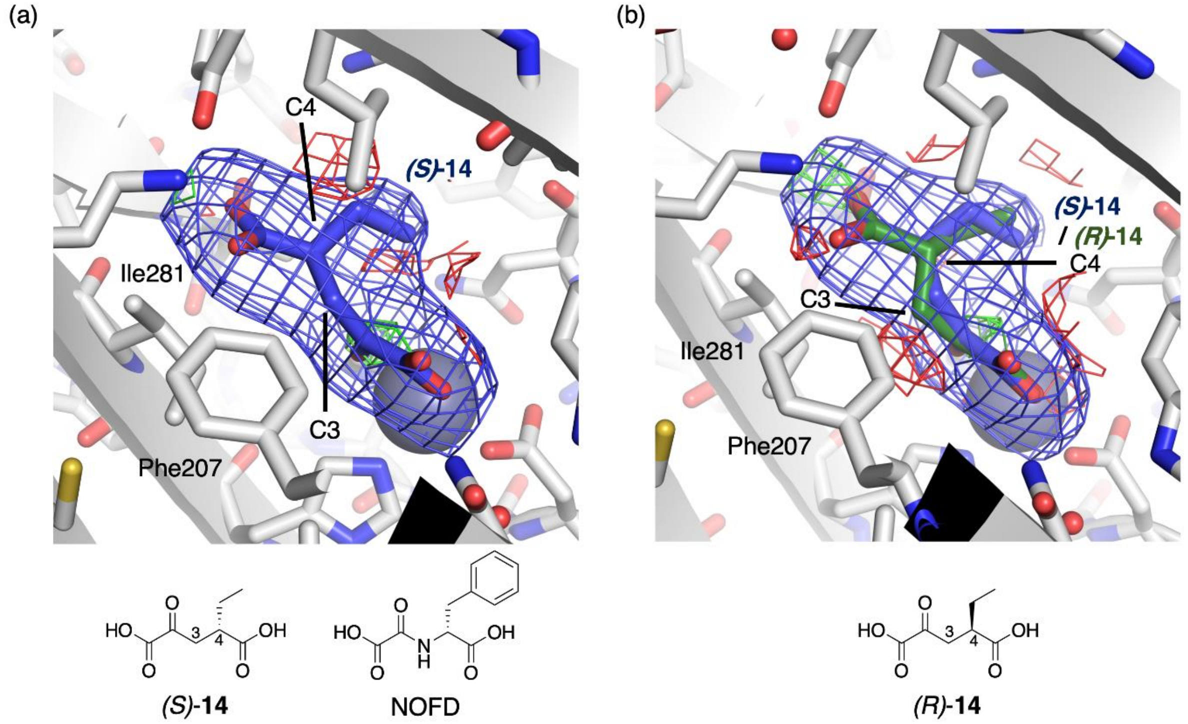

Supplementary Figure 20. Views from a structure of FIH complexed with 4-propyl-2OG (15), Zn, and the synthetic ankyrin peptide CA$_{1-20}$ (FIH:15:CA$_{1-20}$; PDB ID: 7A1P). Color code: FIH: grey; carbon-backbone of 4-ethyl-2OG (**15**): blue; Zn: lavender blue; carbon-backbone of the synthetic ankyrin peptide CA$_{1-20}$: orange; oxygen: red; nitrogen: blue.

(**a**) Overview of the FIH:**15**:CA$_{1-20}$ structure; (**b**) representative OMIT electron density map (mF$_o$-DF$_c$) contoured to 3σ around (*S*)-**15** of the FIH:**15**:CA$_{1-20}$ structure. (*S*)-**15** coordinates to the Zn ion in a bidentate manner and is positioned to interact with the sidechains of FIH active site residues Lys214, Tyr145, Thr196, Asn294, and Asn205 (distances in Å); (**c**) representative OMIT electron density map (mF$_o$−DF$_c$) contoured to 3σ around the synthetic ankyrin peptide CA$_{1-20}$ of the FIH:**15**:CA$_{1-20}$ structure; (**d**) superimposition of a view from the FIH:**15**:CA$_{1-20}$ structure with one from the reported FIH:2OG:HIF-1α$_{786-826}$ structure (FIH: pale green, carbon-backbone of 2OG: green, carbon-backbone of the HIF-1α$_{786-826}$ substrate peptide: cyan, Fe: orange; PDB ID: 1H2L)[9] reveals similar FIH conformations (Cα RMSD = 0.14 Å). Note, that the conformations of the N-termini of both substrate peptides are similar, whereas the conformations of the C-termini substantially differ (Cα RMSD = 0.51 Å, calculated using the 10 Cα atoms of both Leu8 – Ala17 of the CA$_{1-20}$ peptide and Leu795 – Ala804 of the HIF-1α$_{786-826}$ peptide).

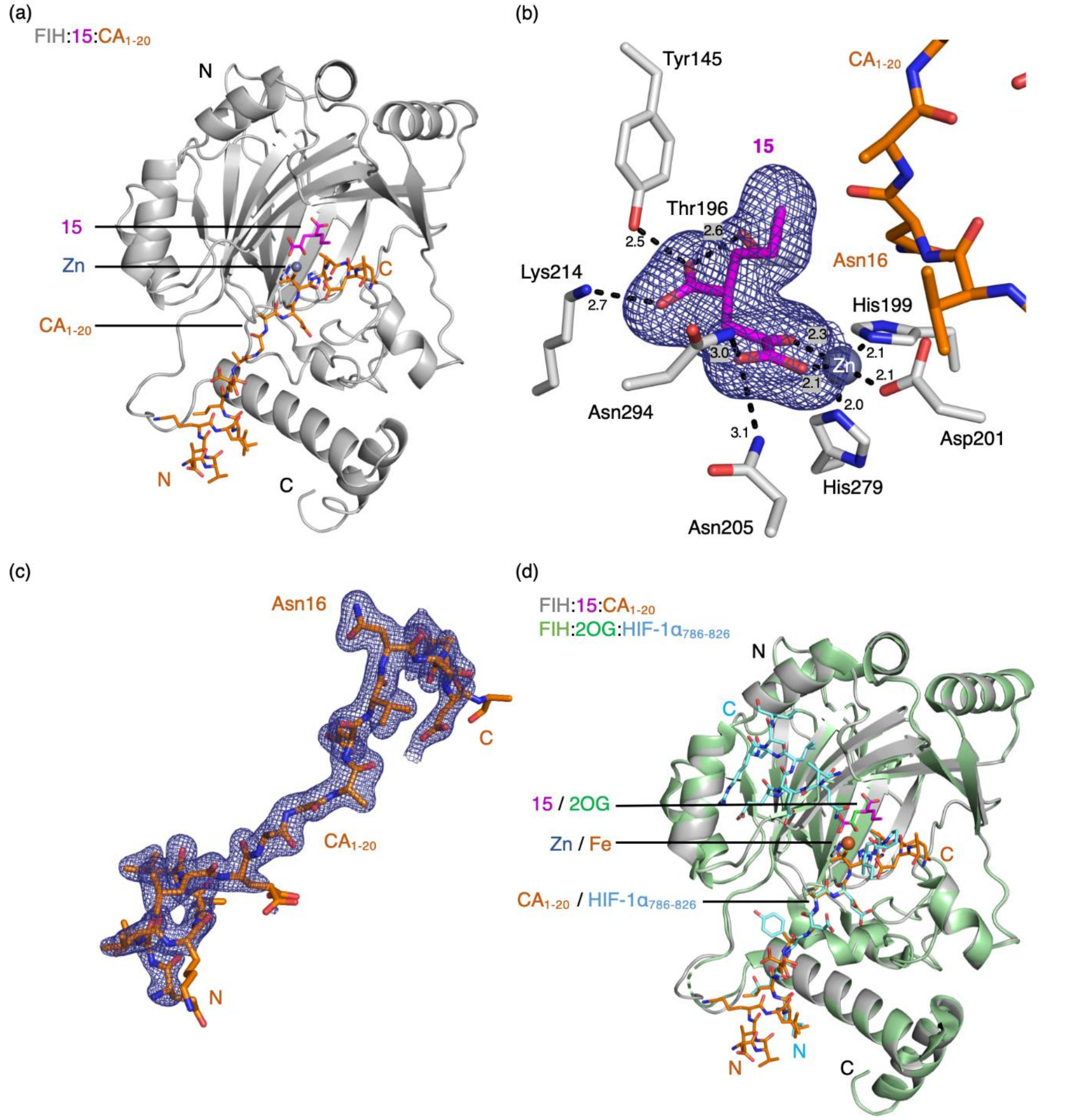

Supplementary Figure 21. Analysis of electron density maps supports the predominant presence of the (*S*)-enantiomer of 4-propyl-2OG (15) in the FIH:15:CA_{1-20} structure (PDB ID: 7A1P). Color code: FIH: grey; carbon-backbone of (*S*)-4-propyl-2OG ((*S*)-**15**): magenta; carbon-backbone of (*R*)-4-propyl-2OG ((*R*)-**15**): deep green; Zn: lavender blue; water: red sphere; oxygen: red; nitrogen: blue.

(**a**) Representative OMIT electron density map (mF_o-DF_c) contoured to 3σ around (*S*)-**15** modelled in the FIH:(*S*)-**15**:CA_{1-20} structure (blue mesh). Electron density maps (F_o-F_c) contoured to 2σ and -2σ around (*S*)-**15** are shown in green and red mesh, respectively; (**b**) superimposition of (*S*)-**15** and (*R*)-**15** modelled in the FIH:**15**:CA_{1-20} structure. Representative OMIT electron density map (mF_o-DF_c) contoured to 3σ around (*R*)-**15** modelled in the FIH:(*R*)-**15**:CA_{1-20} structure (blue mesh). Electron density maps (F_o-F_c) contoured to 2σ and -2σ around (*R*)-**15** are shown in green and red mesh, respectively. Both positive and negative densities were observed around the C4 and C6 atoms of (*R*)-**15**; these positive and negative densities were not observed at the 2σ and -2σ level for (*S*)-**15** as shown in panel (**a**). The superimposed image shows that the C4 and C6 atoms of (*S*)-**15** are positioned to compensate the negative densities observed for (*R*)-**15** in the putative FIH:(*R*)-**15**:CA_{1-20} structure. Thus, the electron density analysis indicates that the (*S*)-enantiomer of **15** is predominantly (at least) present in the FIH:**15**:CA_{1-20} structure. This proposal is consistent with the binding mode of *N*-(carboxycarbonyl)-D-phenylalanine (NOFD) to FIH[5]; Note, that the nitrogen atom of NOFD affects the Cahn-Ingold-Prelog priority rules resulting in its formal assignment as (*R*)-enantiomer.

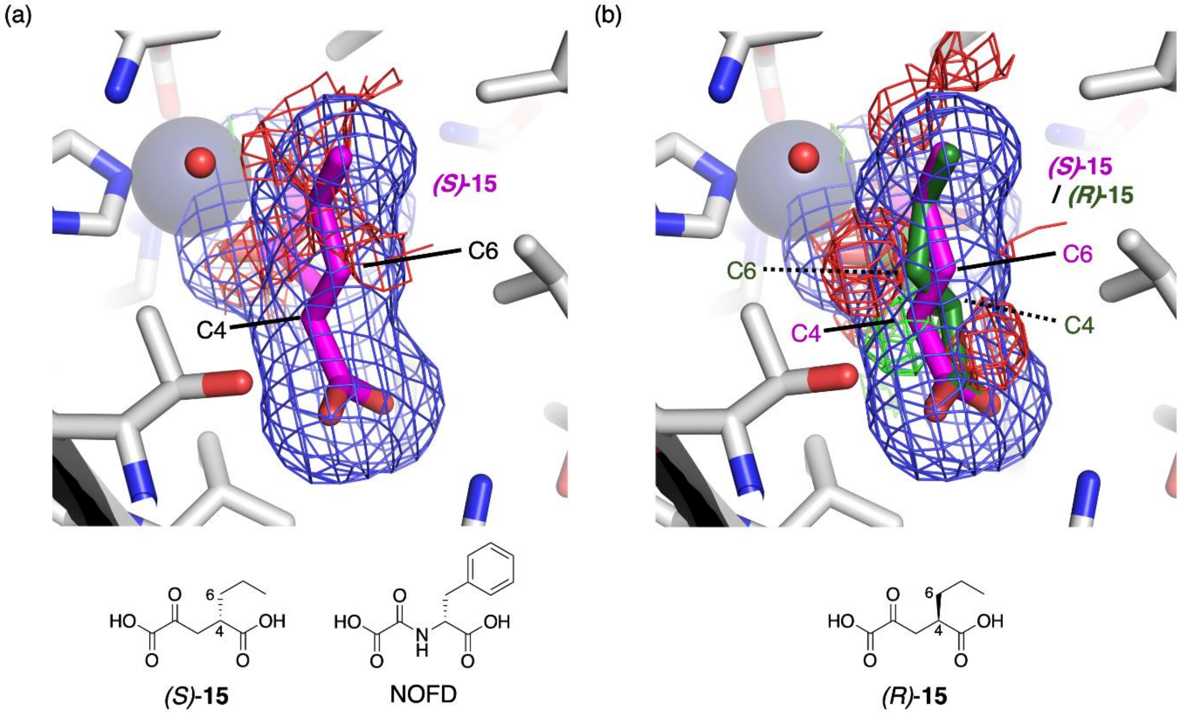

Supplementary Figure 22. Views from a structure of FIH complexed with 3-(carboxycarbonyl)-cyclopentane-1-carboxylic acid (22), Zn, and the synthetic ankyrin peptide CA_{1-20} (FIH:22:CA_{1-20}; PDB ID: 7A1Q). Color code: FIH: grey; carbon-backbone of 3-(carboxycarbonyl)cyclopentane-1-carboxylic acid (**22**): sky blue; Zn: lavender blue; carbon-backbone of the synthetic ankyrin peptide CA_{1-20}: orange; oxygen: red; nitrogen: blue.

(**a**) Overview of the FIH:**22**:CA_{1-20} structure; (**b**) representative OMIT electron density map (mF_o-DF_c) contoured to 5σ around (1*R*,3*S*)-**22** of the FIH:**22**:CA_{1-20} structure. (1*R*,3*S*)-**22** coordinates to the Zn ion in a bidentate manner and is positioned to interact with the sidechains of FIH active site residues Lys214, Tyr145, Thr196, Asn294, and Asn205 (distances in Å); (**c**) representative OMIT electron density map (mF_o−DF_c) contoured to 2σ around the synthetic ankyrin peptide CA_{1-20} of the FIH:**22**:CA_{1-20} structure; (**d**) superimposition of a view from the FIH:**22**:CA_{1-20} structure with one from the reported FIH:2OG:HIF-1$\alpha_{786-826}$ structure (FIH: pale green, carbon-backbone of 2OG: green, carbon-backbone of the HIF-1$\alpha_{786-826}$ substrate peptide: cyan, Fe: orange; PDB ID: 1H2L)[9] reveals similar FIH conformations (Cα RMSD = 0.14 Å). Note, that the conformations of the N-termini of both substrate peptides are similar, whereas the conformations of the C-termini substantially differ (Cα RMSD = 0.50 Å, calculated using the 10 Cα atoms of both Leu8 – Ala17 of the CA_{1-20} peptide and Leu795 – Ala804 of the HIF-1$\alpha_{786-826}$ peptide).

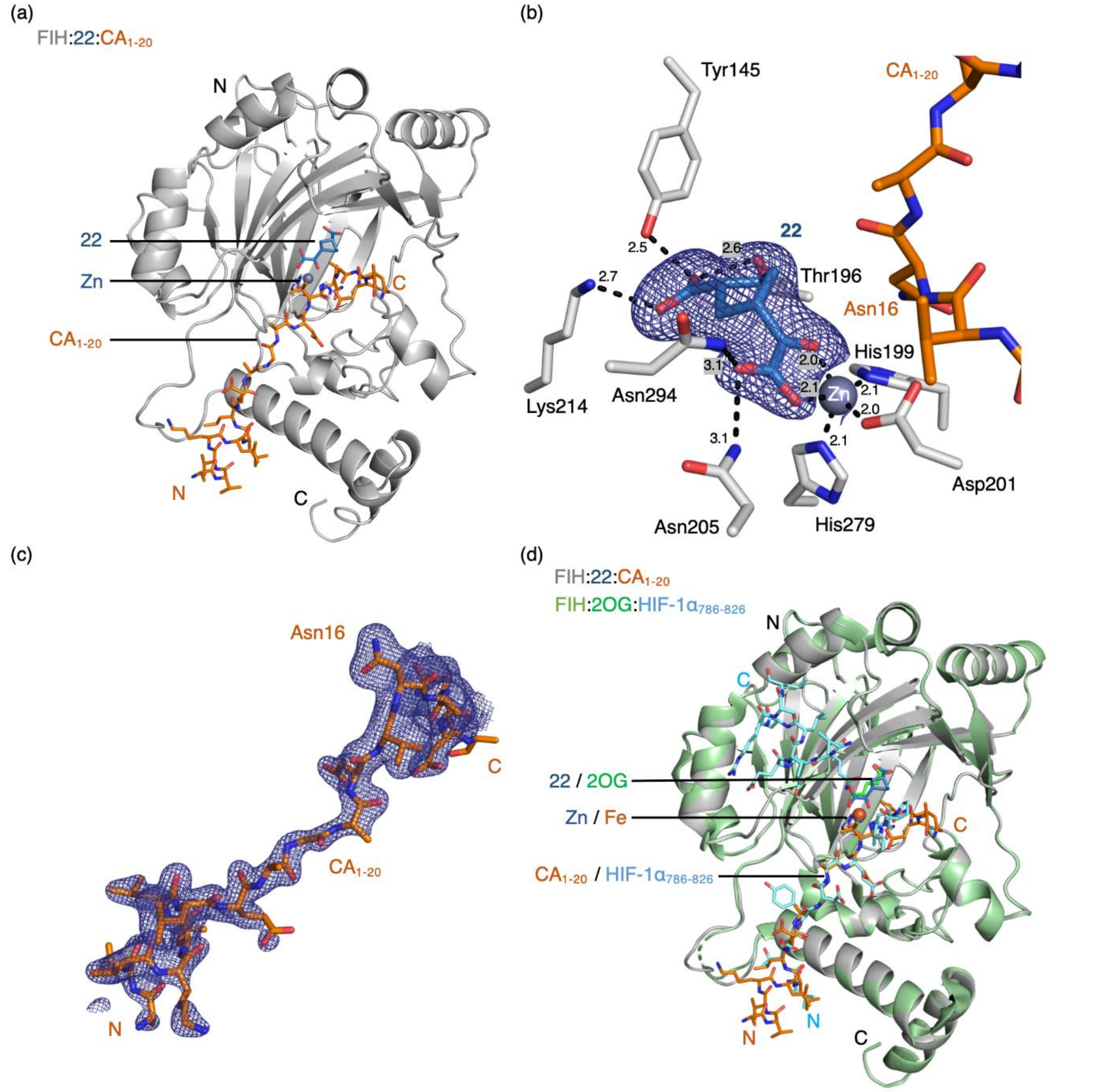

Supplementary Figure 23. Analysis of electron density maps supports the predominant presence of the (1*R*,3*S*)-enantiomer of 3-(carboxycarbonyl)cyclopentane-1-carboxylic acid (22) in the FIH:22:CA$_{1\text{-}20}$ structure (PDB ID: 7A1Q). Color code: FIH: grey; carbon-backbone of (1*R*,3*S*)-3-(carboxycarbonyl)cyclopentane-1-carboxylic acid ((1*R*,3*S*)-**22**): sky blue; carbon-backbone of (1*R*,3*R*)-3-(carboxycarbonyl)cyclopentane-1-carboxylic acid ((1*R*,3*R*)-**22**): deep green; Zn: lavender blue; water: red sphere; oxygen: red; nitrogen: blue.

(**a**) Representative OMIT electron density map ($mF_o\text{-}DF_c$) contoured to 3σ around (1*R*,3*S*)-**22** modelled in the FIH:(1*R*,3*S*)-**22**:CA$_{1\text{-}20}$ structure (blue mesh). Electron density maps ($F_o\text{-}F_c$) contoured to 2σ and -2σ around (1*R*,3*S*)-**22** are shown in green and red mesh, respectively; (**b**) superimposition of (1*R*,3*S*)-**22** and (1*R*,3*R*)-**22** modelled in the FIH:**22**:CA$_{1\text{-}20}$ structure. Representative OMIT electron density map ($mF_o\text{-}DF_c$) contoured to 3σ around (1*R*,3*R*)-**22** modelled in the FIH:(1*R*,3*R*)-**22**:CA$_{1\text{-}20}$ structure (blue mesh). Electron density maps ($F_o\text{-}F_c$) contoured to 2σ and -2σ around (1*R*,3*R*)-**22** are shown in green and red mesh, respectively. Both positive and negative densities were observed around the C3 atom of (1*R*,3*R*)-**22**; these positive and negative densities were not observed at the 2σ and -2σ level for (1*R*,3*S*)-**22** as shown in panel (**a**). The superimposed image of (1*R*,3*S*)-**22** shows that the C3 atom of (1*R*,3*S*)-**22** is positioned to compensate the positive and negative densities observed for (1*R*,3*R*)-**22**. Thus, the electron density analysis indicates that the (1*R*,3*S*)-enantiomer of **22** is predominantly (at least) present in the FIH:**22**:CA$_{1\text{-}20}$ structure.

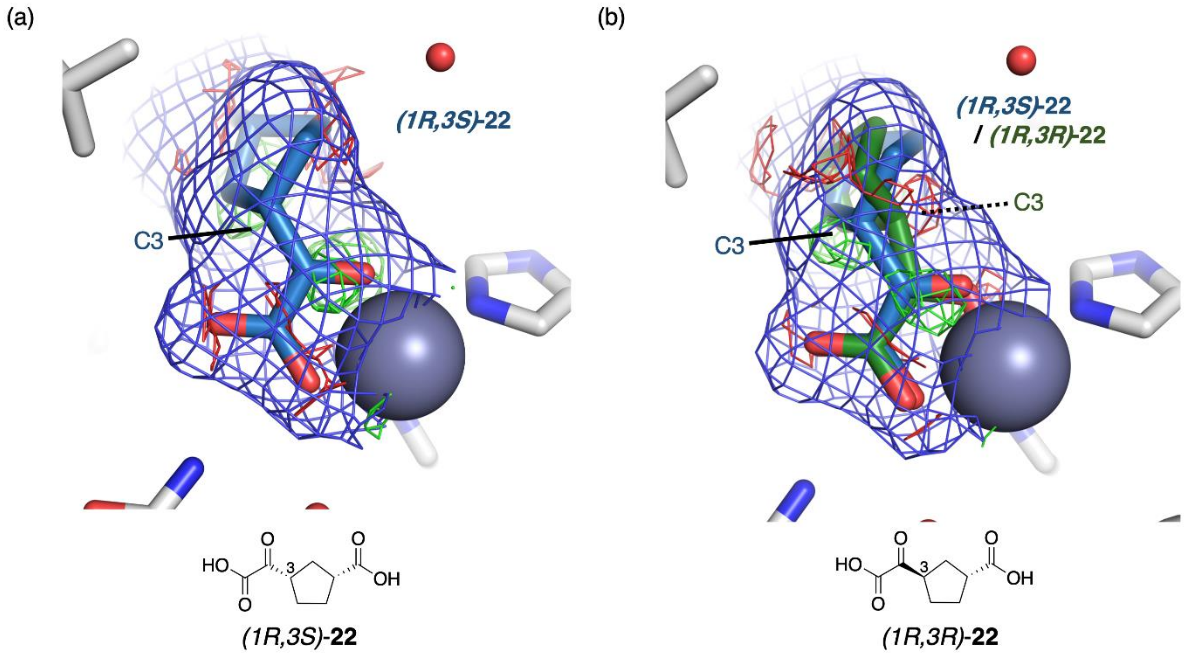

Supplementary Figure 24. Superimposition of the reported FIH:2OG:HIF-1α$_{786\text{-}826}$ structure with the reported AspH:2OG:hFX-EGFD1$_{86\text{-}826}$-4Ser structure. Color code: FIH: grey; carbon-backbone of 2-oxoglutarate (2OG): green; Fe: orange; carbon-backbone of the HIF-1α$_{786\text{-}826}$ substrate peptide: cyan; oxygen: red; nitrogen: blue.

(**a**) The superimposition of a view from the reported FIH:2OG:HIF-1α$_{786\text{-}826}$ structure (PDB ID: 1H2L)[9] with one from the reported AspH:2OG:hFX-EGFD1$_{86\text{-}826}$-4Ser structure (AspH: light pink, carbon-backbone of 2OG: deep pink, carbon-backbone of the hFX-EGFD1$_{86\text{-}826}$-4Ser substrate peptide: magenta; Mn: violet; PDB ID: 6YYW)[1] reveals little structural similarity of the two 2OG oxygenases except for their conserved double-stranded β-helix folds bearing the active site (Cα RMSD = 1.27 Å; calculated using 48 Cα atoms which form the double-stranded β-helix fold); (**b**) superimposition of a view from the active site of FIH:2OG:HIF-1α$_{786\text{-}826}$ structure with one from the reported AspH:2OG:hFX-EGFD1$_{86\text{-}826}$-4Ser structure reveals that 2OG occupies different conformations in both structures, especially with regard to its C5 carboxylates.

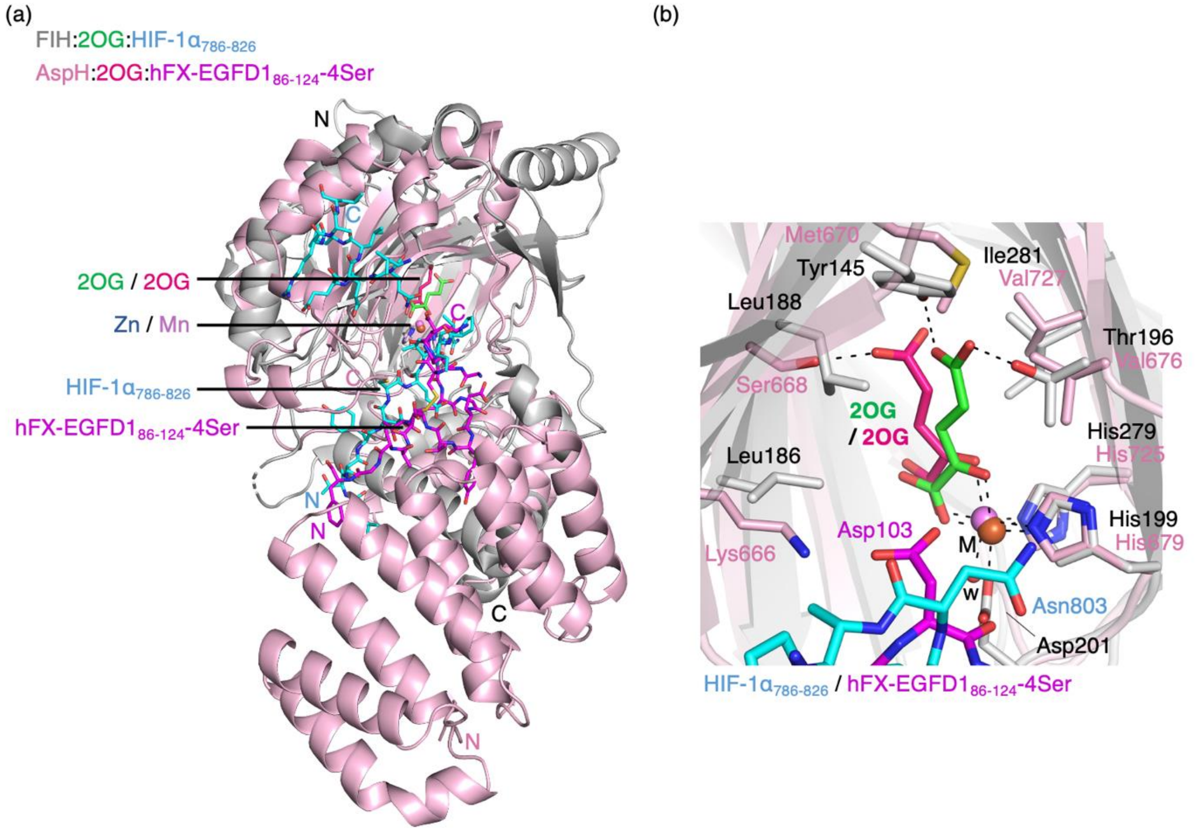

Supplementary Figure 25. Superimposition of the FIH:1:CA$_{1-20}$ structure with the reported AspH:1:hFX-EGFD1$_{86-826}$-4Ser structure. Color code: FIH: grey; carbon-backbone of 3-methyl-2OG (**1**): yellow; Zn: lavender blue; carbon-backbone of the synthetic ankyrin peptide CA$_{1-20}$: orange; oxygen: red; nitrogen: blue.

The superimposition of a view from the FIH:**1**:CA$_{1-20}$ structure with one from the reported AspH:**1**:hFX-EGFD1$_{86-826}$-4Ser structure (AspH: light pink, carbon-backbone of **1**: deep pink, carbon-backbone of the hFX-EGFD1$_{86-826}$-4Ser substrate peptide: magenta; Mn: violet; PDB ID: 6YYX)[1] reveals little structural similarity of the two 2OG oxygenases except for their conserved double-stranded β-helix folds bearing the active site (Cα RMSD = 1.30 Å; calculated using 58 Cα atoms which form the double-stranded β-helix fold).

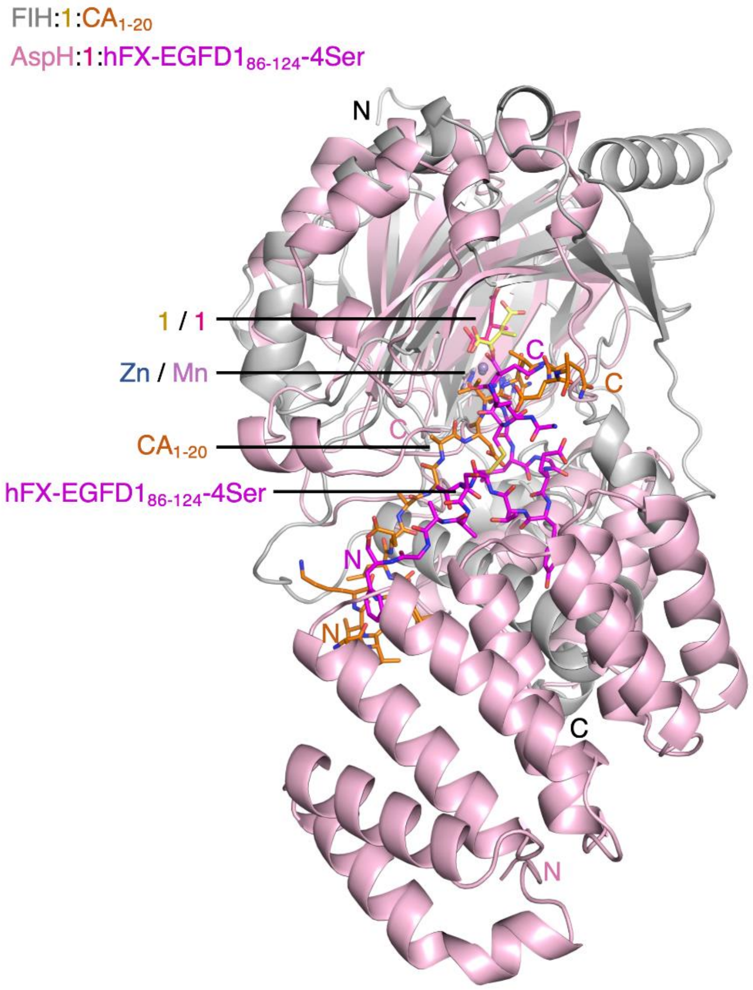

Supplementary Figure 26. Robustness of the FIH inhibition assays. (**a**) Z'-factors[11] (circles) and (**b**) signal-to-noise ratios (S/N, squares) for all FIH inhibition assay plates analyzed to determine IC_{50}-values. The Z'-factors >0.5 (grey line) indicate a stable and robust assay[11]. 16 Compounds were investigated per plate including DMSO and NOFD[5] which were used as negative and positive inhibition controls, respectively; technical duplicates were in adjacent wells. Z'-factors and S/N-ratios were determined according to the cited literature using Microsoft Excel.

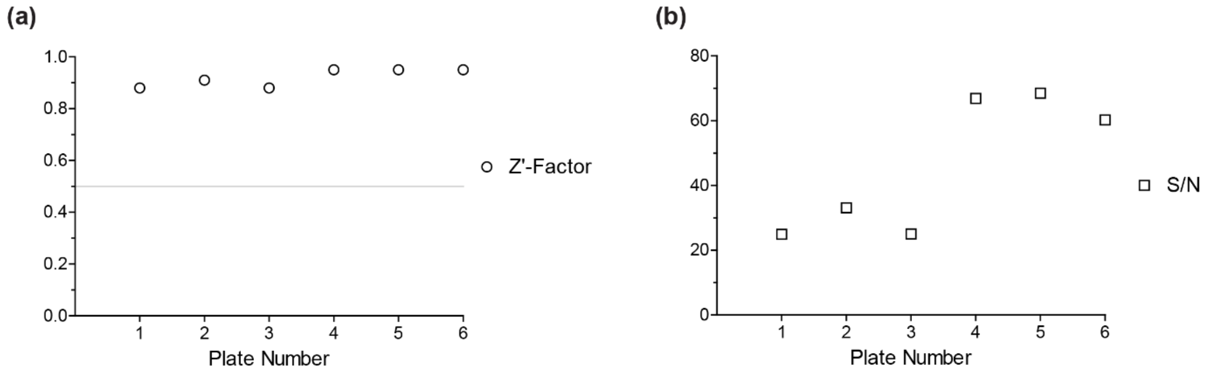

Supplementary Figure 27. 4-Ethyl-2OG (14) selectively inhibits AspH in the presence of FIH in a dose-dependent manner. FIH and AspH-catalyzed peptide hydroxylations were analyzed simultaneously using SPE-MS as described in the Methods section; competition experiments were performed in the same reaction vessel using equimolar 2OG oxygenase and substrate concentrations. Conditions: 0.15 µM FIH, 5.0 µM $CA_{1\text{-}20}$ peptide (FIH substrate)[3], 0.15 µM AspH, 5.0 µM hFX-$EGFD1_{86\text{-}124}$-4Ser peptide (AspH substrate)[8], 100 µM L-ascorbic acid (LAA), 10 µM ammonium iron(II) sulfate hexahydrate (FAS, $(NH_4)_2Fe(SO_4)_2{\cdot}6H_2O$), and 2OG and/or 4-ethyl-2OG (**14**) in buffer (50 mM Tris, 50 mM NaCl, pH 7.5, 20° C). Measurement times were normalized to the first sample injection analyzed after the addition of the 2OG oxygenases to the Substrate Mixture (t = 0 s), by which time low levels of hydroxylation were manifest. **14** is a reported inhibitor of human AspH (Supplementary Table 1)[1], and a cosubstrate for FIH (Table 1).

Varying the concentration of **14** in the presence of 100 µM 2OG reveals that **14** selectively inhibits AspH (black circles) in a dose dependent manner, while no substantial inhibitory effect on FIH activity (orange squares) was observed. AspH catalysis was entirely suppressed by a tenfold excess of **14** in the investigated timescale. In the absence of 2OG, **14** selectively sustains FIH catalysis (panel **f**); however, compared to the natural cosubstrate 2OG (panel **a**), **14** is a less efficient cosubstrate – the time taken to reach a similar substrate conversion as with 2OG is about four times longer with **14**. Cosubstrate concentrations: (**a**) 100 µM 2OG, 0 µM **14**; (**b**) 100 µM 2OG, 100 µM **14**; (**c**) 100 µM 2OG, 250 µM **14**; (**d**) 100 µM 2OG, 500 µM **14**; (**e**) 100 µM 2OG, 1000 µM **14**; (**f**) 0 µM 2OG, 100 µM **14**. Source data are provided as a Source Data file.

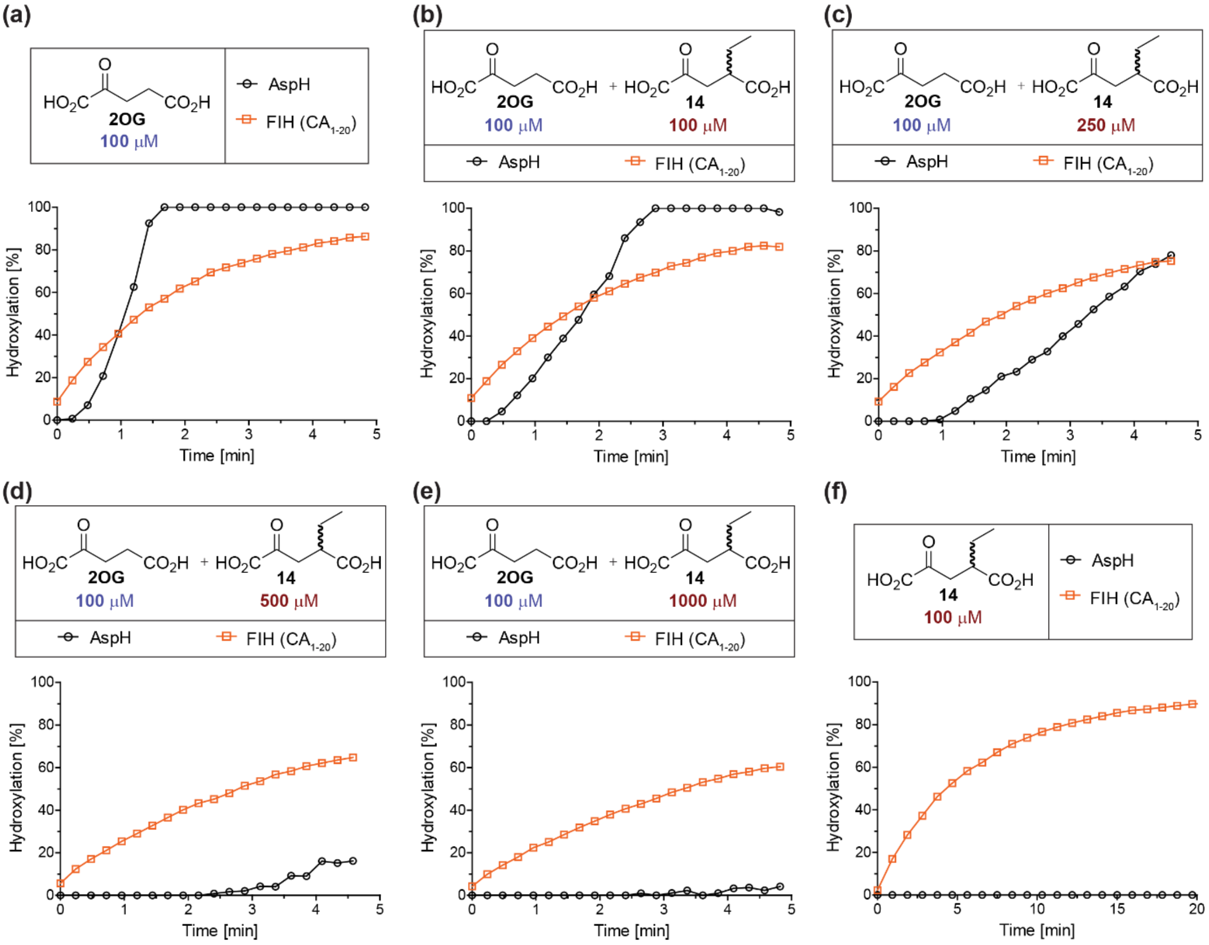

Supplementary Figure 28. Determination of the FIH K_i^{app} for 4-(3-phenylprop-1-yl)-2OG (18) (continues on the following page). Maximum velocities (v_{max}^{app}) and Michaelis constants (K_m^{app}) of FIH for 2OG were determined by monitoring FIH-catalyzed hydroxylation of the HIF-1$\alpha_{788-822}$ peptide[4] at variable 2OG concentrations in the presence of 0, 0.5, 1.0, 2.0 or 4.0 μM 4-(3-phenylprop-1-yl)-2OG (**18**), respectively, using SPE-MS assays as described in the Methods section. Conditions: 0.15 μM FIH, 5.0 μM substrate peptide, 100 μM L-ascorbic acid (LAA), and 20 μM ammonium iron(II) sulfate hexahydrate (FAS, $(NH_4)_2Fe(SO_4)_2 \cdot 6H_2O$) in buffer (50 mM Tris, 50 mM NaCl, pH 7.5, 20° C). Measurement times were normalized to the first sample injection analyzed after the addition of FIH to the Substrate Mixture (t = 0 s), by which time low levels of hydroxylation were manifest. Data are shown as the mean of independent triplicates (n = 3; mean ± standard deviation, SD). Source data are provided as a Source Data file.

(**a**) Time courses of the FIH-catalyzed hydroxylation reactions of HIF-1$\alpha_{788-822}$ at the shown 2OG concentrations, determined in the absence of **18**; (**b**) hydroxylation rates used for the kinetic analysis; (**c**) in the absence of **18**, the FIH v_{max}^{app} and K_m^{app}-values for 2OG are 5.9 ± 0.1·10^{-3} μM·s^{-1} and 0.74 ± 0.07 μM, respectively, as determined by non-linear regression; (**d**) time courses of the FIH-catalyzed hydroxylation reactions of HIF-1$\alpha_{788-822}$ at the shown 2OG concentrations, determined in the presence of 0.5 μM **18**; (**e**) hydroxylation rates used for the kinetic analysis; (**f**) in the presence of 0.5 μM **18**, the FIH v_{max}^{app} and K_m^{app}-values for 2OG are 5.8 ± 0.1·10^{-3} μM·s^{-1} and 2.12 ± 0.20 μM, respectively, as determined by non-linear regression; (**g**) time courses of the FIH-catalyzed hydroxylation reaction of HIF-1$\alpha_{788-822}$ at the shown 2OG concentrations, determined in the presence of 1.0 μM **18**; (**h**) hydroxylation rates used for the kinetic analysis; (**i**) in the presence of 1.0 μM **18**, the FIH v_{max}^{app} and K_m^{app}-values for 2OG are 6.2 ± 0.1·10^{-3} μM·s^{-1} and 4.58 ± 0.34 μM, respectively, as determined by non-linear regression; (**j**) time courses of the FIH-catalyzed hydroxylation reaction of HIF-1$\alpha_{788-822}$ at the shown 2OG concentrations, determined in the presence of 2.0 μM **18**; (**k**) hydroxylation rates used for the kinetic analysis; (**l**) in the presence of 2.0 μM **18**, the FIH v_{max}^{app} and K_m^{app}-values for 2OG are 5.9 ± 0.2·10^{-3} μM·s^{-1} and 9.62 ± 0.91 μM, respectively, as determined by non-linear regression; (**m**) time courses of the FIH-catalyzed hydroxylation reaction of HIF-1$\alpha_{788-822}$ at the shown 2OG concentrations, determined in the presence of 4.0 μM **18**; (**n**) hydroxylation rates used for the kinetic analysis; (**o**) in the presence of 4.0 μM **18**, the FIH v_{max}^{app} and K_m^{app}-values for 2OG are 6.1 ± 0.3·10^{-3} μM·s^{-1} and 25.1 ± 2.5 μM, respectively, as determined by non-linear regression.

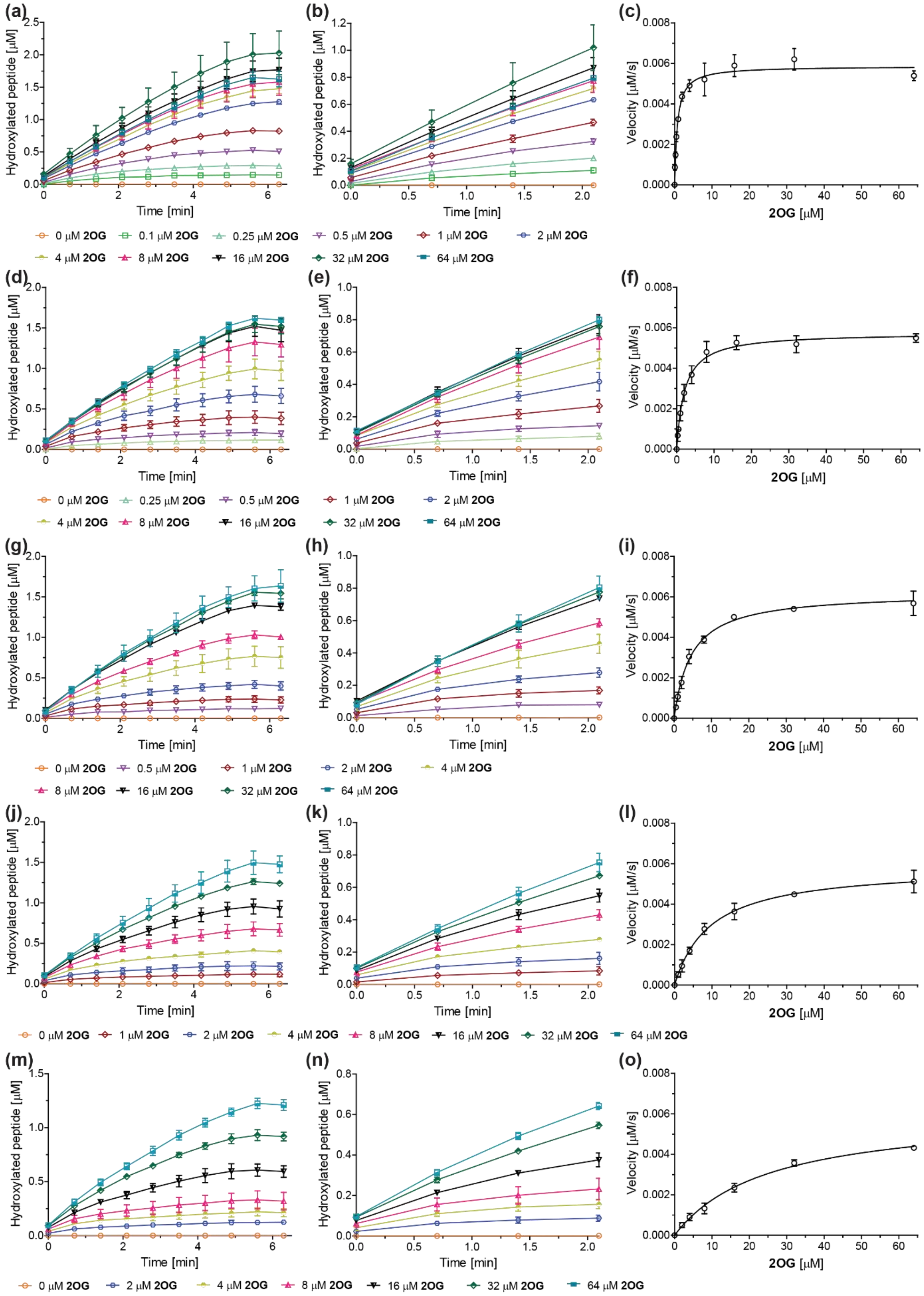

(a)
(b)
(c)
Hydroxylated peptide [µM]
Time [min]
Velocity [µM/s]
2OG [µM]
0 µM 2OG
0.1 µM 2OG
0.25 µM 2OG
0.5 µM 2OG
1 µM 2OG
2 µM 2OG
4 µM 2OG
8 µM 2OG
16 µM 2OG
32 µM 2OG
64 µM 2OG
(d)
(e)
(f)
Hydroxylated peptide [µM]
Time [min]
Velocity [µM/s]
2OG [µM]
0 µM 2OG
0.25 µM 2OG
0.5 µM 2OG
1 µM 2OG
2 µM 2OG
4 µM 2OG
8 µM 2OG
16 µM 2OG
32 µM 2OG
64 µM 2OG
(g)
(h)
(i)
Hydroxylated peptide [µM]
Time [min]
Velocity [µM/s]
2OG [µM]
0 µM 2OG
0.5 µM 2OG
1 µM 2OG
2 µM 2OG
4 µM 2OG
8 µM 2OG
16 µM 2OG
32 µM 2OG
64 µM 2OG
(j)
(k)
(l)
Hydroxylated peptide [µM]
Time [min]
Velocity [µM/s]
2OG [µM]
0 µM 2OG
1 µM 2OG
2 µM 2OG
4 µM 2OG
8 µM 2OG
16 µM 2OG
32 µM 2OG
64 µM 2OG
(m)
(n)
(o)
Hydroxylated peptide [µM]
Time [min]
Velocity [µM/s]
2OG [µM]
0 µM 2OG
2 µM 2OG
4 µM 2OG
8 µM 2OG
16 µM 2OG
32 µM 2OG
64 µM 2OG

Supplementary Figure 29. Views from a crystal structure of FIH complexed with 4-(3-phenylpropyl)-2OG (18) and Zn (FIH:18; PDB ID: 7A1J). Color code: FIH: grey; carbon-backbone of 3-(3-phenylpropyl)-2OG (**18**): salmon; Zn: lavender blue; oxygen: red; nitrogen: blue.

(**a**) Overview of the FIH:**18** crystal structure; (**b**) superimposition of a view from the FIH:**18** structure with one from the reported FIH:2OG:HIF-1α$_{786\text{-}826}$ structure (FIH: pale green, carbon-backbone of 2OG: green, carbon-backbone of the HIF-1α$_{786\text{-}826}$ substrate peptide: cyan, Fe: orange; PDB ID: 1H2L)[9] reveals similar FIH conformations (Cα RMSD = 0.14 Å); (**c**) superimposition of a fold of the FIH:**18** structure with one from the reported FIH:NOFD structure (FIH: purple, carbon-backbone of *N*-(carboxycarbonyl)-D-phenylalanine (NOFD): marine blue, Fe: orange; PDB ID: 1YCI)[5] reveals similar FIH conformations (Cα RMSD = 0.28 Å).

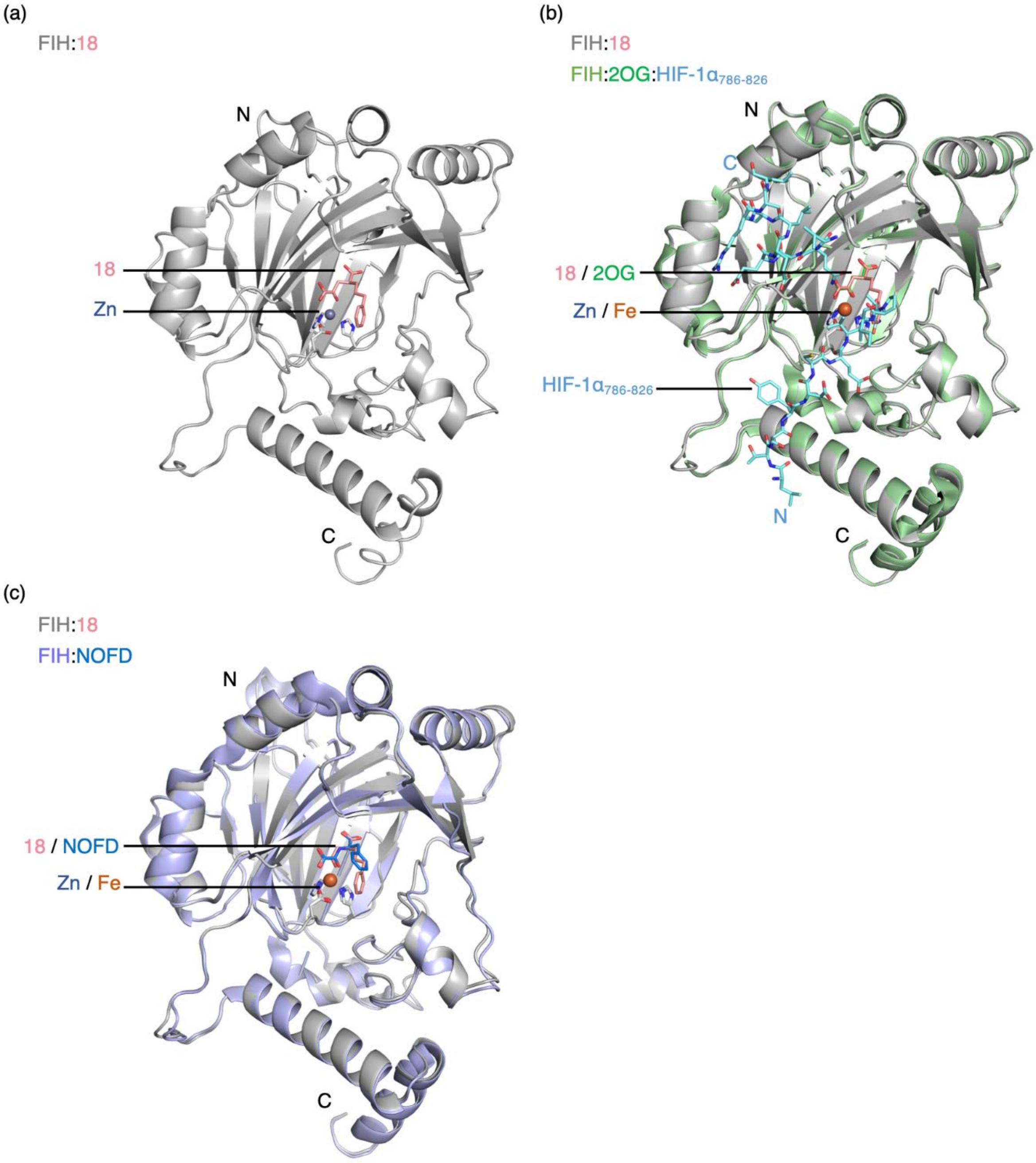

Supplementary Figure 30. Analysis of electron density maps supports the predominant presence of the (*S*)-enantiomer of 4-(3-phenylpropyl)-2OG (18) in the FIH:18 structure (PDB ID: 7A1J). Color code: FIH: grey; carbon-backbone of (*S*)-3-(3-phenylpropyl)-2OG ((*S*)-**18**): salmon; carbon-backbone of (*R*)-3-(3-phenylpropyl)-2OG ((*R*)-**18**): deep green; Zn: lavender blue; water: red sphere; oxygen: red; nitrogen: blue.

(**a**) Representative OMIT electron density map (mF_o-DF_c) contoured to 3σ around (*S*)-**18** modelled in the FIH:**18** structure (blue mesh). Electron density maps (F_o-F_c) contoured to 2σ and -2σ around (*S*)-**18** are shown in green and red mesh, respectively; (**b**) superimposition of (*S*)-**18** and (*R*)-**18** modelled in the FIH:**18** structure. Representative OMIT electron density map (mF_o-DF_c) contoured to 3σ around (*R*)-**18** modelled in the FIH:**18** structure (blue mesh). Electron density maps (F_o-F_c) contoured to 2σ and -2σ around (*R*)-**18** are shown in green and red mesh, respectively. Negative density at the -2σ level was observed around the C4 and C6 atoms of (*R*)-**18** modelled in the FIH:**18** structure; negative density for these atoms of (*S*)-**18** was not observed at the -2σ level as shown in panel (**a**). The superimposed image shows that the C4 and C6 atoms of (*S*)-**18** are positioned to avoid the negative densities observed for (*R*)-**18** in the putative FIH:(*R*)-**18** structure. Thus, the analysis of the electron density maps indicates that the (*S*)-enantiomer of **18** is predominantly (at least) present in the FIH:**18** structure. This proposal is consistent with the binding mode of *N*-(carboxycarbonyl)-D-phenylalanine (NOFD) to FIH[5]; Note, that the nitrogen atom of NOFD affects the Cahn-Ingold-Prelog priority rules resulting in its formal assignment as the (*R*)-enantiomer.

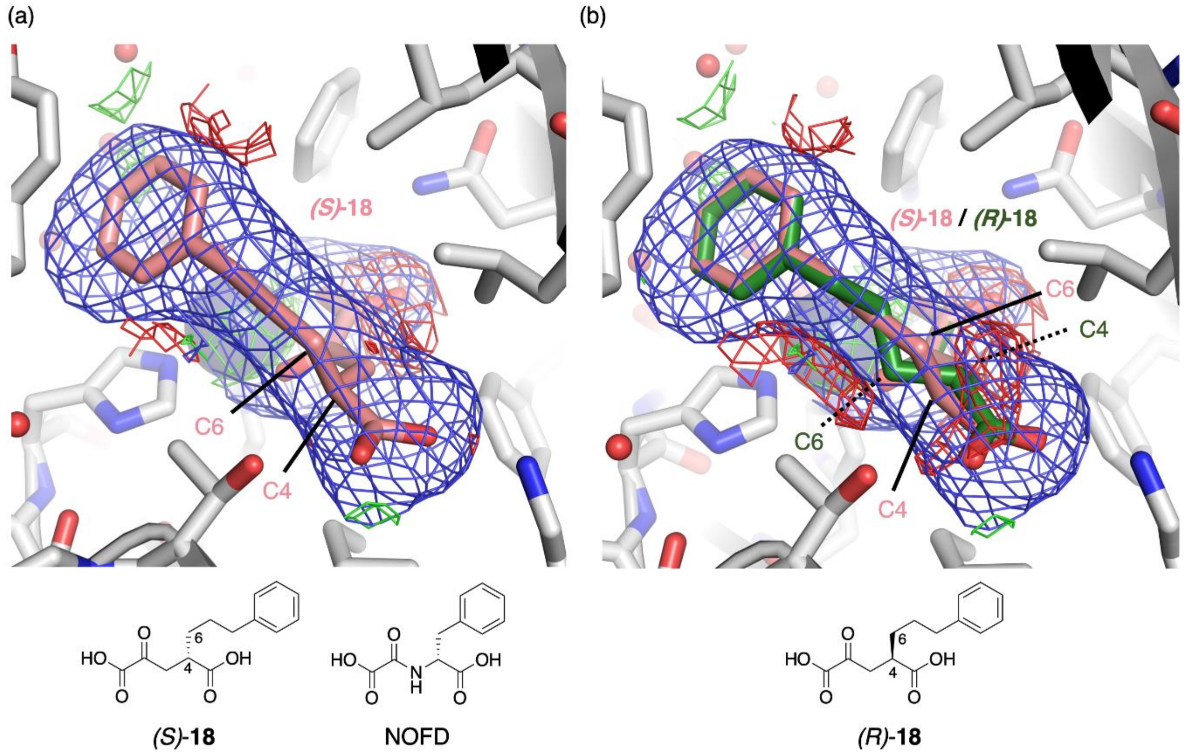

Supplementary Figure 31. Views from a crystal structure of FIH complexed with 3-((9,9-dimethyl-9*H*-fluoren-2-yl)methyl)-2OG (11) and Zn (FIH:11; PDB ID: 7A1K). Color code: FIH: grey; carbon-backbone of 3-((9,9-dimethyl-9*H*-fluoren-2-yl)methyl)-2OG (**11**): violet; Zn: lavender blue; oxygen: red; nitrogen: blue.
(**a**) Overview of the FIH:**11** crystal structure; (**b**) superimposition of a view from the FIH:**11** structure with one from the reported FIH:2OG:HIF-1$\alpha_{786\text{-}826}$ structure (FIH: pale green, carbon-backbone of 2OG: green, carbon-backbone of the HIF-1$\alpha_{786\text{-}826}$ substrate peptide: cyan, Fe: orange; PDB ID: 1H2L)[9] reveals similar FIH conformations (Cα RMSD = 0.17 Å); (**c**) superimposition of a view from the FIH:**11** structure with one from the reported FIH:NOFD structure (FIH: light blue, carbon-backbone of NOFD: marine blue, Fe: orange; PDB ID: 1YCI)[5] reveals similar FIH conformations (Cα RMSD = 0.24 Å); (**d**) superimposition of an active site view from the FIH:**11** structure with one from the reported FIH:NOFD structure (FIH: light blue, carbon-backbone of NOFD: marine blue, Fe: orange; PDB ID: 1YCI)[5] reveal conformational changes in the sidechains of Tyr102$_{FIH}$ and Gln147$_{FIH}$, presumably to avoid unfavorable steric interactions with the bulky fluorenyl-substituent of **11**.

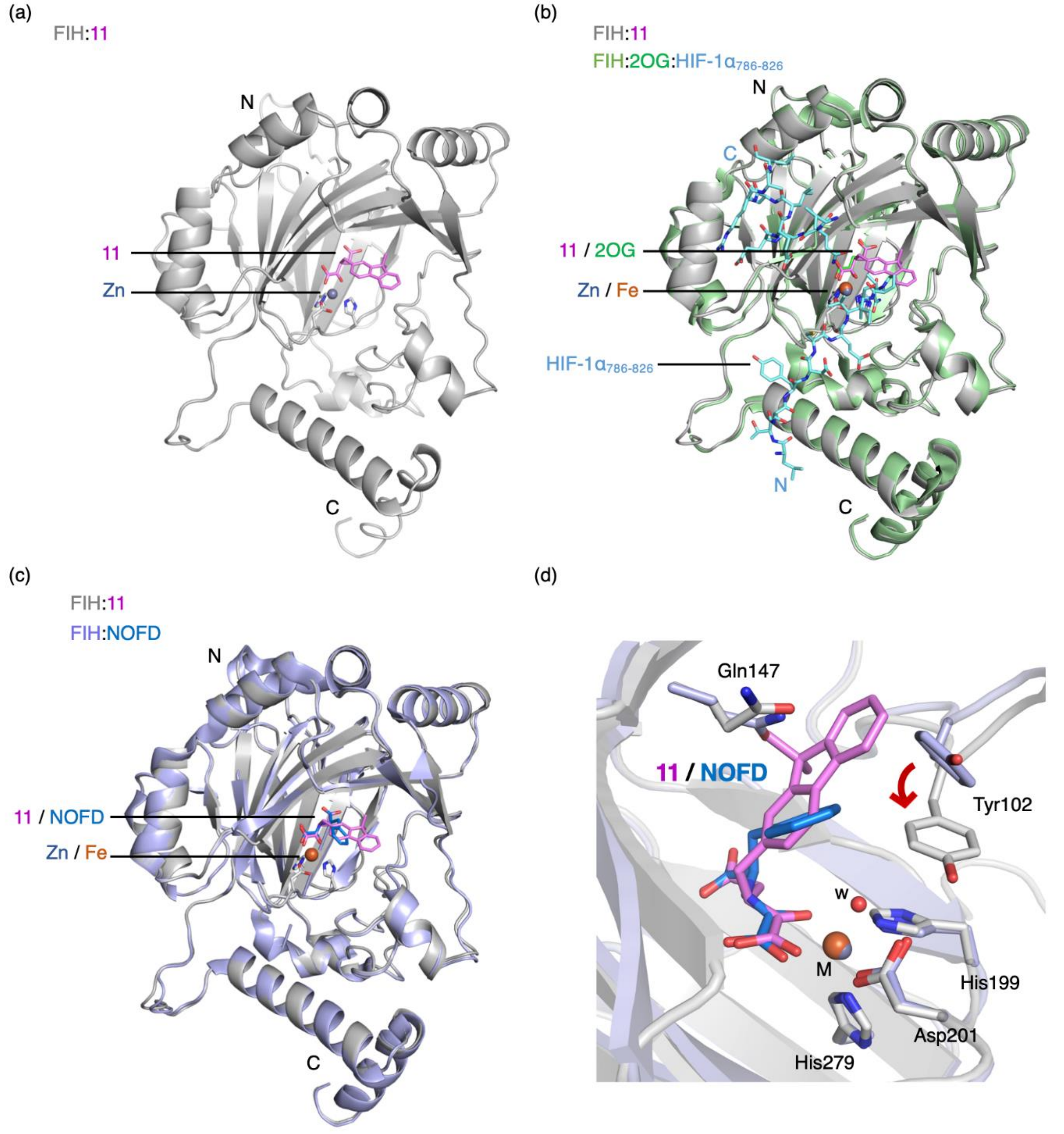

Supplementary Figure 32. Analysis of electron density maps supports the predominant presence of the (*S*)-enantiomer of 3-((9,9-dimethyl-9*H*-fluoren-2-yl)methyl)-2OG (11) in the FIH:11 structure (PDB ID: 7A1K). Color code: FIH: grey; carbon-backbone of (*S*)-3-((9,9-dimethyl-9*H*-fluoren-2-yl)methyl)-2OG ((*S*)-**11**): violet; carbon-backbone of (*R*)-3-((9,9-dimethyl-9*H*-fluoren-2-yl)methyl)-2OG ((*R*)-**11**): deep green; Zn: lavender blue; water: red sphere; oxygen: red; nitrogen: blue.

(**a**) Representative OMIT electron density map (mF_o-DF_c) contoured to 2σ around (*S*)-**11** modelled in the FIH:**11** structure (blue mesh). Electron density maps (F_o-F_c) contoured to 2σ and -2σ around (*S*)-**11** are shown in green and red mesh, respectively. The C3-C6-C7 bond angle of (*S*)-**11** (model value: 118.4°) is similar to the predicted value (112.3°); (**b**) representative OMIT electron density map (mF_o-DF_c) contoured to 2σ around (*R*)-**11** modelled in the FIH:(*R*)-**11** structure (blue mesh). Electron density maps (F_o-F_c) contoured to 2σ and -2σ around (*R*)-**11** are shown in green and red mesh, respectively. The C3-C6-C7 bond angle of (*R*)-**11** derives from the predicted value (112.3°) by 9.4σ (model value: 84.2°), supporting the predominant (at least) presence of (*S*)-**11** of the FIH:**11** structure. This proposal is consistent with the binding mode of *N*-(carboxycarbonyl)-D-phenylalanine (NOFD) to FIH;[5] Note, that the nitrogen atom of NOFD affects the Cahn-Ingold-Prelog priority rules resulting in its formal assignment as (*R*)-enantiomer.

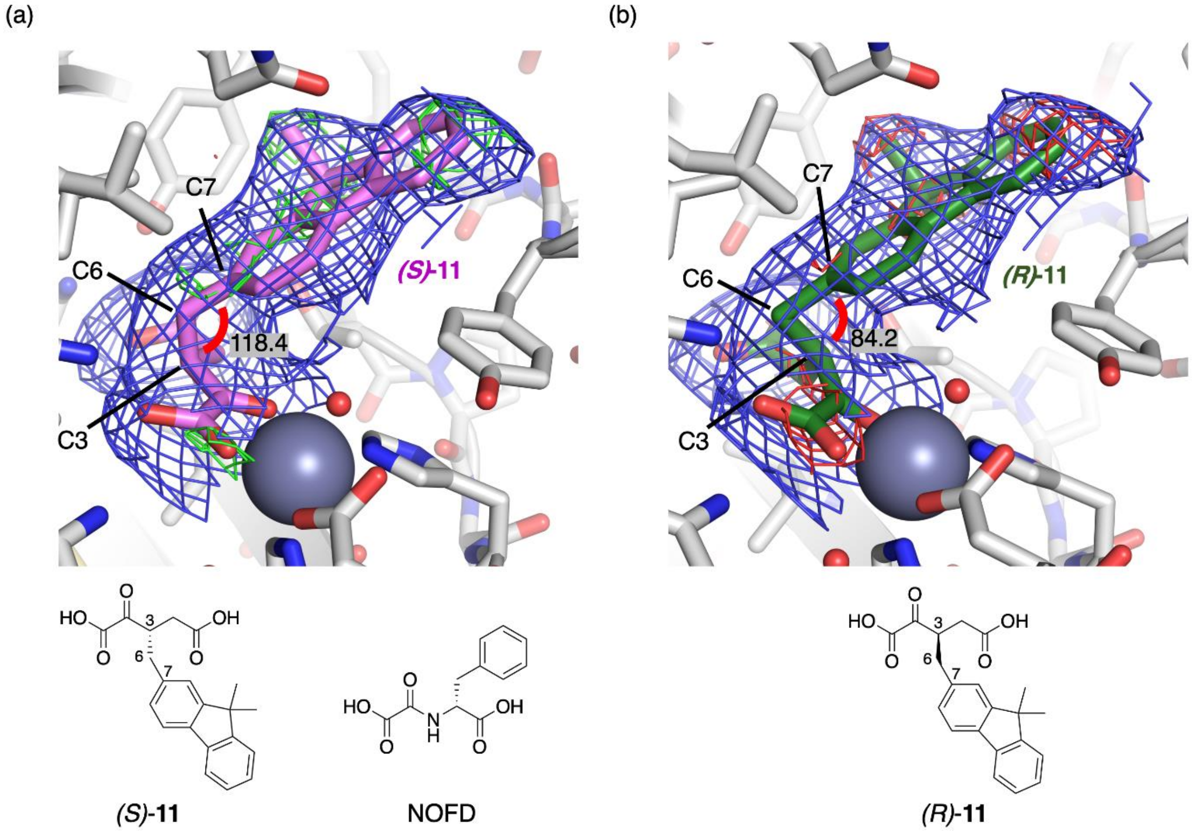

Supplementary Figure 33. Substrate and cosubstrate binding affect the conformation of the sidechain of Tyr102$_{\mathbf{FIH}}$. Color code: FIH: grey; carbon-backbone of 3-((9,9-dimethyl-9*H*-fluoren-2-yl)methyl)-2OG (**11**): violet; Zn: lavender blue; oxygen: red; nitrogen: blue.

(**a**) Superimposition of a view from the FIH:**11** structure (Supplementary Figure 31) with one from the reported FIH:NOG:*D*-Leu-CA$_{1\text{-}20}$ structure (FIH: lavender, carbon-backbone of *N*-oxalylglycine (NOG): marine blue, carbon-backbone of the synthetic ankyrin *D*-Leu-CA$_{1\text{-}20}$ peptide (sequence: HLEVVKLLLEHGADV(*D*-Leu)AQDK): sand; PDB ID: 6RUJ)[12] reveals similar FIH conformations (Cα RMSD = 0.25 Å); (**b**) superimposition of an active site view from the FIH:**11** structure (Supplementary Figure 31) with those from the reported FIH:NOG:*D*-Leu-CA$_{1\text{-}20}$ (PDB ID: 6RUJ)[12] and FIH:2OG:HIF-1α$_{786\text{-}826}$ structures (FIH: pale green, carbon-backbone of 2OG: green, carbon-backbone of the HIF-1α$_{786\text{-}826}$ substrate peptide: cyan, Fe: orange; PDB ID: 1H2L)[9] reveals that the sidechain of Tyr102$_{\text{FIH}}$ occupies different conformations. In both the FIH:**11** and the FIH:NOG:*D*-Leu-CA$_{1\text{-}20}$ structures, the sidechain of Tyr102$_{\text{FIH}}$ is rotated compared to its conformation in the FIH:2OG:HIF-1α$_{786\text{-}826}$ structure, likely to avoid a clash with the C3-substituent of **11** or the *D*-Leu-CA$_{1\text{-}20}$ substrate, respectively.

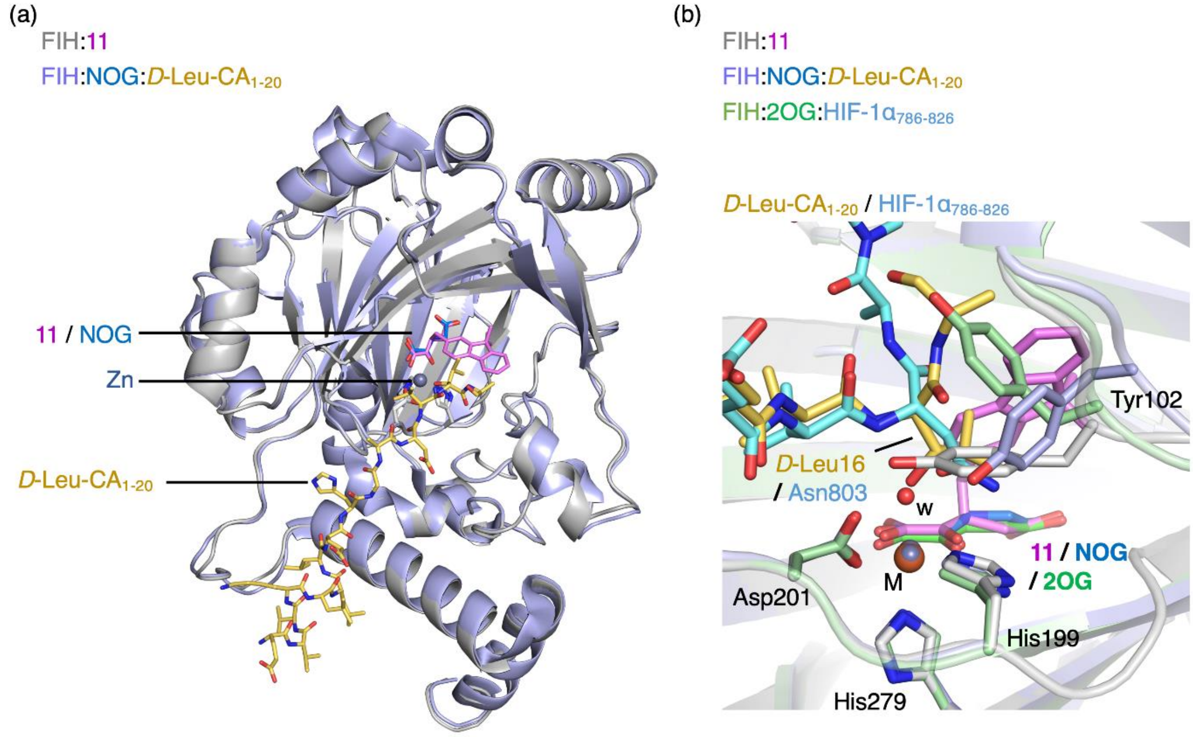

Supplementary Figure 34. ^{1}H and ^{13}C NMR spectra of compound 37. NMR spectra were recorded in $CDCl_3$ using a Bruker AVANCE AVIIIHD 600 machine equipped with a 5 mm BB-F/1H Prodigy N_2 cryoprobe operated using Bruker TopSpin software (version 3.6.1). Spectra were analyzed and processed using Bruker TopSpin 3.6.1.

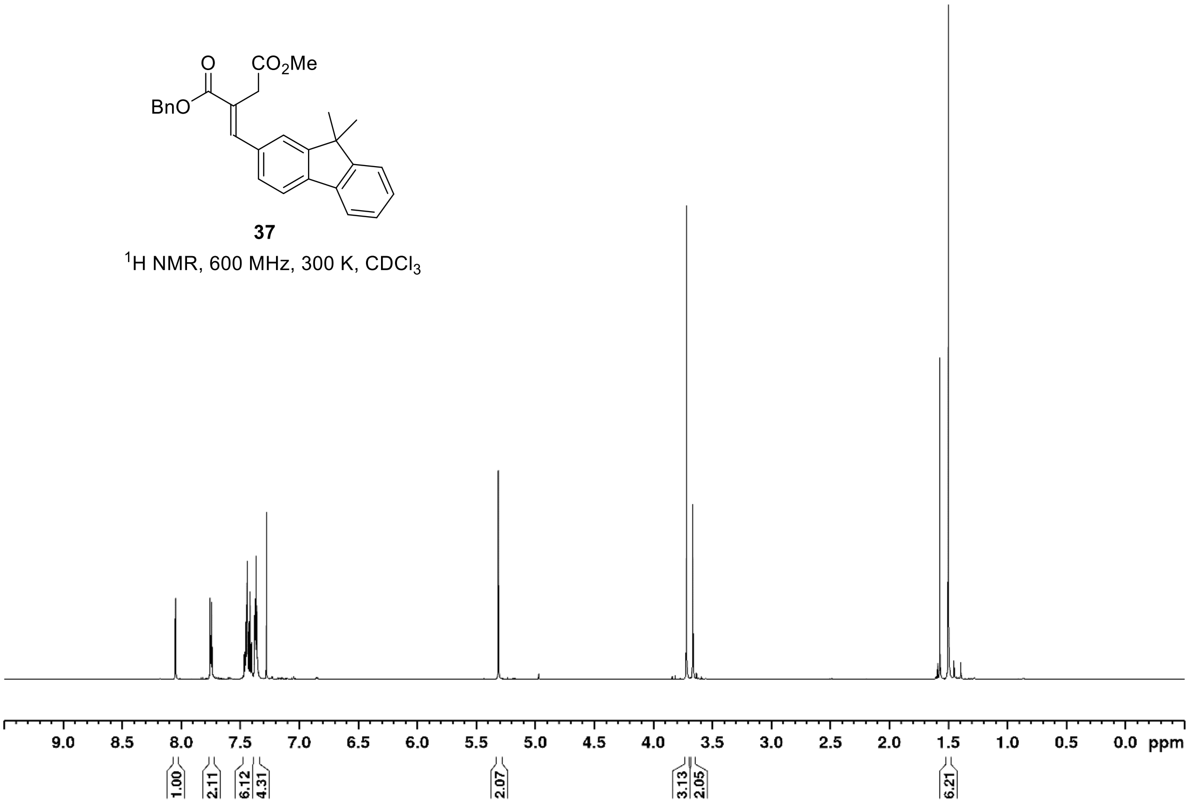

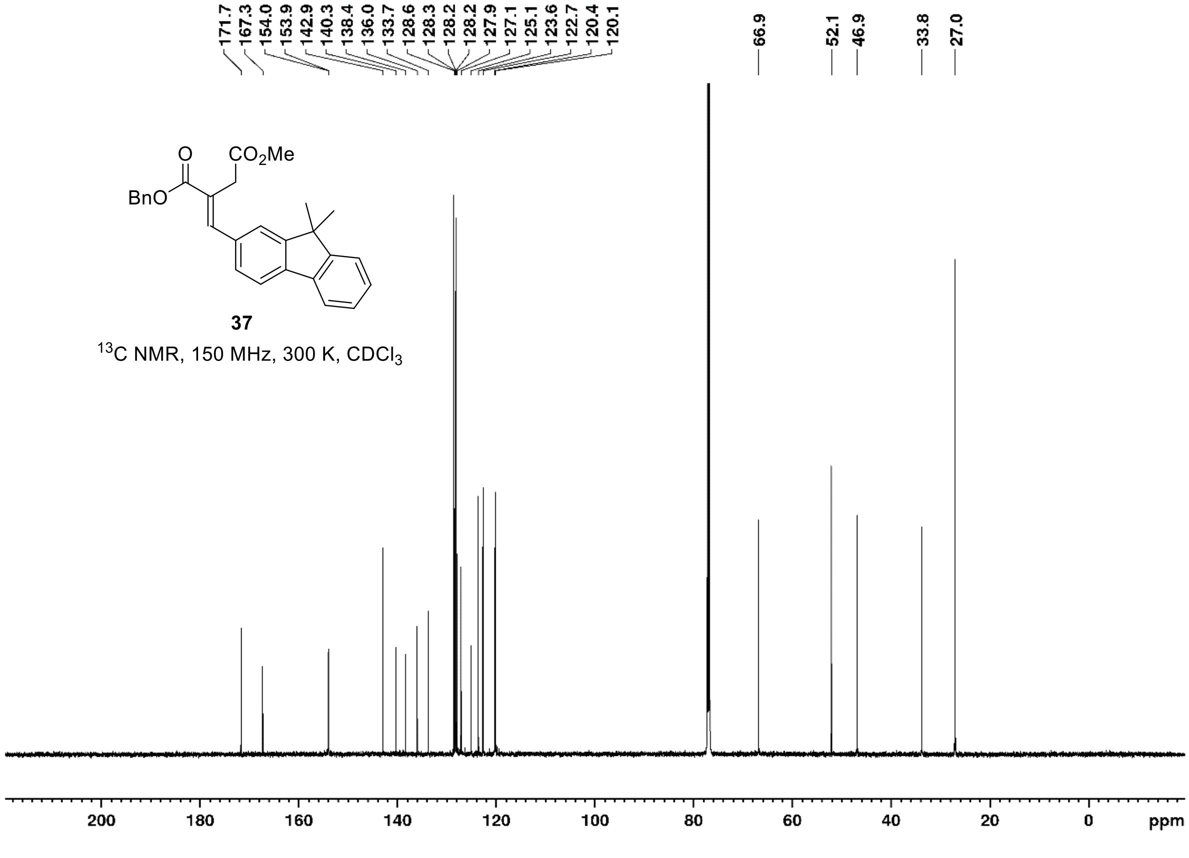

Supplementary Figure 35. ^{1}H and ^{13}C NMR spectra of compound 38. NMR spectra were recorded in $CDCl_3$ using a Bruker AVANCE AVIIIHD 600 machine equipped with a 5 mm BB-F/1H Prodigy N_2 cryoprobe operated using Bruker TopSpin software (version 3.6.1). Spectra were analyzed and processed using Bruker TopSpin 3.6.1.

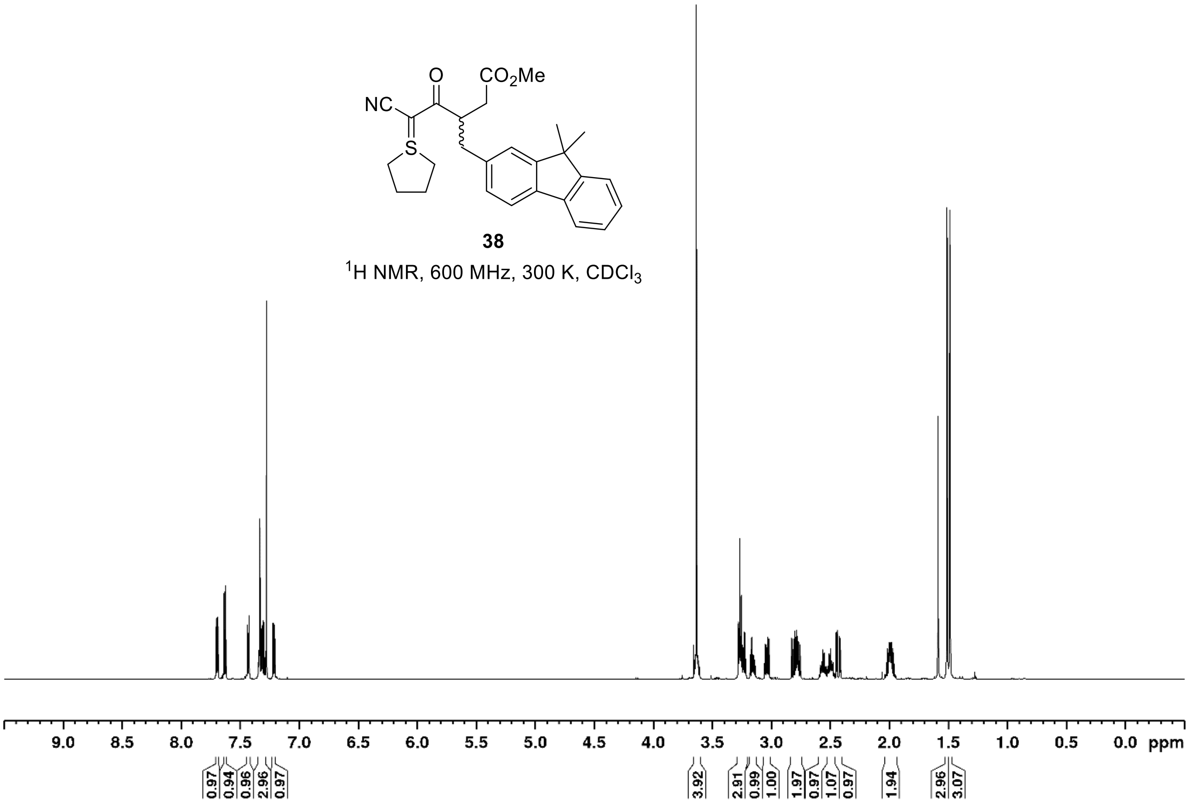

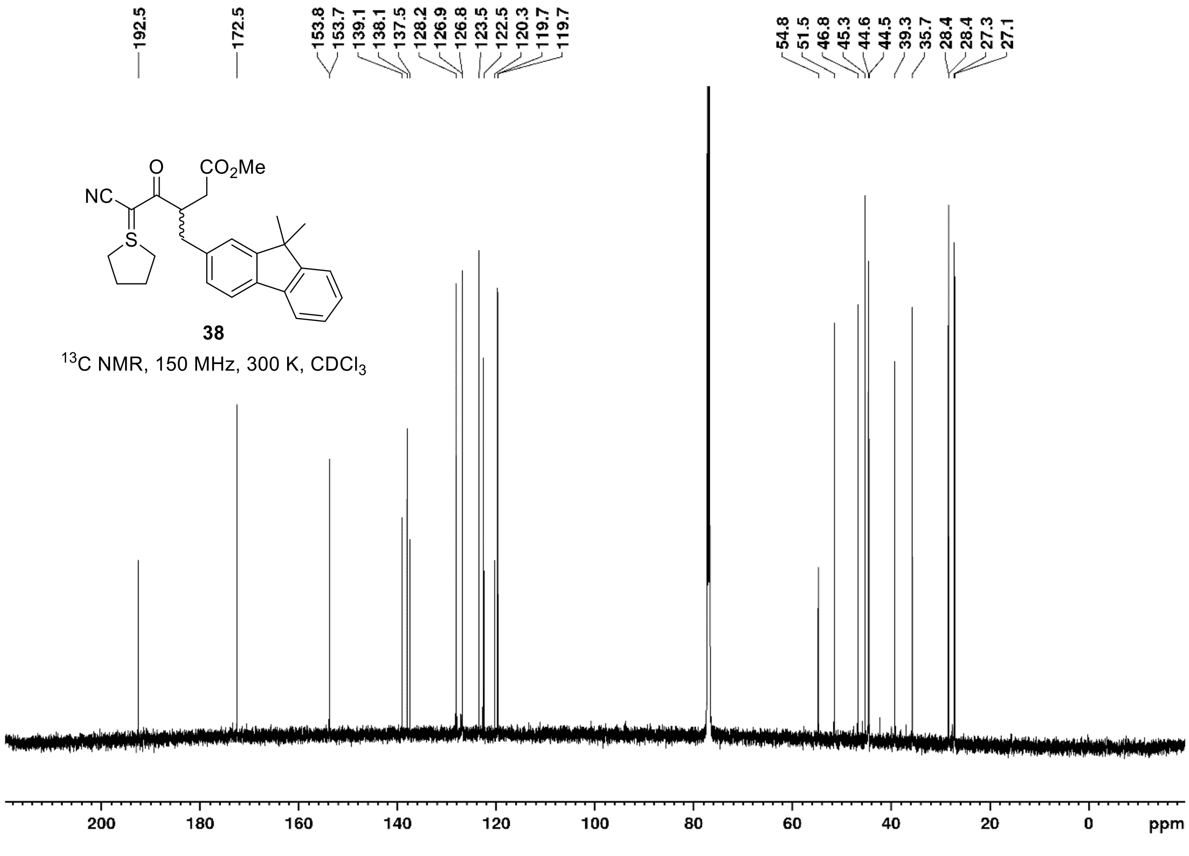

Supplementary Figure 36. ^{1}H and ^{13}C NMR spectra of compound 39. NMR spectra were recorded in $CDCl_3$ using a Bruker AVANCE AVIIIHD 600 machine equipped with a 5 mm BB-F/1H Prodigy N_2 cryoprobe operated using Bruker TopSpin software (version 3.6.1). Spectra were analyzed and processed using Bruker TopSpin 3.6.1.

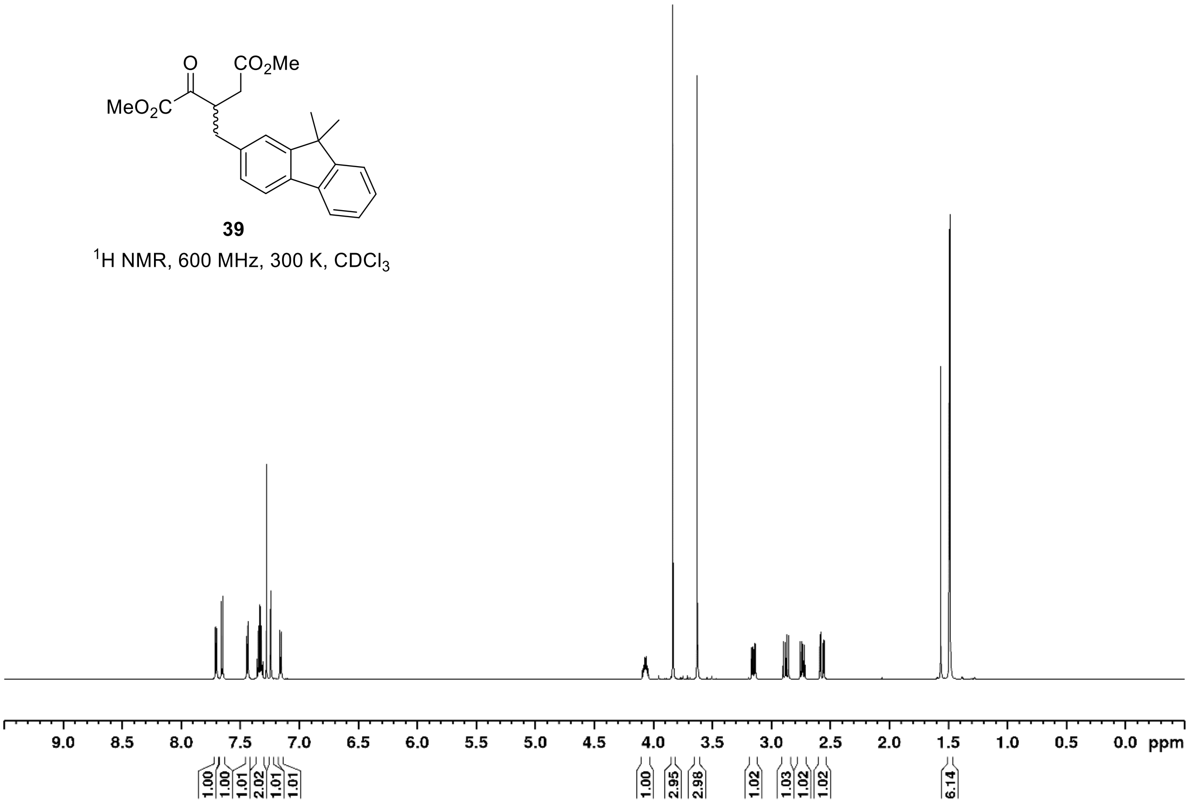

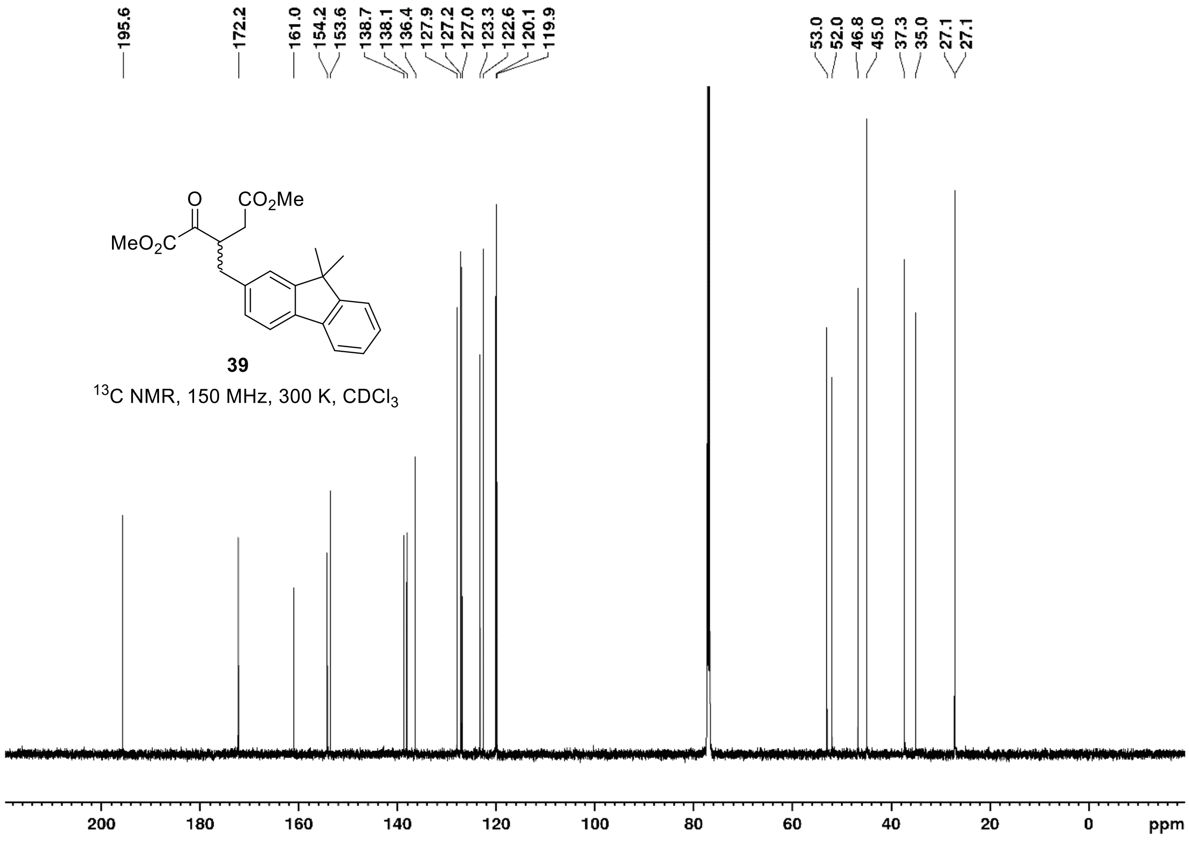

Supplementary Figure 37. ^{1}H and ^{13}C NMR spectra of compound 11. NMR spectra were recorded in $CDCl_3$ using a Bruker AVANCE AVIIIHD 600 machine equipped with a 5 mm BB-F/1H Prodigy N_2 cryoprobe operated using Bruker TopSpin software (version 3.6.1). Spectra were analyzed and processed using Bruker TopSpin 3.6.1.

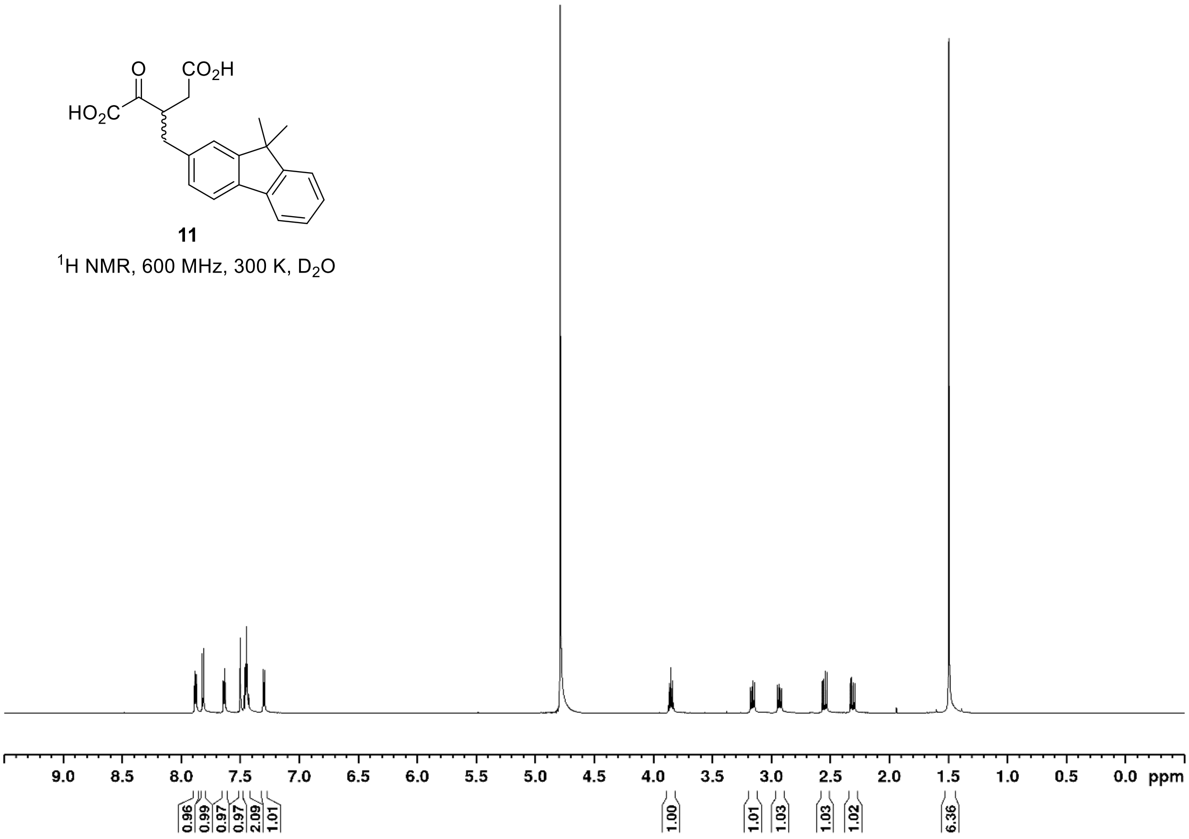

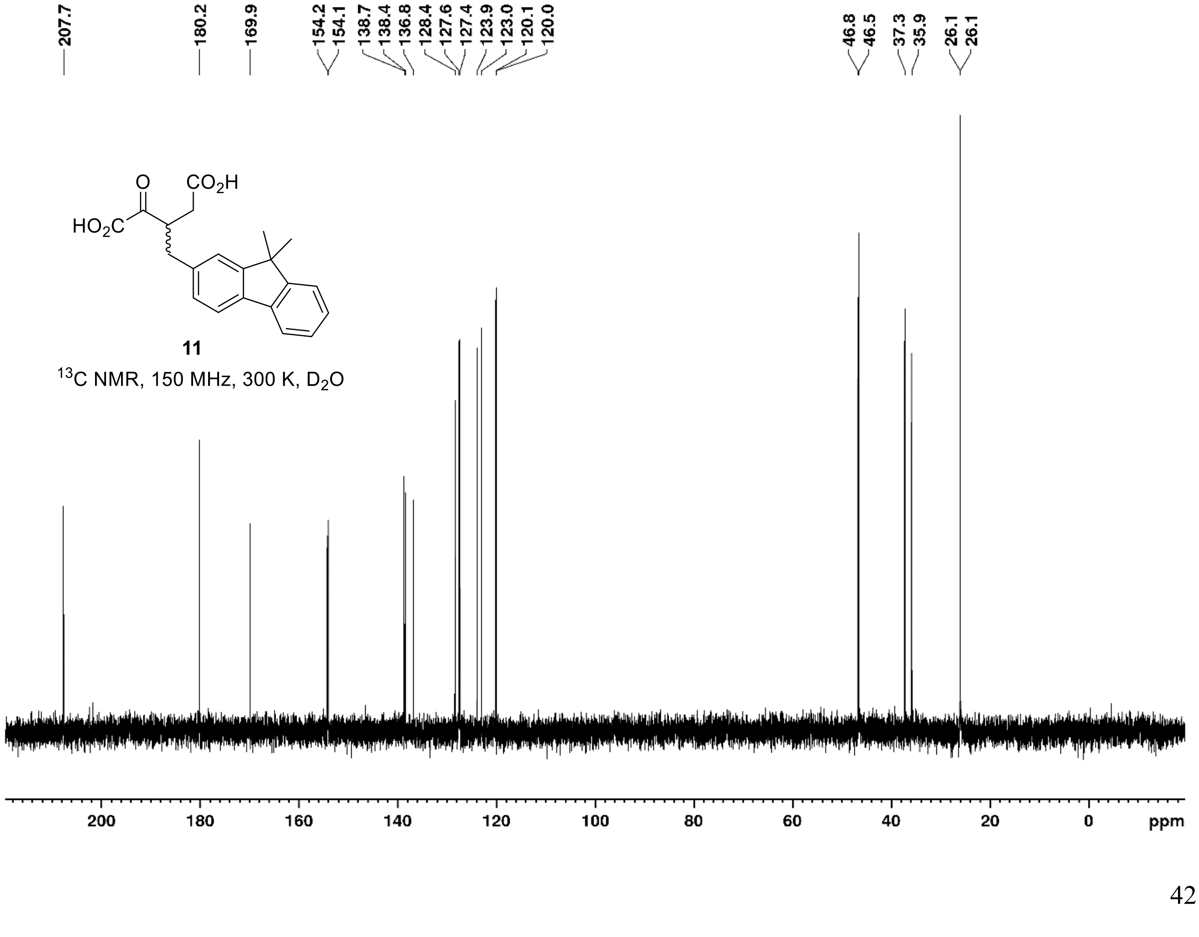

Supplementary Figure 38. ^{1}H and ^{13}C NMR spectra of compound 40. NMR spectra were recorded in $CDCl_3$ using a Bruker AVANCE AVIIIHD 600 machine equipped with a 5 mm BB-F/1H Prodigy N_2 cryoprobe operated using Bruker TopSpin software (version 3.6.1). Spectra were analyzed and processed using Bruker TopSpin 3.6.1.

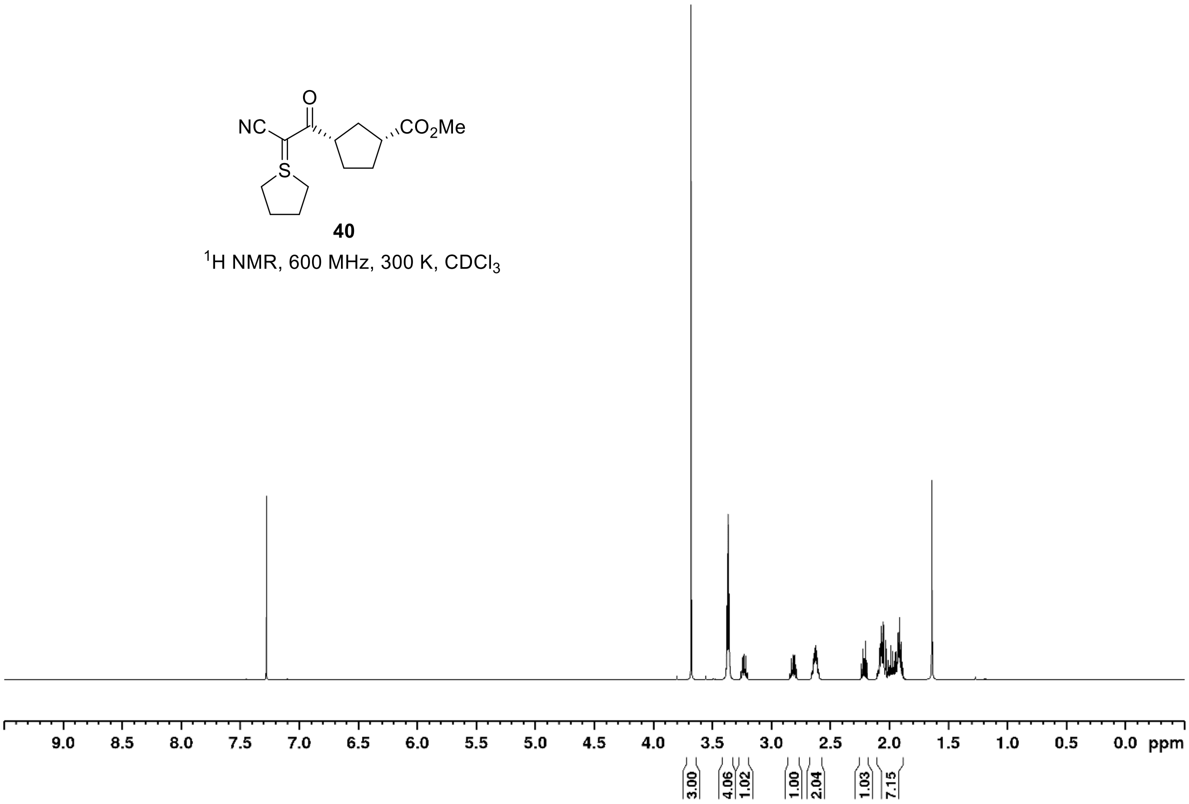

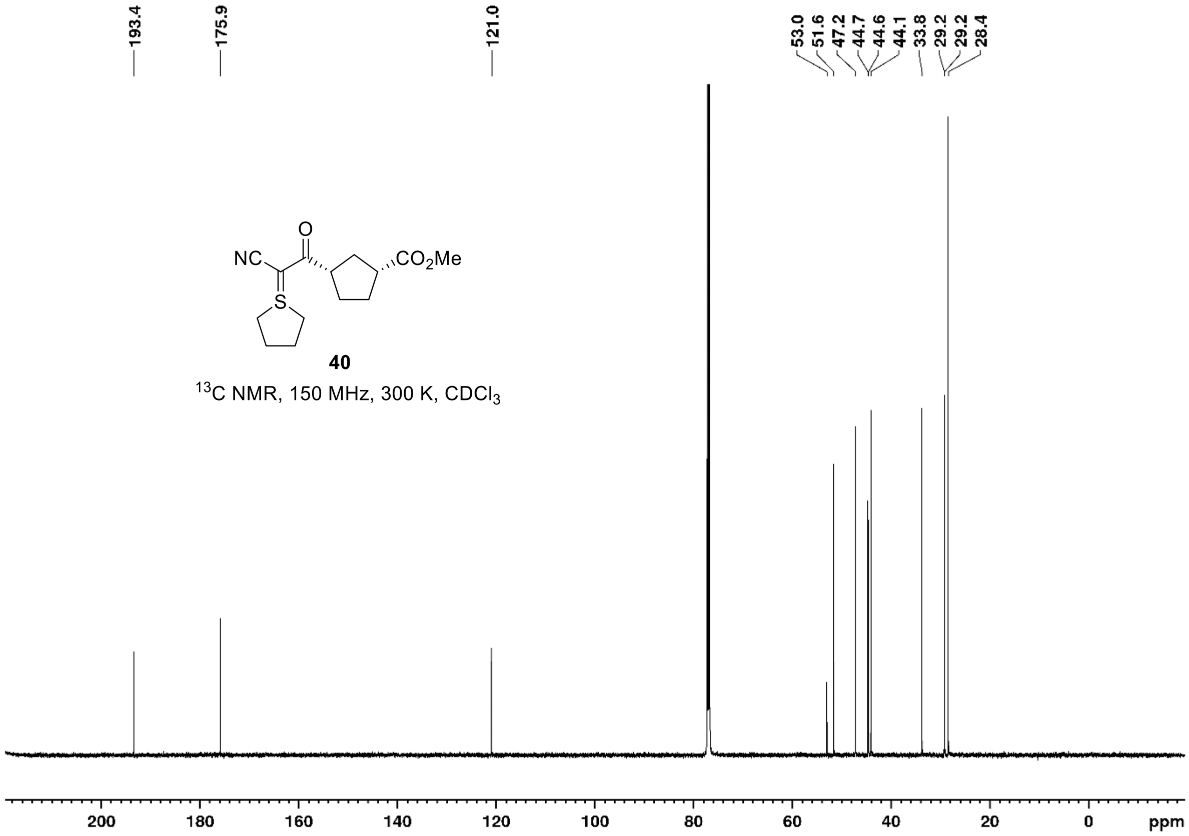

Supplementary Figure 39. ^{1}H and ^{13}C NMR spectra of compound 41. NMR spectra were recorded in $CDCl_3$ using a Bruker AVANCE AVIIIHD 600 machine equipped with a 5 mm BB-F/1H Prodigy N_2 cryoprobe operated using Bruker TopSpin software (version 3.6.1). Spectra were analyzed and processed using Bruker TopSpin 3.6.1.

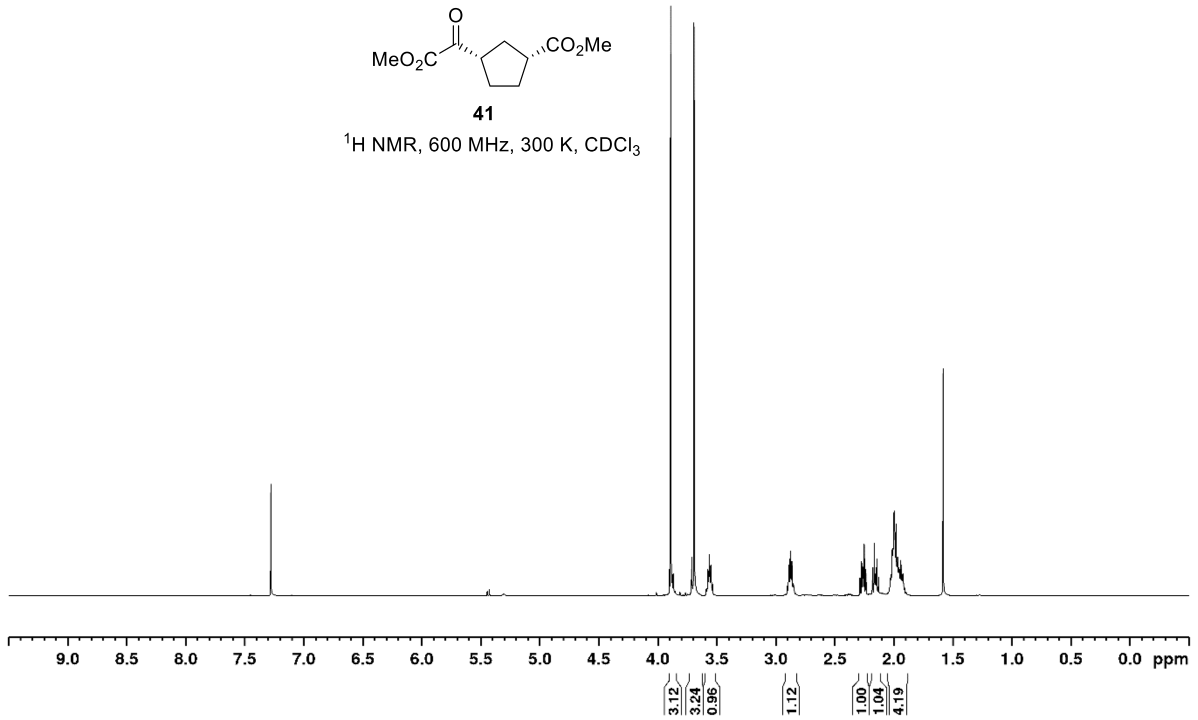

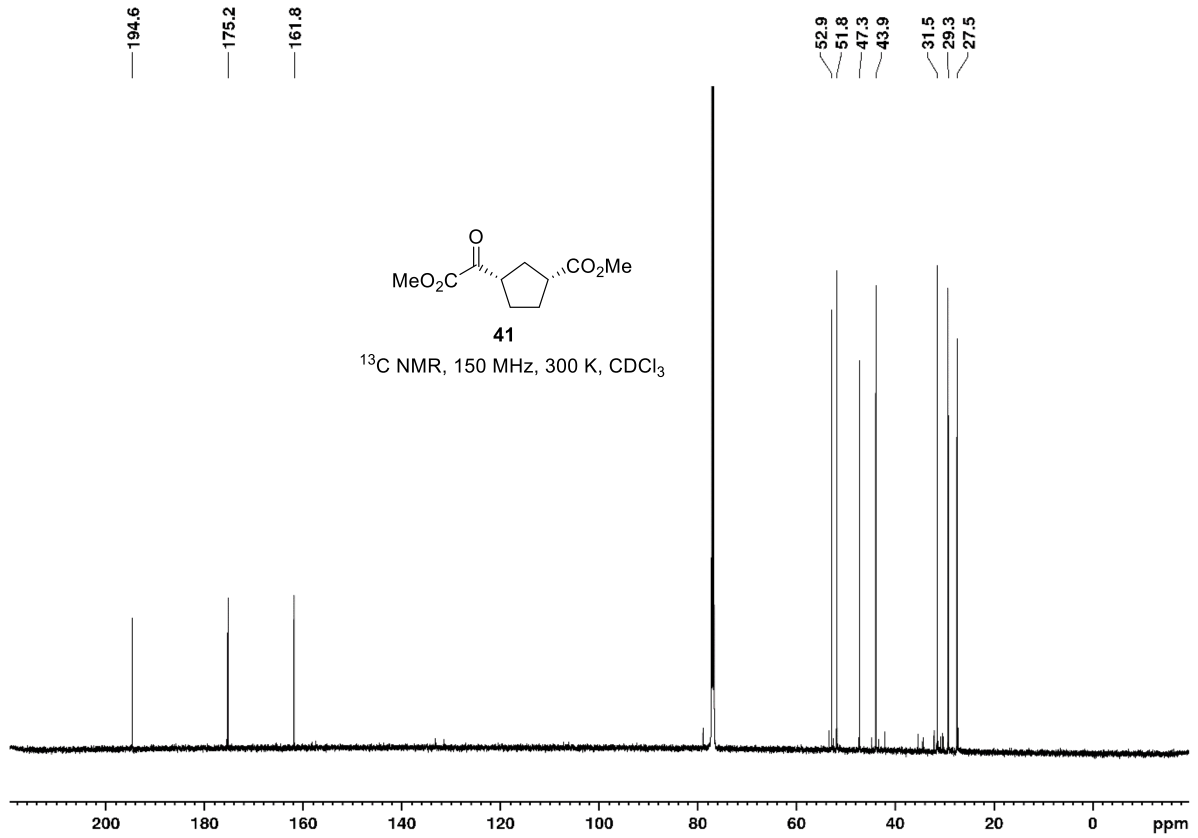

Supplementary Figure 40. ^{1}H and ^{13}C NMR spectra of compound 22. NMR spectra were recorded in $CDCl_3$ using a Bruker AVANCE AVIIIHD 600 machine equipped with a 5 mm BB-F/1H Prodigy N_2 cryoprobe operated using Bruker TopSpin software (version 3.6.1). Spectra were analyzed and processed using Bruker TopSpin 3.6.1.

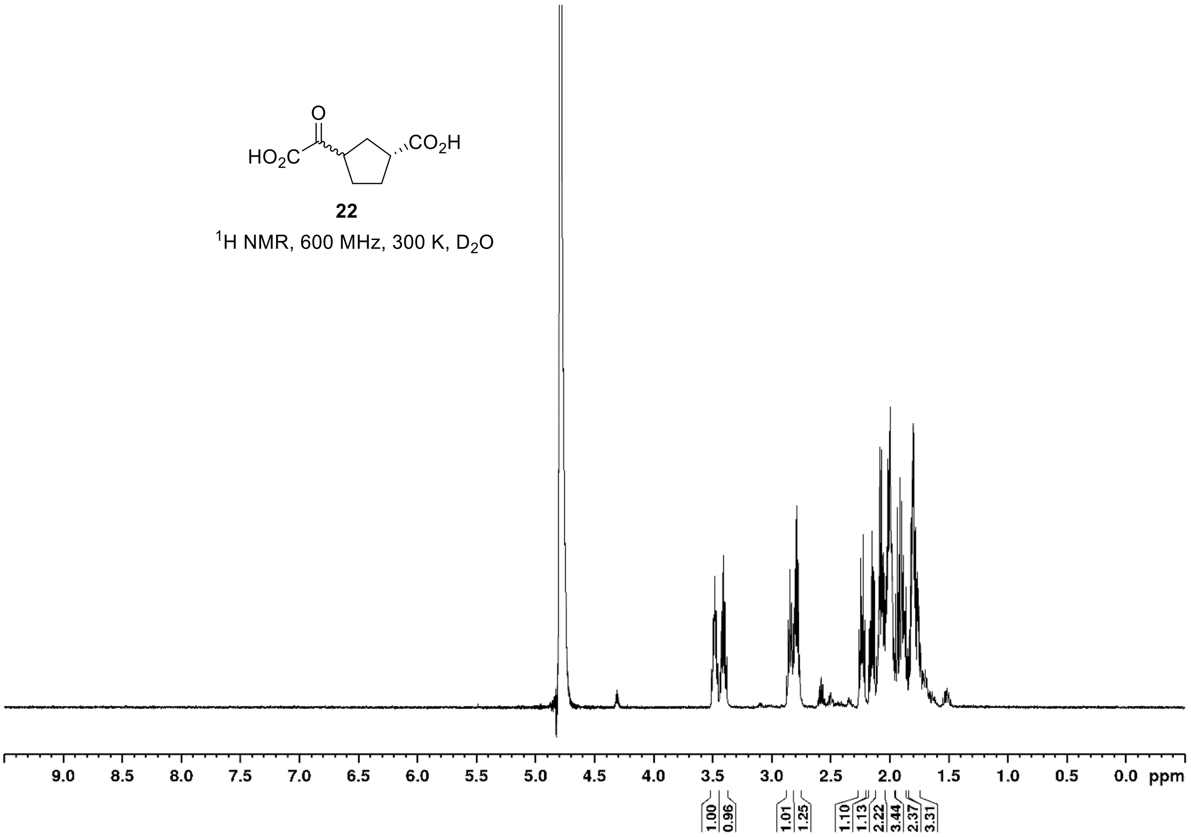

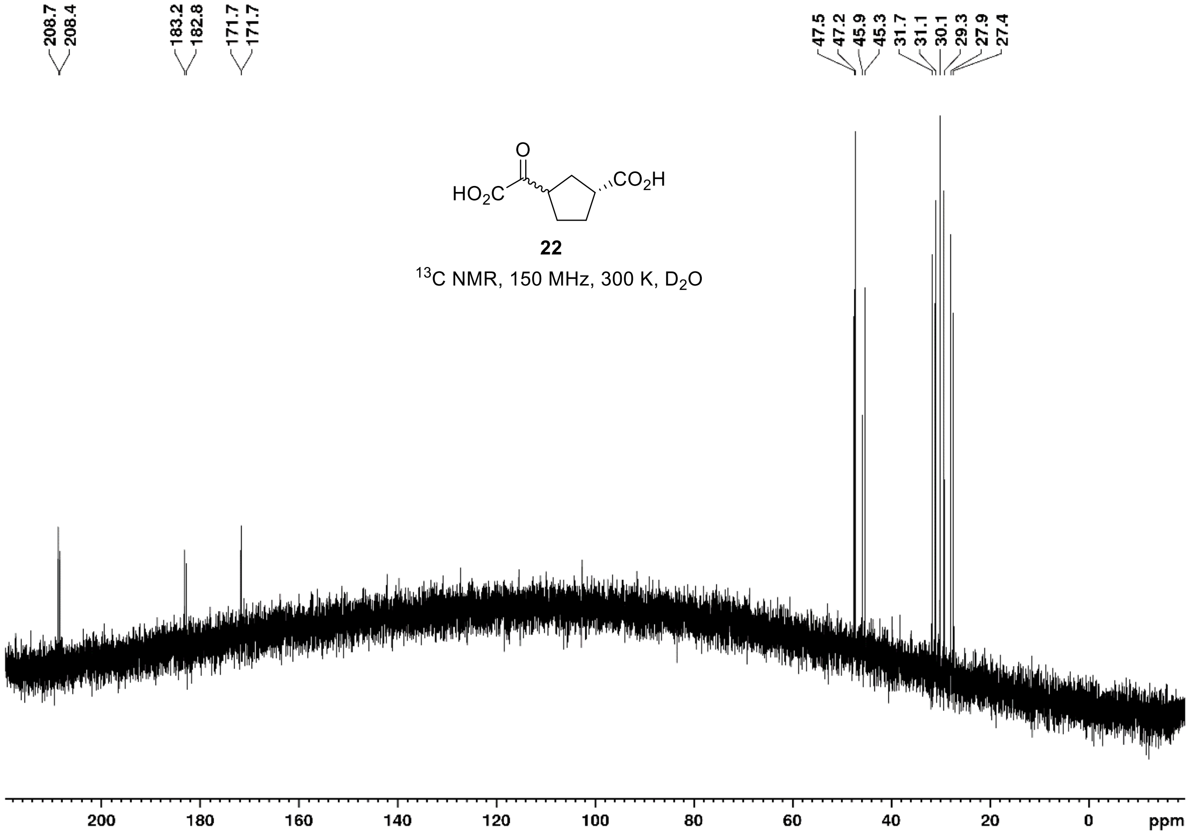

2. Supplementary Tables

Supplementary Table 1. 2OG derivatives have the potential to selectively inhibit 2OG oxygenases (continues on the following page). SPE-MS FIH inhibition assays were performed as described in the Methods section. Conditions: 0.15 µM FIH, 5.0 µM HIF-1$\alpha_{788-822}$[4], 100 µM LAA, 10 µM FAS, and 10 µM 2OG in buffer (50 mM Tris, 50 mM NaCl, pH 7.5, 20º C). Reported SPE-MS AspH inhibition assay conditions[1]: 0.05 µM AspH, 1.0 µM substrate peptide (hFX-$CP_{101-119}$), 100 µM LAA, 2.0 µM FAS, and 3.0 µM 2OG in buffer (50 mM HEPES, pH 7.5, 20º C).

The reported partially selective FIH inhibitor *N*-oxalyl-D-phenylalanine (NOFD)[5] was used as a positive inhibition control (IC_{50} ~ 0.3 µM, Entry 1). Of the 35 2OG derivatives investigated for FIH inhibition, 17 inhibit FIH activity, while 23 have been reported to inhibit AspH[1]. Of the 17 identified FIH inhibitors, 4-(3-phenylpropyl)-2OG **18** (IC_{50} ~ 0.9 µM, Entry 19) and 3-((9,9-dimethyl-9*H*-fluoren-2-yl)methyl)-2OG **11** (IC_{50} ~ 1.9 µM, Entry 12) displayed highest potency. All of the 17 identified FIH inhibitors have been reported to also inhibit AspH activity. The comparison with the reported inhibition data for AspH[1] reveals that, in general, the synthetic 2OG derivatives inhibit AspH more efficiently than FIH. For example, 4-isobutyl-2OG (**16**) inhibits AspH ~70 fold more selective than FIH as judged by IC_{50}-values (IC_{50} ~ 0.5 µM for AspH and IC_{50} ~ 35.3 µM for FIH; Entry 17). Note, however, that the SPE-MS AspH inhibition assays were performed at different enzyme, substrate, 2OG, and Fe(II) concentrations (0.05 µM, 1.0 µM, 3.0 µM, respectively 2.0 µM for AspH and 0.15 µM, 5.0 µM, 10.0 µM, respectively 10.0 µM for FIH) and in a different reaction buffer (50 mM HEPES, pH 7.5 for AspH and 50 mM Tris, 50 mM NaCl, pH 7.5 for FIH), which might, at least in part, reflect the observed differences of the synthetic 2OG derivatives to inhibit the two 2OG oxygenases. Low levels of selective FIH inhibition was observed for 3-propyl-2OG (**3**), which appears to inhibit AspH with twofold reduced potency than FIH (Entry 4). The results indicated the potential of 3-((9,9-dimethyl-9*H*-fluoren-2-yl)methyl)-2OG **11** for selective FIH inhibition (Entry 12), however, the experimental error reported for AspH inhibition prohibits an exact comparison of the IC_{50}-values.

While the identified FIH inhibitors did not display substantial selectivity for FIH inhibition, perfectly selective AspH over FIH inhibition was observed for 2OG derivatives **10**, **13**, **22-24**, and **35**. Of these selective AspH inhibitors, 4,4-dimethyl-2OG (**13**) is the most efficient AspH inhibitor (IC_{50} ~ 0.3 µM, Entry 14), and thus bears the potential for use in functional assignment and cellular AspH inhibition studies.

	[a]2OG derivative	IC_{50} FIH [μM]	IC_{50} AspH[1] [μM]		[a]2OG derivative	IC_{50} FIH [μM]	IC_{50} AspH[1] [μM]		[a]2OG derivative	IC_{50} FIH [μM]	IC_{50} AspH[1] [μM]
1	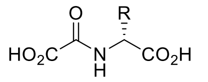 **NOFD**: R = CH_2Ph	0.3 ± 0.1	15.5 ± 3.1[13]	13	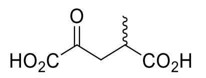 **12**	inactive	inactive	[h]25	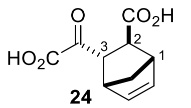	inactive	19.3 ± 1.6
2	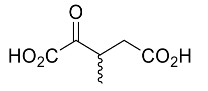 **1**	inactive	inactive	14	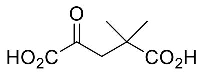 **13**	inactive	0.31 ± 0.10	26	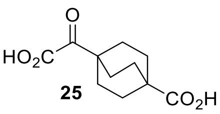	inactive	inactive
3	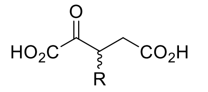 **2**: R = CH_2CH_3	4.3 ± 0.1	1.2 ± 0.5	15	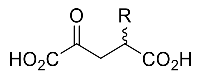 **14**: R = CH_2CH_3	19.3 ± 7.7	0.61 ± 0.09	27	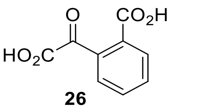 **26**	inactive	inactive
4	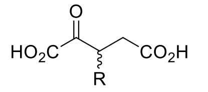 **3**: R = $CH_2CH_2CH_3$	2.7 ± 0.1	5.7 ± 1.1	16	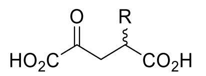 **15**: R = $CH_2CH_2CH_3$	14.8 ± 0.7	0.47 ± 0.08	28	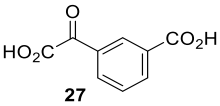 **27**	inactive	inactive
5	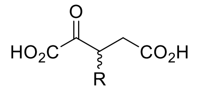 **4**: R = $CH_2CH_2C(CH_3)_3$	31.0 ± 0.1	48.2 ± 13.1	17	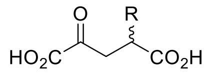 **16**: R = $CH_2CH(CH_3)_2$	35.3 ± 2.0	0.51 ± 0.12	29	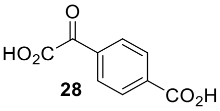	inactive	inactive
6	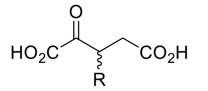 **5**: R = $CH_2CH_2CH_2Ph$	5.7 ± 0.6	6.8 ± 0.9	18	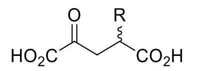 **17**: R = $CH_2CH_2C(CH_3)_3$	38.9 ± 2.1	0.70 ± 0.11	30	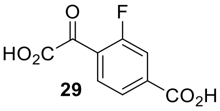	inactive	inactive
7	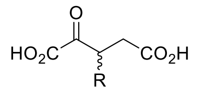 **6**: R = CH_2Ph	6.4 ± 0.9	1.6 ± 0.3	19	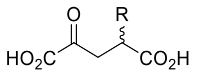 **18**: R = $CH_2CH_2CH_2Ph$	0.9 ± 0.1	0.25 ± 0.05	31	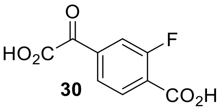	inactive	inactive
8	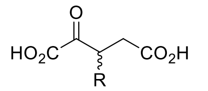 **7**: R = $CH_2(4\text{-}FC_6H_4)$	7.5 ± 0.7	2.6 ± 0.8	20	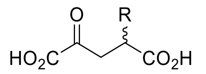 **19**: R = CH_2Ph	3.6 ± 0.2	0.43 ± 0.05	32	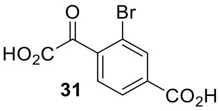	inactive	inactive
9	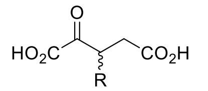 **8**: R = $CH_2(4\text{-}F_3COC_6H_4)$	12.7 ± 1.3	6.3 ± 2.6	21	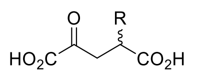 **20**: R = CH_2(2-naphthyl)	2.5 ± 0.1	0.17 ± 0.03	33	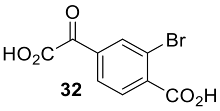	inactive	inactive
10	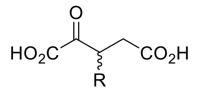 **9**: R = $CH_2(4\text{-}MeOC_6H_4)$	3.6 ± 0.3	3.6 ± 1.4	[f]22	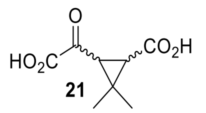	19.4 ± 0.6	5.2 ± 1.7	34	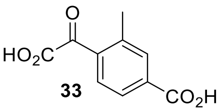	inactive	inactive
11	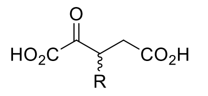 **10**: R = $CH_2(3,5\text{-}Me_2C_6H_4)$	inactive	4.7 ± 0.1	[g]23	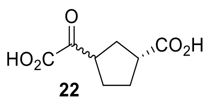	inactive	5.0 ± 1.2	35	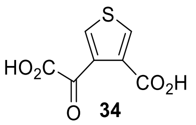	inactive	inactive
12	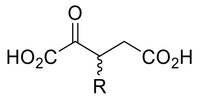 **11**: R = CH_2(9,9-dimethyl-9*H*-fluoren-2-yl)	1.9 ± 0.1	4.3 ± 3.8	24	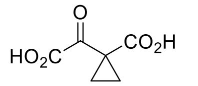 **23**	inactive	3.3 ± 1.0	36	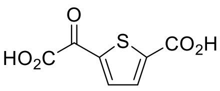 **35**	inactive	12.9 ± 1.3

a) All chiral 2OG derivatives were prepared as racemic mixtures; b) mixture of racemic diastereomers, dr (*cis*:*trans*) = 2.5:1; c) mixture of diastereomers, dr (*cis*:*trans*) = 1:1; d) (±)-(*2-exo,3-endo*)-diastereomer.

Supplementary Table 2. Crystallization conditions, data collection, and refinement statistics for the FIH:2OG derivative complexes without substrate bound.[a]

	FIH·Zn^{II}·**1** (FIH:**1**)	FIH·Zn^{II}·**3** (FIH:**3**)	FIH·Zn^{II}·**11** (FIH:**11**)	FIH·Zn^{II}·**18** (FIH:**18**)
**PDB ID**	7A1L	7A1M	7A1K	7A1J
**Data collection**				
Space group	$P4_12_12$	$P4_12_12$	$P4_12_12$	$P4_12_12$
Cell dimensions:				
a, *b*, *c* (Å)	86.55, 86.55, 148.09	85.95, 85.95, 148.96	86.19, 86.19, 148.74	86.18, 86.18, 148.90
α, β, γ (°)	90.00, 90.00, 90.00	90.00, 90.00, 90.00	90.00, 90.00, 90.00	90.00, 90.00, 90.00
X-Ray source[b]	Synchrotron (DLS I03)	Synchrotron (DLS I04)	Synchrotron (DLS I03)	Synchrotron (DLS I03)
Resolution (Å)[c]	74.05-2.29 (2.33-2.29)	74.45-2.18 (2.22-2.18)	54.58-1.90 (1.93-1.90)	74.59-1.99 (2.02-1.99)
R_{merge}	0.106 (3.519)	0.156 (6.807)	0.056 (5.871)	0.076 (5.665)
$I / \sigma I$	18.3 (1.2)	17.1 (0.5)	25.5 (0.7)	21.5 (0.6)
CC (1/2)	1.000 (0.533)	1.000 (0.414)	0.999 (0.308)	0.999 (0.380)
Total number of reflections	681827 (32251)	724756 (35982)	1169753 (61900)	1027267 (53366)
Total number unique reflections	26157 (1252)	28085 (1487)	44899 (2209)	39248 (1927)
Completeness (%)	99.9 (98.8)	93.7 (100.0)	100.0 (100.0)	100.0 (100.0)
Multiplicity	26.1 (25.8)	25.8 (24.2)	26.1 (28.0)	26.2 (27.7)
**Refinement**				
R_{work} / R_{free}	0.208 / 0.222	0.220 / 0.245	0.174 / 0.209	0.207 / 0.227
No. atoms:	5453	5463	5594	5474
B-factors:	90.0	84.0	80.0	94.0
R.m.s. deviations:				
Bond lengths (Å)	0.002	0.003	0.020	0.009
Bond angles (°)	0.481	0.585	1.317	0.673

[a)]Experimental details are described in the Methods section; [b)]DLS: Diamond Light Source; [c)]Values in parentheses are for highest-resolution shell.

Supplementary Table 3. Crystallization conditions, data collection, and refinement statistics for the FIH:2OG derivative complexes with substrate bound.[a]

	FIH·Zn^{II}·**1**·synthetic consensus ankyrin repeat (FIH:**1**:CA_{1-20})	FIH·Zn^{II}·**14**·synthetic consensus ankyrin repeat (FIH:**14**:CA_{1-20})	FIH·Zn^{II}·**15**·synthetic consensus ankyrin repeat (FIH:**15**:CA_{1-20})	FIH·Zn^{II}·**22**·synthetic consensus ankyrin repeat (FIH:**22**:CA_{1-20})	FIH·Zn^{II}·**1**·tankyrase-2 (FIH:**1**:$TNKS2_{691-710}$)
**PDB ID**	7A1N	7A1O	7A1P	7A1Q	7A1S
**Data collection**					
Space group	$P4_12_12$	$P4_12_12$	$P4_12_12$	$P4_12_12$	$P4_12_12$
Cell dimensions:					
a, b, c (Å)	86.05, 86.05, 147.83	86.29, 86.29, 146.93	85.95, 85.95, 147.63	85.97, 85.97, 148.18	86.09, 86.09, 148.42
α, β, γ (°)	90.00, 90.00, 90.00	90.00, 90.00, 90.00	90.00, 90.00, 90.00	90.00, 90.00, 90.00	90.00, 90.00, 90.00
X-Ray source[b]	Synchrotron (DLS I04)	Synchrotron (DLS I04)	Synchrotron (DLS I04)	Synchrotron (DLS I04)	Synchrotron (DLS I03)
Resolution (Å)[c]	74.37-2.01 (2.04-2.01)	74.41-2.21 (2.25-2.21)	74.28-1.76 (1.79-1.76)	56.12-1.75 (1.78-1.75)	74.47-2.01 (2.04-2.01)
R_{merge}	0.112 (6.269)	0.155 (6.556)	0.076 (6.504)	0.059 (8.138)	0.219 (12.392)
I / σI	19.8 (0.6)	15.8 (0.5)	22.5 (0.6)	26.6 (0.4)	13.5 (0.5)
CC (1/2)	1.000 (0.373)	0.999 (0.168)	1.000 (0.365)	1.000 (0.437)	0.999 (0.351)
Total number of reflections	993295 (50076)	674360 (13207)	1481946 (76943)	1522127 (78100)	983869 (51652)
Total number unique reflections	37759 (1845)	25789 (788)	55608 (2745)	56748 (2782)	38135 (1861)
Completeness (%)	100.0 (100.0)	90.5 (56.8)[d]	100.0 (100.0)	100.0 (100.0)	100.0 (100.0)
Multiplicity	26.3 (27.1)	26.1 (16.8)	26.6 (28.0)	26.8 (28.1)	25.8 (27.8)
**Refinement**					
R_{work} / R_{free}	0.213 / 0.234	0.210 / 0.243	0.161 / 0.191	0.177 / 0.200	0.180 / 0.209
No. atoms:	5769	5854	5960	5864	5971
B-factors:	77.0	83.0	56.0	61.0	64.0
R.m.s. deviations:					
Bond lengths (Å)	0.002	0.002	0.010	0.005	0.002
Bond angles (°)	0.541	0.518	1.017	0.844	0.513

[a]Experimental details are described in the Methods section; [b]DLS: Diamond Light Source; [c]Values in parentheses are for highest-resolution shell; [d]Ellipsoidal completeness, as defined by autoPROC/STARANISO (Methods section).

3. Supplementary Methods

3.1 General information

Unless otherwise stated, all reagents were from commercial sources (Sigma-Aldrich, Inc.; Fluorochem Ltd; Alfa Aesar). Oxone, obtained from Alfa Aesar, was used to oxidize cyanosulfur ylids according to a literature protocol[2]. With the exception of 2OG derivatives **11** and **22**, all 2OG derivatives used in this study were synthesized according to a reported procedure[1]. All chiral compounds were prepared as racemates, except for the reported FIH inhibitor *N*-oxalyl-D-phenylalanine[5] (NOFD), which was prepared as a single enantiomer[14]. Anhydrous solvents were from Sigma-Aldrich, Inc. and kept under an atmosphere of nitrogen. Solvents, liquids, and solutions were transferred using nitrogen-flushed stainless steel needles and syringes. All reactions were carried out under an atmosphere of nitrogen unless stated otherwise. Milli-Q® Ultrapure (MQ-grade) water was used for buffers; LCMS grade solvents (Merck) were used for solid phase extraction coupled to mass spectrometry (SPE-MS).
Purifications were performed using an automated Biotage Isolera One purification machine (wavelengths monitored: 254 and 280 nm) equipped with pre-packed Biotage® SNAP KP-Sil or Biotage® SNAP Ultra flash chromatography cartridges. The cartridge size and solvent gradients (in column volumes, CV) used are specified in the experimental procedures. HPLC grade solvents (acetone, cyclohexane, dichloromethane, ethyl acetate, methanol; Sigma-Aldrich, Inc.) were used for purifications, reaction work-ups, and extractions.
Thin layer chromatography (TLC) employed Merck silica gel 60 F_{254} TLC plates and visualized under UV light and by using appropriate staining solutions (e.g. Hanessian's stain). Melting points (m.p.) were determined using a Stuart SMP-40 automated melting point apparatus. Infrared (IR) spectroscopy was performed using a Bruker Tensor-27 Fourier transform infrared (FT-IR) spectrometer. High-resolution mass spectrometry (HRMS) was performed using electro-spray ionization (ESI) mass spectrometry (MS) in the positive or negative ionization modes employing a Thermo Scientific Exactive mass spectrometer (ThermoFisher Scientific); data are presented as a mass-to-charge ratio (*m/z*).
Nuclear magnetic resonance (NMR) spectroscopy was performed using a Bruker AVANCE AVIIIHD 600 machine equipped with a 5 mm BB-F/1H Prodigy N_2 cryoprobe operated using Bruker TopSpin software (version 3.6.1). Spectra were analyzed and processed using Bruker TopSpin 3.6.1. Chemical shifts for protons are reported in parts per million (ppm) downfield from tetramethylsilane and are referenced to residual protium in the NMR solvent ($CDCl_3$: $\delta = 7.28$ ppm; D_2O: $\delta = 4.79$ ppm). For ^{13}C NMR, chemical shifts are reported in the scale relative to the NMR solvent (*i.e.* $CDCl_3$: $\delta = 77.00$ ppm). For ^{19}F NMR, chemical shifts are reported in the scale relative to $CFCl_3$. NMR data are reported as follows: chemical shift, multiplicity (s: singlet, d: doublet, dd: doublet of doublets, t: triplet, q: quartet, m: multiplet, br: broad signal), coupling constant (*J* in Hz; accurate to 0.1 Hz), and integration. The number of C-atoms in brackets indicates overlapping signals in ^{13}C NMR; chemical shift numbers in brackets indicate close signals that can be differentiated considering second respectively third decimal numbers.

3.2 General synthetic procedures

3.2.1 General Procedure A

To a solution of mono-methyl dicarboxylic acid half-ester **S1** (1.0 equiv.) and 1-(cyanomethyl)tetrahydro-1*H*-thiophen-1-ium bromide **S2**[2] (1.4 equiv.) in anhydrous dichloromethane (0.2 M) were sequentially added redistilled *N*,*N*-diisopropylethylamine (4.0 equiv.) and T3P (50%$_{w/w}$ in ethyl acetate, 1.4 equiv.) dropwise at 0° C under an atmosphere of N_2 gas. The reaction mixture was stirred and allowed to slowly warm to ambient temperature overnight (10 – 12 h). The reaction mixture was diluted with saturated aqueous $NaHCO_3$ solution and extracted three times with dichloromethane. The combined organic extracts were dried over anhydrous Na_2SO_4, filtered, and evaporated. The crude residue was purified using column chromatography to afford sulfur ylide **S3** which was used in the next reaction following General Procedure B.

3.2.2 General Procedure B

To a solution of the sulfur ylide **S3** (1.0 equiv.) in a 2:1 mixture (0.08 M final concentration) of methanol (HPLC grade) and Milli-Q® ultrapure water was added Oxone (2.0 equiv., obtained from Alfa Aesar as recommended by Bode et al.[2]) under an ambient atmosphere at 0° C. The reaction mixture was stirred vigorously at the same temperature for 5 min, then at ambient temperature for 1 h. It was then filtered and the methanol removed under reduced pressure. The remaining aqueous solution was carefully diluted with saturated aqueous $NaHCO_3$ solution and the mixture extracted three times with dichloromethane. The combined organic extracts were dried over anhydrous Na_2SO_4, filtered, and evaporated. The crude residue was purified using column chromatography to afford dimethyl dicarboxylic acid ester **S4** which was used in the next reaction following General Procedure C.

3.2.3 General Procedure C

To a solution of dimethyl dicarboxylic acid ester **S4** (1.0 equiv.) in methanol (0.2 M, HPLC grade) was added an aqueous solution of lithium hydroxide (0.4 M, 2.8 equiv.) under an ambient atmosphere at 0° C. The reaction mixture was allowed to slowly warm to ambient temperature overnight (14 – 18 h). The methanol was then removed under reduced pressure. The aqueous reaction mixture was extracted three times with dichloromethane (the organic extracts were discarded) and the aqueous phase was acidified (pH ≈ 7.0 to 7.7) using Dowex® 50XW8 (H^+-form, mesh 200–400). The mixture was filtered and lyophilized to afford the solid dicarboxylic acid **S5**. The crude product was sufficiently pure as judged by ^1H and ^{13}C NMR for use in biological assays. pKa-values for the 2OG derivatives were not determined, thus, some might have actually been isolated as the corresponding mono- or dilithium salts. The chemical shift data of the 2OG derivatives are likely pH-dependent. In the ^1H NMR spectrum of 2OG derivative **22** (recorded in D_2O), evidence for H/D-exchange at the methylene group α to the ketone was observed.

3.3 Synthetic procedures and analytical data

1-Benzyl 4-methyl (*E*)-2-((9,9-dimethyl-9*H*-fluoren-2-yl)methylene)succinate (37).

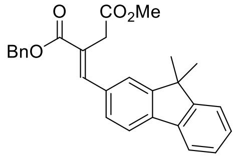

A solution of 1-benzyl-4-methyl itaconate[15] (3.51 g, 15.0 mmol, 1.0 equiv.), 2-iodo-9,9-dimethyl-9*H*-fluorene (5.28 g, 16.5 mmol, 1.1 equiv.), and *N*,*N*-dicyclohexylmethylamine (4.82 mL, 22.5 mmol, 1.5 equiv.) in anhydrous *N*,*N*-dimethylacetamide (10 mL) was added to a sealed 20 mL microwave vial (Biotage) containing palladium acetate (67.4 mg, 0.3 mmol, 0.02 equiv.) and tetraethylammonium chloride (2.49 g, 15.0 mmol, 1.0 equiv.). N_2 gas was bubbled through the reaction mixture for 15 min. The microwave vial was placed in a preheated sand bath (130° C) and the reaction mixture was stirred for 19 h. The reaction mixture was then cooled to ambient temperature and evaporated under reduced pressure. The residue was diluted with saturated aqueous NH_4Cl solution, then extracted three times with ethyl acetate. The combined organic extracts were dried over anhydrous Na_2SO_4, filtered, evaporated, and purified using column chromatography (100 g Ultra cartridge; 60 mL/min; initially, 100% cyclohexane (3 CV), followed by a linear gradient (9 CV): 0%→9% ethyl acetate in cyclohexane) to afford succinate **37** (5.27 g, 82%). The reaction product was isolated as a single alkene diastereoisomer, which was tentatively assigned based on the literature[16] as the *E*-isomer. ^1H and ^{13}C NMR spectra of **37** are shown in Supplementary Figure 34.

Clear yellow oil; ^1H NMR (600 MHz, 300 K, $CDCl_3$): δ = 8.05 (s, 1H), 7.76−7.74 (m, 2H), 7.47−7.40 (m, 6H), 7.38−7.35 (m, 4H), 5.31 (s, 2H), 3.72 (s, 3H), 3.67 (s, 2H), 1.50 ppm (s, 6H); ^{13}C NMR (150 MHz, 300 K, $CDCl_3$): δ = 171.7, 167.3, 154.0, 153.9, 142.9, 140.3, 138.4, 136.0, 133.7, 128.6, 128.3, 128.2, 128.1(8), 127.9, 127.1, 125.1, 123.6, 122.7, 120.4, 120.1, 66.9, 52.1, 46.9, 33.8, 27.0 ppm; IR (film): $\tilde{\nu}$ = 3063, 3035, 2958, 2925, 1738, 1708, 1634, 1449, 1382, 1328, 1289, 1255, 1176, 1096, 1007 cm^{-1}; HRMS (ESI): *m/z* calculated for $C_{28}H_{26}O_4Na$ $[M+Na]^+$: 449.1723, found: 449.1722.

Methyl 5-cyano-3-((9,9-dimethyl-9*H*-fluoren-2-yl)methyl)-4-oxo-5-(tetrahydro-1-λ^4-thiophen-1-ylidene)pentanoate (38).

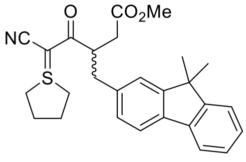

1-Benzyl 4-methyl (*E*)-2-((9,9-dimethyl-9*H*-fluoren-2-yl)methylene)succinate (5.27 g, 12.4 mmol, 1.0 equiv.) was dissolved in anhydrous methanol (35 mL). N_2 gas was bubbled through the solution for 15 min before palladium on charcoal (132 mg of $10\%_{w/w}$ palladium, 1.2 mmol, 0.1 equiv.) was added. H_2 gas was bubbled through the black suspension for 15 min and the reaction mixture was stirred for 19 h at ambient temperature under an atmosphere of H_2 (1 atm). The resultant black suspension was filtered through Celite®, washed with methanol (*Caution*: Palladium-containing waste was kept as aqueous suspension before appropriate disposal), and evaporated to afford the corresponding crude mono-methyl dicarboxylic acid half-ester (83%), which was used in the next reaction without further purification: According to General Procedure A, cyanosulfur ylide **38** (2.83 g, 52% over two steps) was obtained, following column chromatography (100 g KP-Sil cartridge; 60 mL/min; initially, 100% ethyl acetate (8 CV), followed by a linear gradient (7 CV): 0%→100% acetone in ethyl acetate). ^1H and ^{13}C NMR spectra of **38** are shown in Supplementary Figure 35.

Clear orange oil; ^1H NMR (600 MHz, 300 K, $CDCl_3$): δ = 7.70−7.69 (m, 2H), 7.63 (d, *J* = 7.7 Hz, 1H), 7.44−7.43 (m, 1H), 7.44−7.43 (m, 1H), 7.35−7.29 (m, 3H), 7.22 (dd, *J* = 7.7, 1.3 Hz, 1H), 3.66−3.61 (m, 4H), 3.28−3.22 (m, 3H), 3.16 (dt, *J* = 12.6, 6.8 Hz, 1H), 3.04 (dd, *J* = 13.4, 7.4 Hz, 1H), 2.81 (dd, *J* = 16.4, 9.5 Hz, 1H), 2.77 (dd, *J* = 13.4, 8.2 Hz, 1H), 2.60−2.54 (m, 1H), 2.52−2.47 (m, 1H), 2.43 (dd, *J* = 16.4, 4.8 Hz, 1H), 2.04−1.95 (m, 2H), 1.51 (s, 3H), 1.49 ppm (s, 3H); ^{13}C NMR (150 MHz, 300 K, $CDCl_3$): δ = 192.5, 172.5, 153.8, 153.7, 139.1, 138.1, 137.5, 128.2, 126.9, 126.8, 123.5, 122.5, 120.3, 119.8, 119.7, 54.8, 51.5, 46.8, 45.3, 44.6, 44.5, 39.3, 35.7, 28.4, 28.3(8), 27.3, 27.1 ppm; IR (film): $\tilde{\nu}$ = 3502, 3009, 2956, 2924, 2862, 2168, 1733, 1591, 1448, 1354, 1301, 1268, 1201, 1153, 1005, 912 cm^{-1}; HRMS (ESI): *m/z* calculated for $C_{27}H_{30}O_3NS$ $[M+H]^+$: 448.1941, found: 448.1942.

Dimethyl 3-((9,9-dimethyl-9*H*-fluoren-2-yl)methyl)-2-oxopentanedioate (39).

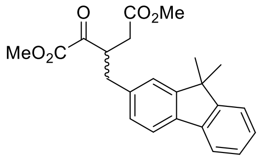

According to General Procedure B, dimethyl dicarboxylate **39** (1.73 g, 72%) was obtained from cyanosulfur ylide **38** (2.83 g, 6.3 mmol), following column chromatography (50 g KP-Sil cartridge; 50 mL/min; initially, 100% cyclohexane (3 CV), followed by a linear gradient (8 CV): 0%→15% ethyl acetate in cyclohexane). ^{1}H and ^{13}C NMR spectra of **39** are shown in Supplementary Figure 36.

Clear yellow oil; ^{1}H NMR (600 MHz, 300 K, $CDCl_3$): δ = 7.71−7.70 (m, 1H), 7.66 (d, J = 7.7 Hz, 1H), 7.45−7.43 (m, 1H), 7.36−7.31 (m, 2H), 7.24 (d, J = 1.0 Hz, 1H), 7.16 (dd, J = 7.7, 1.5 Hz, 1H), 4.07 (dddd, J = 10.3, 8.7, 6.1, 4.4 Hz, 1H), 3.84 (s, 3H), 3.63 (s, 3H), 3.15 (dd, J = 13.7, 6.3 Hz, 1H), 2.88 (dd, J = 17.3, 10.2 Hz, 1H), 2.74 (dd, J = 13.7, 8.6 Hz, 1H), 2.57 (dd, J = 17.3, 4.4 Hz, 1H), 1.49 (s, 3H), 1.48(7) ppm (s, 3H); ^{13}C NMR (150 MHz, 300 K, $CDCl_3$): δ = 195.6, 172.2, 161.0, 154.2, 153.6, 138.7, 138.1, 136.4, 127.9, 127.2, 127.0, 123.3, 122.6, 120.1, 119.9, 53.0, 52.0, 46.8, 45.0, 37.3, 35.0, 27.1, 27.0(8) ppm; IR (film): ṽ = 3008, 2957, 2924, 2861, 1731, 1438, 1360, 1264, 1205, 1101, 1047 cm^{-1}; HRMS (ESI): *m/z* calculated for $C_{23}H_{24}O_5Na$ [M+Na]$^+$: 403.1516, found: 403.1518.

3-((9,9-Dimethyl-9*H*-fluoren-2-yl)methyl)-2-oxopentanedioic acid (11).

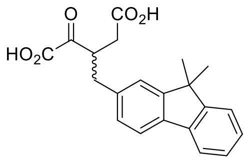

Dicarboxylic acid **11** (42 mg, 60%) was obtained from dimethyl 3-((9,9-dimethyl-9*H*-fluoren-2-yl)methyl)-2-oxopentanedioate **39** (76.1 mg, 0.2 mmol) according to General Procedure C. ^{1}H and ^{13}C NMR spectra of **11** are shown in Supplementary Figure 37.

Pale yellow solid, m.p.: >220 °C (decomposition); ^{1}H NMR (600 MHz, 300 K, D_2O): δ = 7.89−7.87 (m, 1H), 7.82 (d, J = 7.7 Hz, 1H), 7.64−7.63 (m, 1H), 7.50 (d, J = 1.0 Hz, 1H), 7.47−7.43 (m, 2H), 7.30 (dd, J = 7.7, 1.5 Hz, 1H), 3.85 (apparent pent., J = 7.0 Hz, 1H), 3.16 (dd, J = 14.0, 6.9 Hz, 1H), 2.93 (dd, J = 14.0, 6.9 Hz, 1H), 2.55 (dd, J = 15.8, 7.5 Hz, 1H), 2.31 (dd, J = 15.8, 6.8 Hz, 1H), 1.50 ppm (s, 6H); ^{13}C NMR (150 MHz, 300 K, D_2O): δ = 207.7, 180.2, 169.9, 154.2, 154.1, 138.7, 138.4, 136.8, 128.4, 127.6, 127.4, 123.9, 123.0, 120.1, 120.0, 46.8, 46.5, 37.3, 35.9, 26.1, 26.0(6) ppm; IR (film): ṽ = 2962, 2928, 1700, 1636, 1577, 1406, 1361, 1301, 1216, 1107 cm^{-1}; HRMS (ESI): *m/z* calculated for $C_{21}H_{19}O_5$ [M−H]$^-$: 351.1238, found: 351.1237.

Methyl (1*R*,3*S*)-3-(2-cyano-2-(tetrahydro-1l4-thiophen-1-ylidene)acetyl)cyclopentane-1-carboxylate (40).

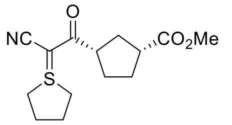

According to General Procedure A, cyanosulfur ylide **40** (1.38 g, 85%) was obtained from commercially-sourced (1*R*,3*S*)-3-(methoxycarbonyl)cyclopentane-1-carboxylic acid (993 mg, 5.8 mmol), following column chromatography (50 g KP-Sil cartridge; 50 mL/min; initially, 100% ethyl acetate (7 CV), followed by a linear gradient (7 CV): 0%→100% acetone in ethyl acetate). ^{1}H and ^{13}C NMR spectra of **40** are shown in Supplementary Figure 38.

Clear pale orange oil; ^{1}H NMR (600 MHz, 300 K, $CDCl_3$): δ = 3.68 (s, 3H), 3.39−3.35 (m, 4H), 3.23 (ddt, J = 9.9, 8.0, 7.9 Hz, 1H), 2.81 (ddt, J = 10.0, 8.0, 8.0 Hz, 1H), 2.66−2.60 (m, 2H), 2.22 (dt, J = 12.8, 7.6 Hz, 1H), 2.10−1.89 ppm (m, 7H); ^{13}C NMR (150 MHz, 300 K, $CDCl_3$): δ = 193.4, 175.9, 121.0, 53.0, 51.6, 47.2, 44.7, 44.6, 44.1, 33.8, 29.2(4), 29.2, 28.4 ppm; IR (film): ṽ = 3485, 2952, 2873, 2166, 1729, 1591, 1438, 1369, 1309, 1272, 1206, 1154, 1085 cm^{-1}; HRMS (ESI): *m/z* calculated for $C_{14}H_{19}O_3NSNa$ [M+Na]$^+$: 304.0978, found: 304.0978.

Methyl (1*R*,3*S*)-3-(2-methoxy-2-oxoacetyl)cyclopentane-1-carboxylate (41).

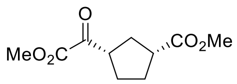

According to General Procedure B, dimethyl dicarboxylate **41** (536 mg, 50%) was obtained from cyanosulfur ylide **40** (1.38 g, 5.0 mmol), following column chromatography (25 g KP-Sil cartridge; 45 mL/min; initially, 100% cyclohexane (3 CV),

followed by a linear gradient (5 CV): 0%→20% ethyl acetate in cyclohexane). ^{1}H and ^{13}C NMR spectra of **41** are shown in Supplementary Figure 39.

Clear colorless oil; ^{1}H NMR (600 MHz, 300 K, $CDCl_3$): δ = 3.89 (s, 3H), 3.70 (s, 3H), 3.59−3.54 (m, 1H), 2.91−2.85 (m, 1H), 2.27 (dt, J = 13.3, 8.2 Hz, 1H), 2.16 (dt, J = 13.3, 8.6 Hz, 1H), 2.04−1.91 ppm (m, 4H); ^{13}C NMR (150 MHz, 300 K, $CDCl_3$): δ = 194.6, 175.2, 161.8, 52.9, 51.8, 47.3, 43.9, 31.5, 29.3, 27.5 ppm; IR (film): ṽ = 2956, 2880, 1729, 1437, 1367, 1272, 1207, 1168, 1097, 1048 cm^{-1}; HRMS (ESI): *m/z* calculated for $C_{10}H_{14}O_5Na$ $[M+Na]^+$: 237.0733, found: 237.0736.

(1*R*)-3-(Carboxycarbonyl)cyclopentane-1-carboxylic acid (22).

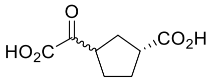

Dicarboxylic acid **22** (93 mg, quant.) was obtained as a mixture of C3 diastereomers ($dr_{cis/trans}$ = 1:1), which were not separated, from (1*R*,3*S*)-methyl 3-(2-methoxy-2-oxoacetyl)cyclopentane-1-carboxylate **41** (107 mg, 0.5 mmol) according to General Procedure C. H/D-exchange at the methylene group α to the ketone was observed by ^{1}H NMR in D_2O, ^{1}H and ^{13}C NMR spectra of **22** are shown in Supplementary Figure 40.

White amorphous solid; ^{1}H NMR (600 MHz, 300 K, D_2O; 1:1 mixture of racemic diastereomers): δ = 3.51−3.46 (m, 1H), 3.43−3.38 (m, 1H), 2.87−2.82 (m, 1H), 2.81−2.76 (m, 1H), 2.24 (dt, J = 13.1, 8.1 Hz, 1H), 2.18−2.13 (m, 1H), 2.11−2.04 (m, 2H), 2.03−1.97 (m, 3H), 1.95−1.86 (m, 2H), 1.83−1.74 ppm (m, 3H); ^{13}C NMR (150 MHz, 300 K, D_2O; 1:1 mixture of racemic diastereomers): δ = 208.7, 208.4, 183.2, 182.8, 171.7, 171.6(7), 47.5, 47.2, 45.9, 45.3, 31.7, 31.1, 30.1, 29.3, 27.9, 27.4 ppm; IR (film): ṽ = 3074, 2972, 2880, 1698, 1600, 1407, 1317, 1193, 1142, 1058 cm^{-1}; HRMS (ESI): *m/z* calculated for $C_8H_9O_5$ $[M-H]^-$: 185.0455, found: 185.0447.

4. Supplementary References

1. Brewitz, L., Nakashima, Y. & Schofield, C. J. Synthesis of 2-oxoglutarate derivatives and their evaluation as cosubstrates and inhibitors of human aspartate/asparagine-β-hydroxylase. *Chem. Sci.* **12**, 1327-1342 (2021).
2. Ju, L., Lippert, A. R. & Bode, J. W. Stereoretentive synthesis and chemoselective amide-forming ligations of C-terminal peptide α-ketoacids. *J. Am. Chem. Soc.* **130**, 4253-4255 (2008).
3. Kelly, L., McDonough, M. A., Coleman, M. L., Ratcliffe, P. J. & Schofield, C. J. Asparagine β-hydroxylation stabilizes the ankyrin repeat domain fold. *Mol. BioSyst.* **5**, 52-58 (2009).
4. Koivunen, P., Hirsilä, M., Günzler, V., Kivirikko, K. I. & Myllyharju, J. Catalytic properties of the asparaginyl hydroxylase (FIH) in the oxygen sensing pathway are distinct from those of its prolyl 4-hydroxylases. *J. Biol. Chem.* **279**, 9899-9904 (2004).
5. McDonough, M. A., et al. Selective inhibition of factor inhibiting hypoxia-inducible factor. *J. Am. Chem. Soc.* **127**, 7680-7681 (2005).
6. Copeland, R. A. *Enzymes: A practical introduction to structure, mechanism, and data analysis*. Wiley-VCH (2000).
7. Wu, Y., Li, Z., McDonough, M. A., Schofield, C. J. & Zhang, X. Inhibition of the oxygen-sensing asparaginyl hydroxylase factor inhibiting hypoxia-inducible factor: A potential hypoxia response modulating strategy. *J. Med. Chem.* **64**, 7189-7209 (2021).
8. Pfeffer, I., et al. Aspartate/asparagine-β-hydroxylase crystal structures reveal an unexpected epidermal growth factor-like domain substrate disulfide pattern. *Nat. Commun.* **10**, 4910 (2019).
9. Elkins, J. M., et al. Structure of factor-inhibiting hypoxia-inducible factor (HIF) reveals mechanism of oxidative modification of HIF-1α. *J. Biol. Chem.* **278**, 1802-1806 (2003).
10. Yang, M., et al. Factor-inhibiting hypoxia-inducible factor (FIH) catalyses the post-translational hydroxylation of histidinyl residues within ankyrin repeat domains. *FEBS J.* **278**, 1086-1097 (2011).
11. Zhang, J.-H., Chung, T. D. Y. & Oldenburg, K. R. A simple statistical parameter for use in evaluation and validation of high throughput screening assays. *J. Biomol. Screen.* **4**, 67-73 (1999).
12. Choi, H., et al. A human protein hydroxylase that accepts D-residues. *Commun. Chem.* **3**, 52 (2020).
13. Brewitz, L., Tumber, A., Pfeffer, I., McDonough, M. A. & Schofield, C. J. Aspartate/asparagine-β-hydroxylase: a high-throughput mass spectrometric assay for discovery of small molecule inhibitors. *Sci. Rep.* **10**, 8650 (2020).
14. Rose, N. R., et al. Inhibitor scaffolds for 2-oxoglutarate-dependent histone lysine demethylases. *J. Med. Chem.* **51**, 7053-7056 (2008).
15. Sagot, E., et al. Chemo-enzymatic synthesis of a series of 2,4-syn-functionalized (S)-glutamate analogues: New insight into the structure−activity relation of ionotropic glutamate receptor subtypes 5, 6, and 7. *J. Med. Chem.* **51**, 4093-4103 (2008).
16. Gürtler, C. & Buchwald, S. L. A phosphane-free catalyst system for the Heck arylation of disubstituted alkenes: Application to the synthesis of trisubstituted olefins. *Chem. Eur. J.* **5**, 3107-3112 (1999).
