# Supplementary material for: 2-Oxoglutarate derivatives can selectively enhance or inhibit the activity of human oxygenases
Source: Nat Commun. 2021 Nov 10;12:6478. doi: 10.1038/s41467-021-26673-2 (PMC8580996; doi:10.1038/s41467-021-26673-2)
Supplement: Supplementary file 2 — Reporting Summary [file 41467_2021_26673_MOESM2_ESM.pdf]

nature research

Corresponding author(s): Prof. Dr. Christopher J. Schofield FRS

Last updated by author(s): Sep 16, 2021

Reporting Summary

Nature Research wishes to improve the reproducibility of the work that we publish. This form provides structure for consistency and transparency in reporting. For further information on Nature Research policies, see our [Editorial Policies] and the [Editorial Policy Checklist].

Statistics

For all statistical analyses, confirm that the following items are present in the figure legend, table legend, main text, or Methods section.

n/a	Confirmed	
☐	☒	The exact sample size (n) for each experimental group/condition, given as a discrete number and unit of measurement
☐	☒	A statement on whether measurements were taken from distinct samples or whether the same sample was measured repeatedly
☒	☐	The statistical test(s) used AND whether they are one- or two-sided<br>*Only common tests should be described solely by name; describe more complex techniques in the Methods section.*
☒	☐	A description of all covariates tested
☒	☐	A description of any assumptions or corrections, such as tests of normality and adjustment for multiple comparisons
☐	☒	A full description of the statistical parameters including central tendency (e.g. means) or other basic estimates (e.g. regression coefficient) AND variation (e.g. standard deviation) or associated estimates of uncertainty (e.g. confidence intervals)
☒	☐	For null hypothesis testing, the test statistic (e.g. F, t, r) with confidence intervals, effect sizes, degrees of freedom and P value noted<br>*Give P values as exact values whenever suitable.*
☒	☐	For Bayesian analysis, information on the choice of priors and Markov chain Monte Carlo settings
☒	☐	For hierarchical and complex designs, identification of the appropriate level for tests and full reporting of outcomes
☒	☐	Estimates of effect sizes (e.g. Cohen's d, Pearson's r), indicating how they were calculated

Our web collection on statistics for biologists contains articles on many of the points above.

Software and code

Policy information about availability of computer code

Data collection	Mass spectrometry: Agilent MassHunter workstation B.08.00; NMR spectrometry: Bruker TopSpin 3.6.1; Crystallography: the Diamond Light Source beamline auto-processing pipeline software packages autoPROC, STARANISO, and Xia2 were used for automated data indexing, integration, and scaling according to standard beamline protocols. Citations are provided in the manuscript.
Data analysis	Mass spectrometry: Agilent RapidFire Integrator 4.3.0, Microsoft Excel, and GraphPad Prism 5.04; NMR spectroscopy: Bruker TopSpin 3.6.1; Crystallography: PHENIX 1.18.2 software suite which includes PHASER, PHENIX.refine, and Polder Maps programs; as well as Coot 0.8.6.1, and Pymol 4.6. Citations for the specific software used have been provided in the manuscript or Supplementary Information.

For manuscripts utilizing custom algorithms or software that are central to the research but not yet described in published literature, software must be made available to editors and reviewers. We strongly encourage code deposition in a community repository (e.g. GitHub). See the Nature Research guidelines for submitting code & software for further information.

Data

Policy information about availability of data

All manuscripts must include a data availability statement. This statement should provide the following information, where applicable:

- Accession codes, unique identifiers, or web links for publicly available datasets
- A list of figures that have associated raw data
- A description of any restrictions on data availability

Crystal structure data for FIH complexed to Zn, 2OG derivative, and, if applicable, substrate peptide (CA1-20 or TANK2691-710) are deposited in the protein data bank with PDB accession codes: 7A1L [http://doi.org/10.2210/pdb7a1l/pdb] (FIH:1), 7A1M [http://doi.org/10.2210/pdb7a1m/pdb] (FIH:3), 7A1J [http://doi.org/10.2210/pdb7a1j/pdb] (FIH:18), 7A1K [http://doi.org/10.2210/pdb7a1k/pdb] (FIH:11), 7A1N [http://doi.org/10.2210/pdb7a1n/pdb] (FIH:1:CA1-20), 7A1O [http://doi.org/10.2210/pdb7a1o/pdb] (FIH:14:CA1-20), 7A1P [http://doi.org/10.2210/pdb7a1p/pdb] (FIH:15:CA1-20), 7A1Q [http://doi.org/10.2210/pdb7a1q/pdb]

(FIH:22:CA1-20), and 7A1S [http://doi.org/10.2210/pdb7a1s/pdb] (FIH:1:TANK2691-710). Additionally, data of reported crystal structure have been used: 1H2L [http://doi.org/10.2210/pdb1h2l/pdb], 1H2K [http://doi.org/10.2210/pdb1h2k/pdb], 1YCI [http://doi.org/10.2210/pdb1yci/pdb], and 6YYX [http://doi.org/10.2210/pdb6yyx/pdb], for which references are given in the manuscript. Source data are provided with this paper and can be obtained from the authors.

Field-specific reporting

Please select the one below that is the best fit for your research. If you are not sure, read the appropriate sections before making your selection.

[x] Life sciences [ ] Behavioural & social sciences [ ] Ecological, evolutionary & environmental sciences

For a reference copy of the document with all sections, see nature.com/documents/nr-reporting-summary-flat.pdf

Life sciences study design

All studies must disclose on these points even when the disclosure is negative.

Sample size	No sample size calculations were performed for mass spectrometry and NMR assays or during chemical synthesis. For crystallography, the Diamond Light Source beamline auto-processing pipeline software packages autoPROC, STARANISO, and Xia2 were used for automated data indexing, integration, and scaling according to standard beamline protocols, detailed crystallographic parameters are are given in Supplementary Tables 2 and 3.
Data exclusions	The vast majority of data generated from independent assay repeats were included in the data analysis as shown in the Supplementary Information and Source Data File and by error bars in the manuscript figures. In rare cases (<5%), independent replicates were not successful and data was excluded from the analysis as shown in the Source Data File; experiments failed mostly due to human-caused errors in experimental set up. Details on crystallographic data handling are provided in the Methods section.
Replication	Mass spectrometry assays were run in independent replicates ($n \geq 2$), as specified in the manuscript and Supplementary Information. The vast majority of the independent replication attempts (>95%) were successful; experiments failed mostly due to human-caused errors in experimental set up.
Randomization	Samples were not randomized for the in vitro mass spectrometry, NMR spectroscopy, crystallographic, and the chemical synthesis assays and experiments described. The order of adding components for mass spectrometry/NMR assays and crystallization experiments as well as chemicals during synthetic experiments are specified in the Methods section or Supplementary Information.
Blinding	For the in vitro mass spectrometry, NMR spectroscopy, crystallographic, and the chemical synthesis experiments described in the manuscript, blinding was not feasible. Unblinded control experiments have been performed for all assays.

Reporting for specific materials, systems and methods

We require information from authors about some types of materials, experimental systems and methods used in many studies. Here, indicate whether each material, system or method listed is relevant to your study. If you are not sure if a list item applies to your research, read the appropriate section before selecting a response.

Materials & experimental systems

n/a	Involved in the study	
[x]	[ ]	Antibodies
[x]	[ ]	Eukaryotic cell lines
[x]	[ ]	Palaeontology and archaeology
[x]	[ ]	Animals and other organisms
[x]	[ ]	Human research participants
[x]	[ ]	Clinical data
[x]	[ ]	Dual use research of concern

Methods

n/a	Involved in the study	
[x]	[ ]	ChIP-seq
[x]	[ ]	Flow cytometry
[x]	[ ]	MRI-based neuroimaging
